# Pathophysiological Changes in the Enteric Nervous System of Rotenone-Exposed Mice as Early Radiological Markers for Parkinson's Disease

**DOI:** 10.3389/fneur.2021.642604

**Published:** 2021-03-22

**Authors:** Gabriela Schaffernicht, Qi Shang, Alicia Stievenard, Kai Bötzel, Yanina Dening, Romy Kempe, Magali Toussaint, Daniel Gündel, Mathias Kranz, Heinz Reichmann, Christel Vanbesien-Mailliot, Peter Brust, Marianne Dieterich, Richard H. W. Funk, Ursula Ravens, Francisco Pan-Montojo

**Affiliations:** ^1^Department of Neurology, University Hospital, Ludwig-Maximilians-University (LMU) Munich, Munich, Germany; ^2^Munich Cluster for Systems Neurology, SyNergy, Munich, Germany; ^3^Univ. Lille, Inserm, CHU Lille, UMR-S 1172 - JPArc - Centre de Recherche Jean-Pierre AUBERT Neurosciences et Cancer, Lille, France; ^4^Department of Pharmacology and Toxicology, TU-Dresden, Dresden, Germany; ^5^Helmholtz-Zentrum Dresden-Rossendorf, Institute of Radiopharmaceutical Cancer Research, Leipzig, Germany; ^6^Department of Neurology, Technische Universität Dresden, Dresden, Germany; ^7^Center for Regenerative Therapies Dresden, Dresden, Germany; ^8^German Center for Vertigo and Balance Disorders, University Hospital, LMU Munich, Munich, Germany; ^9^Institute for Anatomy, Technical University (TU)-Dresden, Dresden, Germany; ^10^Institute for Experimental Cardiovascular Medicine, University Heart Center, Medical Center – University of Freiburg, Faculty of Medicine, University of Freiburg, Freiburg, Germany

**Keywords:** Parkinson's disease, non-motor symptoms, enteric nervous system, pathophysiology, radiological marker

## Abstract

Parkinson's disease (PD) is known to involve the peripheral nervous system (PNS) and the enteric nervous system (ENS). Functional changes in PNS and ENS appear early in the course of the disease and are responsible for some of the non-motor symptoms observed in PD patients like constipation, that can precede the appearance of motor symptoms by years. Here we analyzed the effect of the pesticide rotenone, a mitochondrial Complex I inhibitor, on the function and neuronal composition of the ENS by measuring intestinal contractility in a tissue bath and by analyzing related protein expression. Our results show that rotenone changes the normal physiological response of the intestine to carbachol, dopamine and electric field stimulation (EFS). Changes in the reaction to EFS seem to be related to the reduction in the cholinergic input but also related to the noradrenergic input, as suggested by the non-adrenergic non-cholinergic (NANC) reaction to the EFS in rotenone-exposed mice. The magnitude and direction of these alterations varies between intestinal regions and exposure times and is associated with an early up-regulation of dopaminergic, cholinergic and adrenergic receptors and an irregular reduction in the amount of enteric neurons in rotenone-exposed mice. The early appearance of these alterations, that start occurring before the *substantia nigra* is affected in this mouse model, suggests that these alterations could be also observed in patients before the onset of motor symptoms and makes them ideal potential candidates to be used as radiological markers for the detection of Parkinson's disease in its early stages.

## Introduction

Hallmark lesions of Parkinson's disease (PD) were traditionally considered to be present in the dopaminergic neurons of the substantia nigra (SN) and in the noradrenergic neurons of the locus coeruleus (LC). However, pathological studies showed that PD patients typically have lesions in other central nervous system (CNS) and PNS structures (e.g., the ENS, the sympathetic coeliac ganglion (CG), the intermediolateral nucleus (IML) of the spinal cord, the motor nucleus of the vagus DMV or the amygdala) (1, 2). These lesions are not exclusive of PD (3) and mainly consist in intraneuronal and intraglial alpha-synuclein aggregates called Lewy bodies (LB) and Lewy neurites (LN).

In idiopathic PD most patients showing PD-related inclusions in CNS sites also present LB and LN in the ENS and the sympathetic ganglia (4). Based on autopsies performed on PD patients and healthy individuals, Braak et al. proposed a pathological staging of the disease (5). According to this staging, PD lesions follow a spatio-temporal pattern that starts in the olfactory bulb (OB) and the ENS progressing into the CNS through synaptically connected structures. This pathological staging of the disease seems to correlate well with the appearance of early non-motor symptoms in PD patients including hyposmia, gastrointestinal alterations, autonomic dysfunction, and pain (6).

Little is known about the degenerative process of the ENS in PD patients. Physiological studies in patients revealed delayed gastric emptying, dystonia of the external anal sphincter that causes difficult rectal evacuation and general slow transit constipation probably caused by the local loss of dopaminergic neurons (7–9). In recent years many groups have studied expression and modification of enteric alpha-synuclein in PD patients, with controversial results. Whilst some found an increase in alpha-synuclein inclusions and phosphorylated alpha-synuclein in the above-mentioned areas when compared to control subjects (10), there appears to be a high variability between patients. A review of the literature reveals that not many studies investigated PD-associated changes in the function of the ENS, its neuronal composition or its extrinsic innervation.

In previous studies, we have shown that chronic oral administration of rotenone in mice induces alpha-synuclein accumulation in most of the regions described in Braak's staging (11, 12). The appearance of these alterations followed a spatio-temporal pattern similar to the one predicted by Braak et al. and the disruption of the autonomic nerves connecting the gut to the CNS (i.e., the vagus and sympathetic nerves) prevented the progression of the pathology. Interestingly, we also observed gastrointestinal alterations in rotenone-exposed mice in the form of constipation and a reduction of the noradrenergic innervation of the gut (13). An independent recent study showed alterations in the extrinsic cholinergic innervation of the gut after rotenone exposure in guinea pigs (14). However, there is no study describing the effect that these rotenone-induced pathological changes have on the function of the ENS and more specifically on its role in the regulation of the gastrointestinal contractility. Normal physiological functions of the ENS include: determining the patterns of movement of the gastrointestinal tract; controlling gastric acid secretion; regulating movement of fluid across the lining epithelium; changing local blood flow; modifying nutrient handling; and interacting with the immune and endocrine systems of the gut. The ENS also contributes, along with glial cells, to maintaining the integrity of the epithelial barrier between the gut lumen and cells and tissues within the gut wall. To determine the patterns of movement of the gastrointestinal tract, the ENS has to process the sensory input from mechanical and chemical receptors in the intestinal wall and integrate the sympathetic and parasympathetic input to generate a coordinated effect [reviewed in (15)]. All these functions are based on the interaction between different neuronal subtypes in the ENS, the balance between the type of input that each neurotransmitter released puts in the neuronal network and the expression levels of the post-synaptic receptors. In this context, the current study was designed to further investigate the consequences of orally administered rotenone on the ENS, its neuronal subpopulations, the balance between neurotransmitters, the expression of postsynaptic receptors and its regulation of gastrointestinal contractility (GC). Our results show that chronic rotenone administration in mice affects the electrophysiological properties of the gut, that these alterations correlate well with the up-regulation of certain post-synaptic receptors, and that this is most probably due to the reduction of the extrinsic input from the sympathetic and vagal innervation. This could be important to identify radiological markers for the early diagnosis of PD.

## Materials and Methods

### Animal Model

#### Animal Housing

One year-old C57/BL6J mice (Janvier, France) were housed at room temperature under a 12-h light/dark cycle. Food and water were provided *ad libidum*. All animal experiments were carried out in accordance with the National Institutes of Health Guide for the Care and Use of Laboratory Animals, and protocols were approved by the Saxonian Committee for Animal Research in Germany.

### Chronic Oral Rotenone Administration

Chronic oral exposure to low doses of the pesticide rotenone was performed as previously described (11). Briefly, 1 year-old mice were divided into 2 groups (*n* = 10) and exposed 5 days a week for up to 2 or 4 months (total amount of animals *n* = 40). A 1.2 × 60 mm gavage needle (Unimed, Switzerland) was used to administer 0.01 mL/g animal weight of rotenone solution corresponding to a 5 mg/kg dose. Controls were exposed only with the vehicle solution [2% carboxymethylcellulose (Sigma-Aldrich, Germany) and 1.25% chloroform (Carl Roth, Germany)]. For the PET experiments, additional animals were included in the study. The first experimental series (age = 14 months; weight = 28–35 g), was composed of a control group (*n* = 5) and an exposed group (*n* = 6), respectively unexposed or exposed for 2 months to rotenone. The second experimental series (age = 16 months; weight = 28–35 g), was composed of a control group (*n* = 2) and a exposed group (*n* = 5), respectively unexposed or exposed for 4 months to rotenone.

### Tissue Preparation for Organ Bath and Western Blot

Mice were killed with an overdose of ketamine. Several 1 cm long adjacent intestinal segments were removed from each region (duodenum, jejunum, ileum, and colon), submerged in Krebs solution [120 mM (NaCl), 5.8 mM (KCl), 2.5 mM (CaCl_2_), 1.2 mM (MgCl_2_), 1.4 mM (NaH_2_PO_4_), 15 mM (NaHCO_3_), 11 mM (glucose)], and gently flushed to remove luminal contents. The segments were used for contractility studies in an organ bath or immediately frozen in liquid nitrogen until protein extraction.

### Contractility Studies in an Organ Bath

In order to test the motility of the intestinal segments we measured contractility using an isolated organ bath (Glasapparatemeister, Topas GmbH, Germany) as performed by others (16). The tissue pieces were ligated at each end with a silk thread and suspended longitudinally between two platinum electrodes by attaching one end to the isometric force transducer (Transducer Typ SG4-45, SWEMA, Sweden) and the other end to the base of the 15 ml chamber filled with Krebs solution maintained at 37°C and aerated with 95% O_2_/5%CO_2_. Samples were stabilized in the Krebs solution for 45 min. During this time the bath solution was changed twice and samples were pre-loaded to 10 mN by step-wise increases until the tension was adjusted at 10 mN. Spontaneous smooth muscle activity (SMA) and responses to electrical field stimulation (EFS) were measured isometrically. The mechanical activity of the muscles was recorded using a transducer amplifier relayed to a bioelectric amplifier (Multiplexing Pulse Booster 3165, Gemonio-Varese, Italy) equipped with the data acquisition Chart 4.0TM (AD Instruments, Sydney, NSW, Australia). The preparations were allowed to stabilize for 45 min. During this period the Krebs solution in the bath was exchanged two times. After this equilibration period, SMA was characterized for 15 min by recording basal tone (minimum force during contraction), contraction amplitude (difference between maximum and minimum force during a contraction), and frequency of contractions (number of spontaneous contractions per minute). The preparations were then exposed to different concentrations (in Mol/L: 10^−6^, 3 × 10^−6^, 10^−5^, 3 × 10^−5^, 10^−4^, 3 × 10^−4^, 10^−3^) of the neurotransmitters dopamine and noradrenaline or to the unselective muscarinic receptor agonist carbachol, and the responses of the three contractile parameters were determined. After several medium changes, samples were subjected to EFS using an electric stimulator (Föhr Medical Instruments, Seeheim/Ober Beerbach, Germany). The optimal EFS parameters were determined testing several values for voltage (5, 10, and 15 V) and frequencies (10, 16, and 80 Hz) on test samples. We decided to stimulate the ENS from 2-months exposed mice using three different pulse durations (200, 400, and 800 μs) and reduced the number to two different pulse durations (200 and 400 μs) for the 4-months exposed mice. In all cases a train duration of 30 s with a voltage of 10 V, a pulse delay of 0 s and a pulse rate of 16 Hz were used. Responses to EFS were determined after the last change of bath solution in absence of any additional drugs and represented the responses to stimulation of ENS alone. In order to study the contribution of various neurotransmitters in ENS activation the following drugs were added in top of each other: atropine (AT, 10^−5^ Mol/L) for blocking cholinergic receptors, plus guanethidine (G, 10^−5^ Mol/L) to block additional noradrenergic receptors, plus serotonin (S, 10^−4^ Mol/L) to analyse the effect of serotonin in the absence of cholinergic and adrenergic inputs and plus tetrodotoxin (TTX, 10^−6^ Mol/L) to additionally block voltage-gated sodium channels.

Contractility data for each time-point was translated into a curve by the data acquisition system. The contractile parameters maximum force, minimum force, amplitude and frequency of spontaneous contractions before and after each exposure were automatically extracted using a self-made program written with Matlab (version R2014b, Mathworks, Massachusetts, USA). The effects for each drug concentration on the contractile parameters were determined by normalizing the drug-induced changes to pre-drug control values (time 0). These values were used to generate concentration-response curves for each parameter.

### Protein Extraction for Western Blot

Protein extraction was done in an ice-cold Eppendorf holder using ice cold protein extraction buffer (60 mMol/L TrisHCl (pH 6.8), 1 mMol/L Na_3_VO_4_, 1% SDS) (300 μL). Intestine samples (*n* = 5 per experimental group) were cut into smaller pieces, homogenized with the help of a homogenizing pestle in 1.5 mL Eppendorf tubes and an ultrasound tip. The homogenized sample was kept at 99°C for 5 min and centrifuged at 1,400 rpm for 5 min. The supernatant was collected and stored at −80°C until use.

### SDS-PAGE and Western Blotting

Protein concentration from intestine extracts was measured using the Nanodrop X100. Samples were then diluted in each case to have the same end protein concentration of 30 μg in 20 μL and protein extract, and were loaded together with 5 μL of loading buffer (Invitrogen, Germany, EU) into a 12% polyacrylamide gel.

Page ruler 10–250 kDa molecular weight marker was used for size estimation. Using a Mini Gel Tank (Life Technologies, USA), proteins were separated by electrophoresis using 80 V (stacking) and 120 V (migration) and transferred onto a nitrocellulose membrane using 10 V for 3 h. To verify the efficiency of the transfer, membranes were incubated in a Red Ponceau solution during 2 min and washed three times (3 × 5 min) in deionized water. Membranes were blocked in a blocking solution containing Tris-buffered saline + 0.1% Tween 20 (TBST 1X) and 5% (w/v) non-fat dry milk for 1 h. Membranes were then incubated overnight at 4°C with one of the following primary antibodies: rabbit anti-tyrosine hydroxylase (1:1000, Pel Freez), mouse anti-GAPDH (1:1000, Thermo Scientific), rabbit anti-ChAT (1:1000, Abcam), rabbit anti-Muscarinic Acetylcholine Receptor (M3, 1:3000, ab126168, Abcam), rabbit anti-Dopamine receptor D2 antibody (ADI-905-740-100, 1:1000, Enzo Life Science), or rabbit anti PGP9.5 (AB1761, 1:1000, Millipore or 1:3000, Enzo life Science). On the following day, blots were washed 3 × 5 min. with TBST 1X and incubated with horseradish peroxidase-conjugated anti-rabbit or anti-mouse secondary antibodies (1:5000, Abcam) at room temperature for 1 h.

The blots were subsequently washed 3 × 5 min in TBST 1X and developed with the Prime Western Blotting detection Reagent (GE Healthcare Amersham) using the LAS3000 Bioimager (Fujifilm, Germany, EU). Images were then processed using the open access ImageJ based program FIJI (ww.fiji.sc), where only minor adjustment of the brightness and contrast were performed.

### Small Animal PET/MR Imaging

PET studies of the gastrointestinal tract were performed in male C57/BL6J mice in two experimental series. The first experimental series (age = 14 months; weight = 28–35 g), was composed of a control group (*n* = 5) and a treated group (*n* = 6), respectively unexposed or exposed for 2 months to rotenone. The second experimental series (age = 16 months; weight = 28–35 g), was composed of a control group (*n* = 2) and a treated group (*n* = 5), respectively unexposed or exposed for 4 months to rotenone. To evaluate the impact of exposure to rotenone on the cholinergic innervation of the gut, we performed *in vivo* PET imaging. We selected a clinically used radiotracer, (–)-[^18^F]Flubatine, selective of the $\alpha_{4}\beta_{2}^{*}$ nicotinic acetylcholine receptor subtype to perform these experiments. The reasons to select this radiotracer were its rapid availability and its pharmacokinetic characteristics. (–)-[^18^F]Flubatine is not metabolized through the intestine and this is an important property, as it would otherwise dramatically increase the signal in this region interfering with the analysis. The animals received an intravenous injection of (–)-[^18^F]Flubatine into the tail vein (6.4 ± 1.9 MBq; 0.63 ± 0.58 nmol/kg; A_m_: 1,016 ± 712 GBq/μmol (EOS) for the 2 months treated serie) or retro-orbital (6.5 ± 2.4 MBq; 0.18 ± 0.18 nmol/kg; A_m_: 4,460 ± 2,115 GBq/μmol (EOS); for the 4 months treated serie) followed by a 60-min PET/MR scan (Mediso nanoScan®, Budapest, Hungary). A T1-weighted WB gradient echo sequence (GRE, repetition time = 20 ms, echo time = 6.4 ms) was performed for AC and anatomical orientation. The reconstruction parameters for the list mode data were the following: 3D-ordered subset expectation maximization (OSEM), four iterations, six subsets. The mice were positioned prone in a special mouse bed (heated up to 37°C), with the head fixed to a mouth piece for the anesthetic gas supply with isoflurane in 40% air and 60% oxygen (anesthesia unit: U-410, Agnthos, Lidingö, Sweden; gas blender: MCQ, Rome, Italy), image co-registration and evaluation of the volume of interest (VOI) were performed with PMOD (PMOD Technologies LLC, v. 3.9, Zurich, Switzerland). Spherical VOI with diameters of 1 to 2 mm were placed at the center of the caecum on the PET/MR fusion image. For the jejunum the VOI was delineated from the PET signal. The activity concentrations in the VOIs are expressed as the mean standardized uptake values (SUV) +/− SEM.

### Statistical Analysis

Concentration-response curves were compared using the 2-way ANOVA test with a Bonferroni's *post-hoc* test. Contractility data regarding frequency and EFS were analyzed using an unpaired Student *t*-test. Protein expression data was analyzed using an unpaired non-parametric Student *t*-test (Mann-Whitney Test). Significance was set at *p* < 0.05. The time-activity curves were analyzed using an unpaired Student *t*-test. Significance was set at *p* < 0.05.

## Results

### Rotenone Exposure Induces Changes in Gastrointestinal Contractility in Response to Carbachol and Dopamine

The effect of rotenone on the cholinergic and dopaminergic inputs to the ENS was determined by comparing the effects carbachol and dopamine on motility of intestinal tissue from vehicle and rotenone-exposed mice. Our results show that rotenone induces region- and exposure time-dependent changes in the concentration-response curves for carbachol and dopamine. We did not observe any effects on the basal frequency or mean basal contraction amplitude (i.e., after equilibration the samples and before adding any drugs) between intestinal segments from vehicle- (control) and rotenone-exposed mice (Supplementary Figure 1).

#### Changes in the Response to Carbachol After 2 and 4 Months of Rotenone Exposure

**Figure 1:**
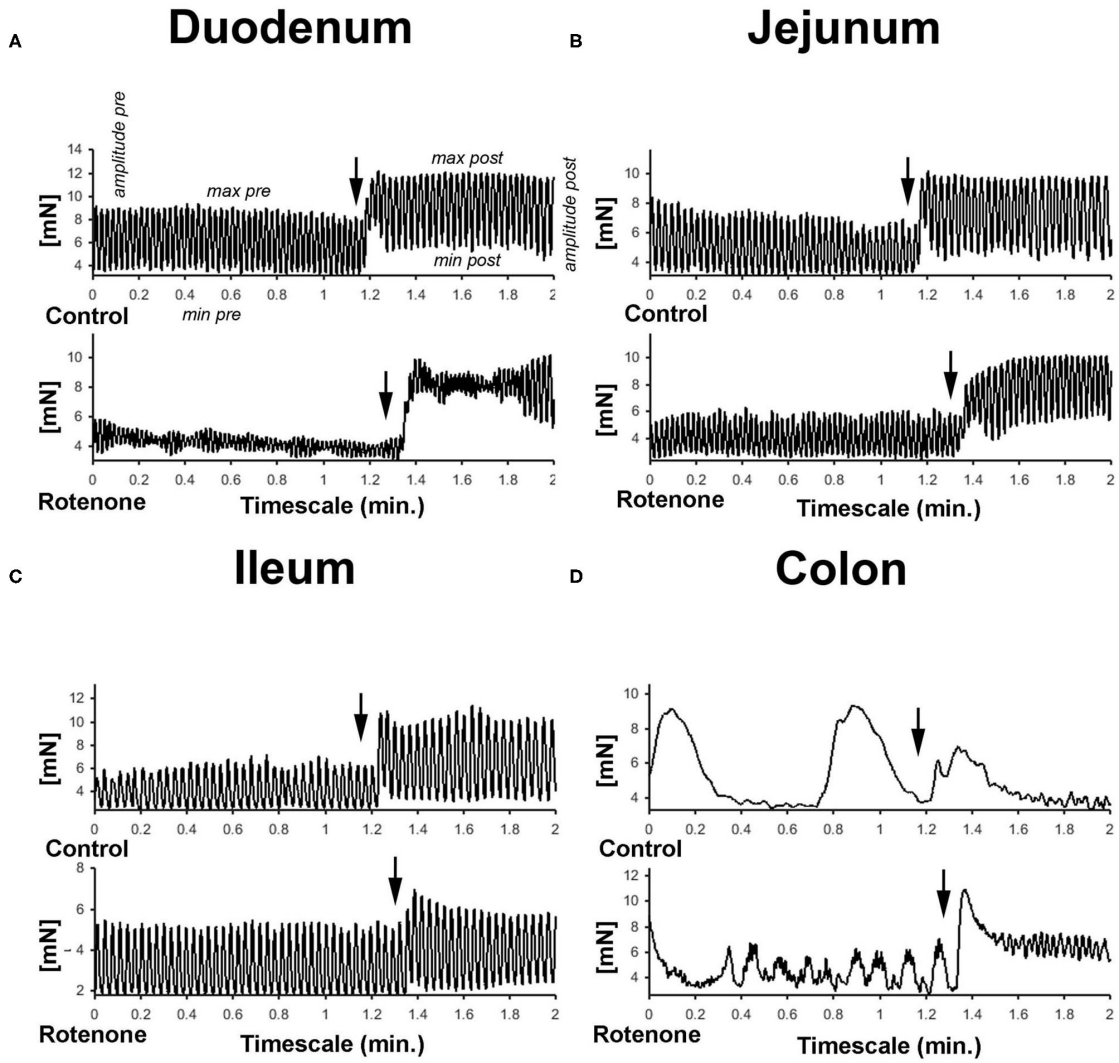
**(A–D)** Graphic showing representative examples of the force generated by the peristaltic movements and the effect of carbachol in the different gut segments in mN. Arrows in all graphics indicates the moments carbachol was added to the buffer.

Carbachol is a cholinergic receptor agonist that enhances intestinal contractility. We constructed concentration-response curves for carbachol in all intestinal segments from control and rotenone-exposed mice (2 and 4 months of exposure) (see Figure 1).

**Figure 2:**
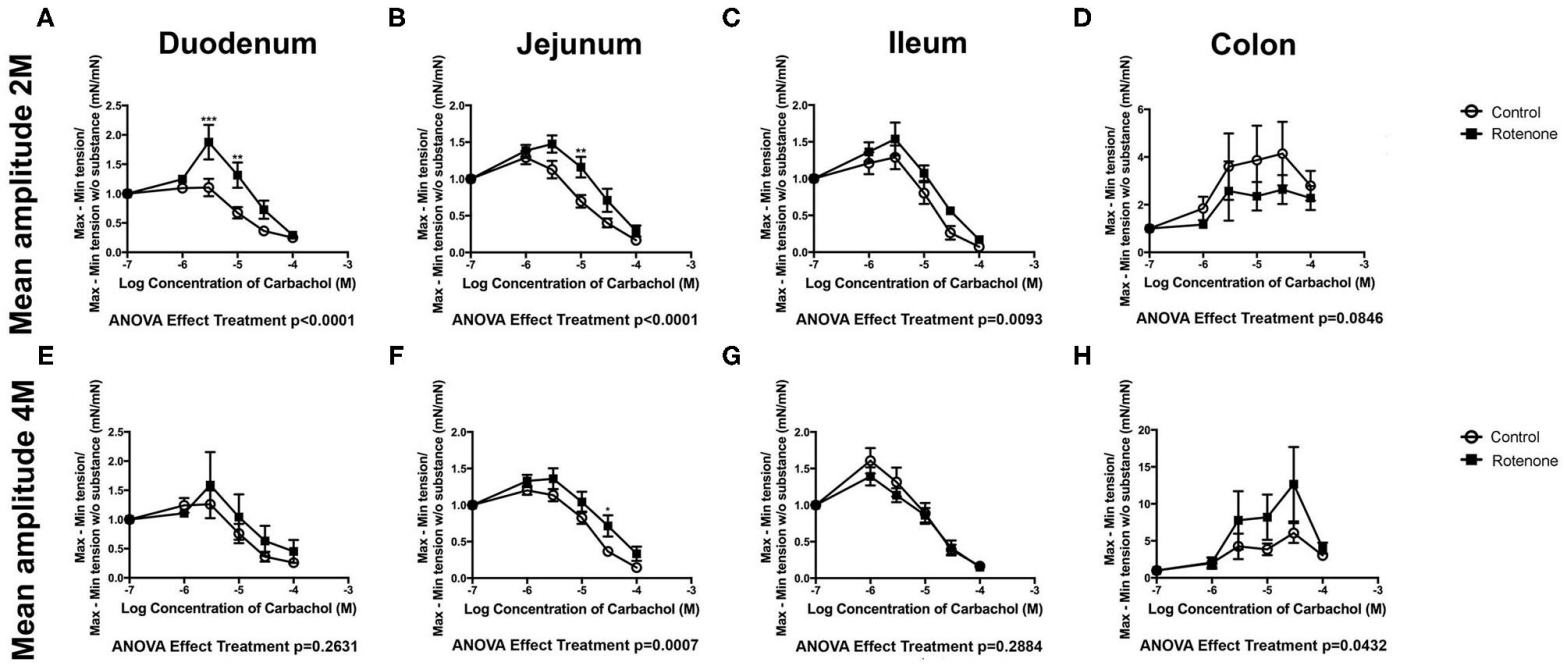
Two- and 4-Month rotenone exposure in mice increases the sensibility to carbachol in duodenum and jejunum. Effect of carbachol treatment on the amplitude (mean amplitude) of the duodenum **(A,E)**, jejunum **(B,F)**, ileum **(C,G)**, and colon **(D,H)** from control or 2 or 4 months rotenone-exposed mice. Data was analyzed using a 2-way ANOVA and a Bonferroni *post-hoc* test. Values of the effect of exposure are shown under the corresponding graph. *, **, *** correspond to *P* < 0.05, *P* < 0.01, and *P* < 0.001, respectively for each concentration obtained with the Bonferroni *post-hoc* test. Error bars represent SEM in all graphics.

In comparison with intestinal tissue from unexposed mice, 2 months of rotenone exposure led to larger increases in the maximum force in response to carbachol in duodenum and jejunum (see Supplementary Figures 2A,E), to elevation of carbachol-stimulated tone in duodenum (see Supplementary Figure 2B) and higher contraction amplitudes in the duodenum, jejunum and ileum (see Figures 2A–G). In comparison to vehicle exposure, 4 months of rotenone exposure produced a slightly different pattern of changes: carbachol increased maximum contractility to a larger extent in the jejunum and to a lower extent in the ileum (see Supplementary Figures 2G,K), increased tone in the jejunum (see Supplementary Figure 2H) to a larger extent, and induced larger contraction amplitudes in the jejunum and the colon (see Figures 2F,H and Table 1).

**Table 1:** Statistical results comparing the reaction to carbachol between vehicle and rotenone-exposed mice.

**Carbachol**		**2-way ANOVA treatment** ***p*****-value**		
		***p* mean amplitude**	***p* maximum**	***p* minimum**
Duodenum	2M	**<0.001**	**<0.001**	**0.003**
	4M	0.263	0.473	0.930
Jejunum	2M	**<0.001**	**0.038**	0.517
	4M	**<0.001**	**<0.001**	**<0.001**
Ileum	2M	0.009	0.660	0.901
	4M	0.288	**0.006**	0.061
Colon	2M	0.085	0.606	0.751
	4M	**0.043**	0.264	0.102

*A 2-way ANOVA was used to compare the effect of carbachol on the amplitude, the maximal exerted contraction force, and the muscle tone between vehicle and rotenone-exposed mice. The level of significance is shown as the p-value. Bold values are the significant values*.

#### Changes in Response to Dopamine After 2 and 4 Months of Rotenone Exposure

**Figure 3:**
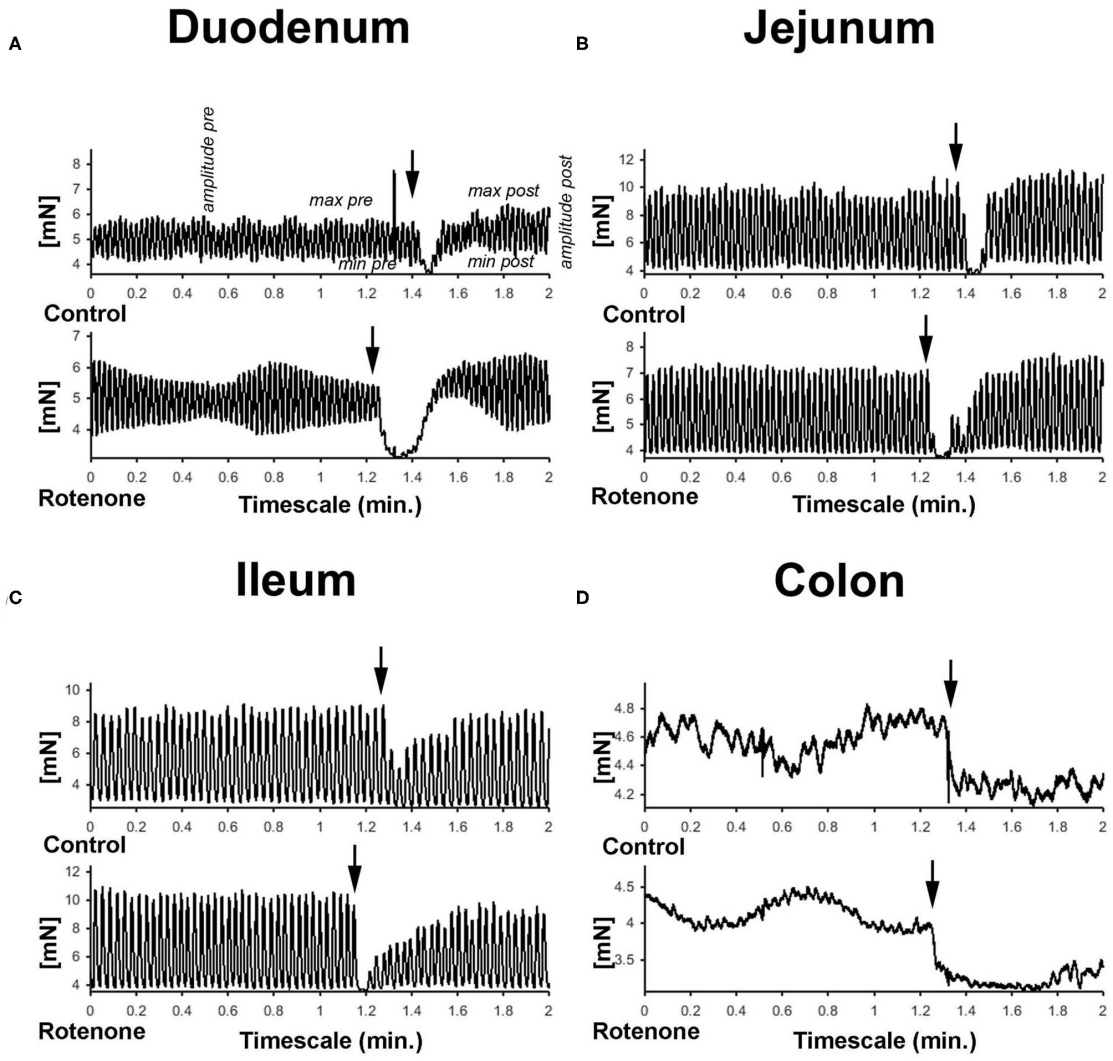
**(A–D)** Graphic showing representative examples of the force generated by the peristaltic movements and the effect of dopamine in the different gut segments in mN.

We then analyzed the effect of rotenone on the responses to dopamine among the different regions of the intestine. The neurotransmitter dopamine induces intestinal relaxation by decreasing tone, maximum force development and amplitude of contraction (see Figure 3).

**Figure 4:**
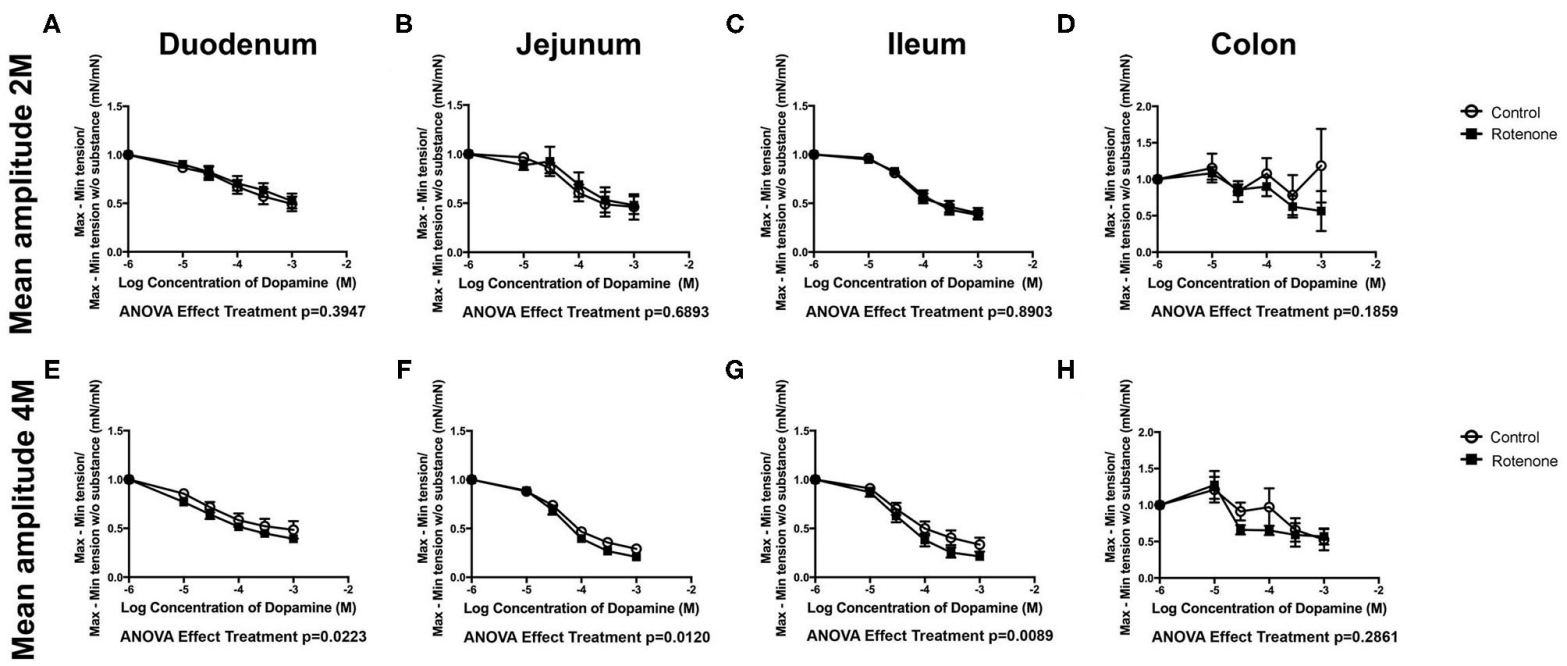
Two- and 4-Month rotenone exposure in mice increases the sensibility to dopamine in duodenum and jejunum. Effect of dopamine treatment on the amplitude (Mean amplitude) of the duodenum **(A,E)**, jejunum **(B,F)**, ileum **(C,G)**, and colon **(D,H)** from control or 2 or 4 months rotenone-exposed mice. Data was analyzed using a 2-way ANOVA and a Bonferroni *post-hoc* test. Values of the effect of exposure are shown under the corresponding graph. *, **, *** correspond to *P* < 0.05, *P* < 0.01, and *P* < 0.001, respectively for each concentration obtained with the Bonferroni *post-hoc* test. Error bars represent SEM in all graphics.

Two months of rotenone exposure led to a bigger dopamine-induced decrease in tone of duodenum, jejunum and ileum than in control preparations (see Supplementary Figures 3B,F,J). We did not observe any significant difference in the effect of dopamine on the maximum force of contraction or on contraction amplitude after 2 months of exposure with rotenone in comparison with vehicle exposure. However, after 4 months of exposure to rotenone, dopamine induced bigger decreases in the maximum force of contraction in jejunum and ileum (see Supplementary Figures 3G,K), larger relaxation of tone in duodenum and ileum (see Supplementary Figures 3D,L) and greater depression of mean contraction amplitude in duodenum, jejunum, and ileum (see Figures 4A-H). Interestingly, the effects in colon were the opposite direction with smaller increase in the maximum force of contraction and a weaker relaxation in tone of rotenone-exposed samples (see Supplementary Figures 3O,P and Table 2 for all results).

**Table 2:** Statistical results comparing the reaction to dopamine between vehicle and rotenone-exposed mice.

**Dopamine**		**2-way ANOVA treatment** ***p*****-value**		
		***p* mean amplitude**	***p* maximum**	***p* minimum**
Duodenum	2M	0.395	0.516	**0.027**
	4M	**0.022**	0.364	**0.012**
Jejunum	2M	0.689	0.891	**<0.001**
	4M	**0.012**	**<0.001**	0.800
Ileum	2M	0.890	0.814	**<0.001**
	4M	**0.009**	**0.001**	**0.011**
Colon	2M	0.186	0.477	0.229
	4M	0.286	**0.006**	**0.003**

*A 2-way ANOVA was used to compare the effect of dopamine on the amplitude, the maximal exerted contraction force, and the muscle tone between vehicle and rotenone-exposed mice. The level of significance is shown as the p-value. Bold values are the significant values*.

Taken together, these results show that in general in mice exposed to rotenone carbachol and dopamine have increased effects when compared to the control group, suggesting an over-expression of the postsynaptic receptors in ENS neurons due to a decreased parasympathetic and dopaminergic input. From a physiological point of view these effects are expected to be quite variable and even divergent between intestinal regions and the duration of the exposure. However, the signal to noise background ratio is still above the threshold. Such a reduction in the cholinergic input would be in accordance with previous observation from our lab and could be responsible for decreased intestinal motility previously observed using the 1-h stool collection test (13).

### Rotenone Exposure Induces Changes in the Response of the ENS to Electric Field Stimulation

**Figure 5:**
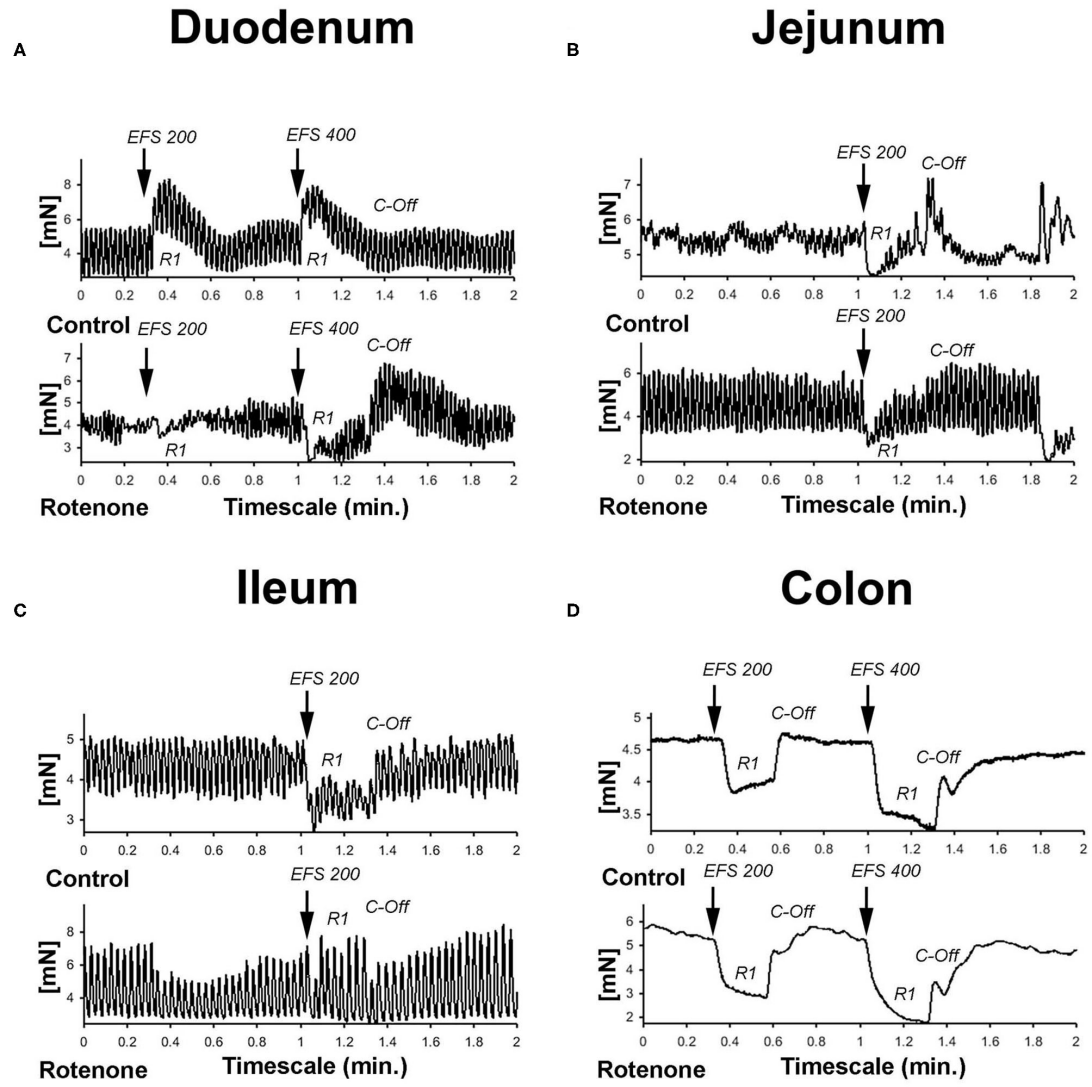
**(A–D)** Graphic showing representative examples of the force generated by the peristaltic movements and the effect of EFS in the different gut segments in mN. Electric field stimulation normally induces an initial relaxation (R1) followed by a contraction (C-Off) when the stimulus ended.

We then tested the effect of rotenone exposure on the response of the ENS to electric field stimulation (EFS). EFS allows to induce indirect contractile effects on the intestine due to excitation of ENS and subsequent neurotransmitter release without directly electrically stimulating the intestinal smooth muscle. The response to EFS depends on the distribution of the neuronal subtypes within the different various regions of the intestine (i.e., cholinergic, serotoninergic, dopaminergic, nitrergic, etc. neurons). Using the parameters chosen for the experiments (see Material and Methods) EFS with a pulse duration of 200 (EFS 200) and 400 (EFS 400) μs (and using the parameters described in Material and Methods) produced a biphasic tetrodotoxin-sensitive response in control tissue from non-exposed mice as reported previously (17). This response consisted in an initial relaxation (R1) after the start of EFS followed by a contraction (C-Off) when the EFS stopped (see Figure 5).

#### Four Months but Not 2 Months Rotenone Exposure Increases the Response of Jejunum and Ileum to EFS in the Absence and Presence of Atropine, Guanethidine, or Serotonin

In order to compare the responses to EFS in control and rotenone-exposed mice, we performed EFS 200 and EFS 400 in the absence or in the presence of atropine (AT), AT and guanethidine (G) (2 months) or AT, G and serotonin (S) (4 months). We then compared both curves using a 2-way ANOVA. We could not detect any significant differences during EFS 200 or EFS 400 stimulation between control and 2 months rotenone-exposure (see Supplementary Figures 4A–H, 5A–H). After 4 months of rotenone exposure intestinal muscle relaxations were enhanced during EFS 200 stimulation (R1 phase) in the ileum (see Supplementary Figure 4M) and so were reactive contractions when stimulation stopped (C-Off phase) in the jejunum and ileum (see Supplementary Figures 4L,N). Similar differences between exposures were observed in these regions when an EFS 400 was applied (see Supplementary Figures, 5L–N and Tables 3, 4 for a summary).

**Table 3:** Shows the mean values (mean), the standard error of the mean (SEM), and the significance (*p*) of the comparison (unpaired Student's *t*-test) between the relaxation observed in R1 induced by the EFS (200 and 400 ms) in the absence of any blocker (consider as 100%) and the relaxation obtained in the presence of atropine (AT) normalized to the value without substance and shown as percentage.

**R1 AT**		**Control**		***p***	**Rotenone**		***p***	***p* control vs. rotenone**
		**Mean**	**SEM**		**Mean**	**SEM**		
Duodenum	2M EFS 200	315.191	175.939	0.249	116.545	85.781	0.851	0.333
	2M EFS 400	41.398	20.512	**0.017**	44.004	18.337	**0.012**	0.926
	4M EFS 200	146.393	45.084	0.290	153.464	41.517	0.216	0.148
	4M EFS 400	168.833	69.876	0.311	215.126	92.574	0.232	0.342
Jejunum	2M EFS 200	118.449	33.240	0.591	72.171	42.528	0.528	0.397
	2M EFS 400	155.139	67.374	0.432	66.621	31.924	0.349	0.272
	4M EFS 200	201.091	49.656	0.059	81.788	18.877	0.349	**0.018**
	4M EFS 400	134.984	42.752	0.425	83.759	13.506	0.247	0.499
Ileum	2M EFS 200	341.479	280.242	0.409	122.493	46.901	0.642	0.459
	2M EFS 400	30.042	21.168	**0.008**	136.076	36.440	0.346	**0.031**
	4M EFS 200	257.809	102.348	0.121	185.114	57.208	0.156	0.116
	4M EFS 400	59.825	6.443	**0.033**	155.883	31.187	0.092	0.908
Colon	2M EFS 200	80.848	15.441	0.243	230.193	98.750	0.217	0.166
	2M EFS 400	59.825	6.443	0.283	122.885	16.826	0.204	0.107
	4M EFS 200	63.827	14.303	**0.022**	70.743	10.085	**0.010**	0.698
	4M EFS 400	63.568	14.458	**0.022**	70.603	9.899	**0.009**	0.693

*The far-right column shows the significance when comparing the results obtained in the presence of AT between control and rotenone exposed mice. Bold values are the significant values*.

**Table 4:** Shows the mean values (mean), the standard error of the mean (SEM), and the significance (*p*) of the comparison (unpaired Student's *t*-test) between the contraction observed in C-Off induced by the EFS (200 and 400 ms) in the absence of any blocker (consider as 100%) and the contraction obtained in the presence of atropine (AT).

**C-Off AT**		**Control**		***p***	**Rotenone**		***P***	***p* control vs. rotenone**
		**Mean**	**SEM**		**Mean**	**SEM**		
Duodenum	2M EFS 200	79.173	14.143	0.171	73.393	11.249	**0.040**	0.756
	2M EFS 400	95.322	20.113	0.821	90.426	18.887	0.623	0.863
	4M EFS 200	81.353	9.268	**0.049**	65.884	6.538	**<0.001**	0.185
	4M EFS 400	88.081	8.791	0.169	68.931	6.566	**<0.001**	0.097
Jejunum	2M EFS 200	95.973	15.359	0.799	78.510	32.023	0.517	0.634
	2M EFS 400	98.213	15.010	0.908	75.032	14.189	0.109	0.288
	4M EFS 200	68.503	10.650	**0.009**	79.165	10.480	0.064	0.486
	4M EFS 400	74.035	7.839	**0.004**	82.448	10.150	0.103	0.521
Ileum	2M EFS 200	73.371	25.338	0.318	65.949	12.992	**0.026**	0.8
	2M EFS 400	80.838	20.061	0.362	76.370	10.713	0.052	0.848
	4M EFS 200	81.073	21.466	0.363	59.825	6.443	**<0.001**	0.334
	4M EFS 400	76.077	14.726	0.104	63.085	6.077	**<0.001**	0.408
Colon	2M EFS 200	99.826	36.553	0.996	27.022	11.878	**<0.001**	0.087
	2M EFS 400	94.823	47.659	0.916	−16.350	27.606	**0.002**	0.08
	4M EFS 200	109.166	16.862	0.594	97.017	13.881	0.833	0.586
	4M EFS 400	104.363	11.605	0.712	97.498	16.565	0.882	0.739

*The far-right column shows the significance when comparing the results obtained in the presence of AT between control and rotenone exposed mice. Bold values are the significant values*.

Altogether, these results show that rotenone exposure leads to a stronger net inhibitory outcome of the enteric neuronal network thus suggesting either a stronger inhibitory or a weaker excitatory neuronal input in the ENS of rotenone-exposed mice. This data correlate well with the previously reported reduction in pellet output after 1.5 months of such a regimen (i.e., chronic low dose rotenone exposure) (13). In order to differentiate between these two options, we compared the effect of EFS alone and in the presence of (i) atropine (AT), a competitive muscarinic acetylcholine (Ach) receptor antagonist to investigate non-cholinergic responses and (ii) AT and guanethidine (G), a selective inhibitor of noradrenaline release from postganglionic adrenergic neurons to investigate non-adrenergic non-cholinergic responses. Additionally, we decided to analyze (iii) AT, G, and serotonin (S), a neurotransmitter that increases gut motility and the peristaltic reflex by inhibiting dopaminergic neurons among other effects, to investigate the basal levels of serotonin in the ENS.

### Rotenone Exposure Reduces the Amount of Acetylcholine Released in Duodenum, Ileum, and Colon and Increases It in the Jejunum During EFS

To indirectly determine the amount of ACh released during the EFS, we compared the relaxation and contraction during the R1 and C-Off phases respectively in the absence and the presence of 10^−5^ Mol/L of AT.

**Figure 6:**
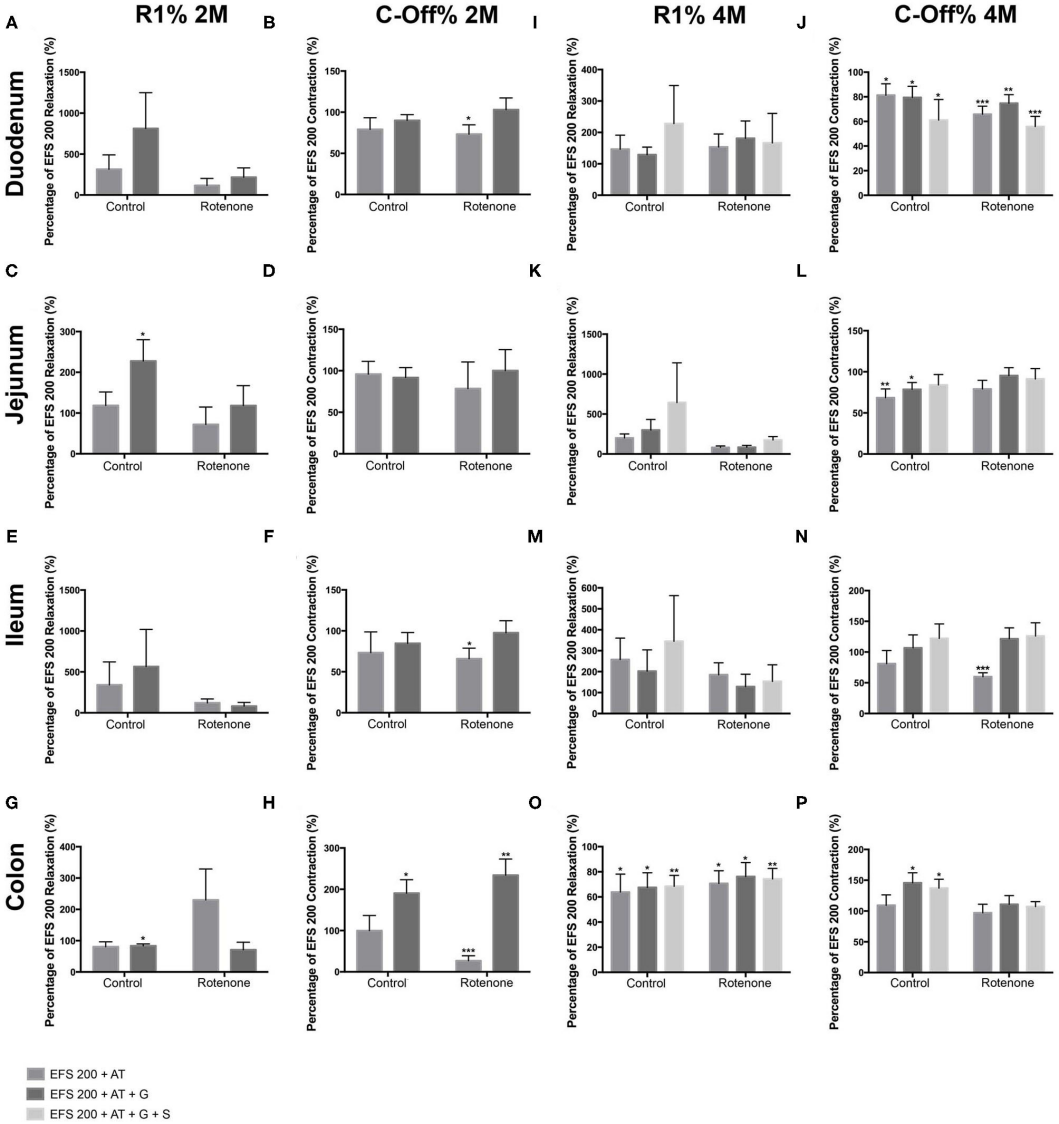
Effect of a 200 μs electric field stimulation (EFS 200) on the different intestinal regions after 2 and 4 months of rotenone exposure in mice. In all regions, AT induced a deeper relaxation and a lower contraction when compared to the reaction observed without atropine (AT) or guanethidine (G) when EFS 200 and EFS 400 were applied. Data in figures is shown as a percentage of the EFS 200 or EFS 400 relaxation/contraction obtained without neurotransmitters and the *p*-values refer to this percentage, if an additional significant difference between control and exposed samples is shown, the *p*-value will be written separately. **(A–H)** Graphics showing the R1 and the C-Off induced by EFS 200 on the duodenum **(A,B)**, jejunum **(C,D)**, ileum **(E,F)**, and colon **(G,H)** in Krebs solution alone, in the presence of atropine or in the presence of atropine and guanethidine after 2 months of rotenone exposure. **(I–P)** Graphics showing the R1 and the C-Off induced by EFS 200 on the duodenum **(I,J)**, jejunum **(K,L)**, ileum **(M,N)**, and colon **(O,P)** in Krebs solution alone, in the presence of atropine or in the presence of atropine and guanethidine after 4 months of rotenone exposure. Data was analyzed using a 2-way ANOVA Values of the effect of exposure are shown under the corresponding graph. *, **, *** correspond to *P* < 0.05, *P* < 0.01, and *P* < 0.001, respectively. Error bars represent SEM in all graphics. Data was analyzed using a Student's *t*-test, *, **, *** correspond to *P* < 0.05, *P* < 0.01, and *P* < 0.001, respectively. Error bars represent SEM in all graphics.

**Figure 7:**
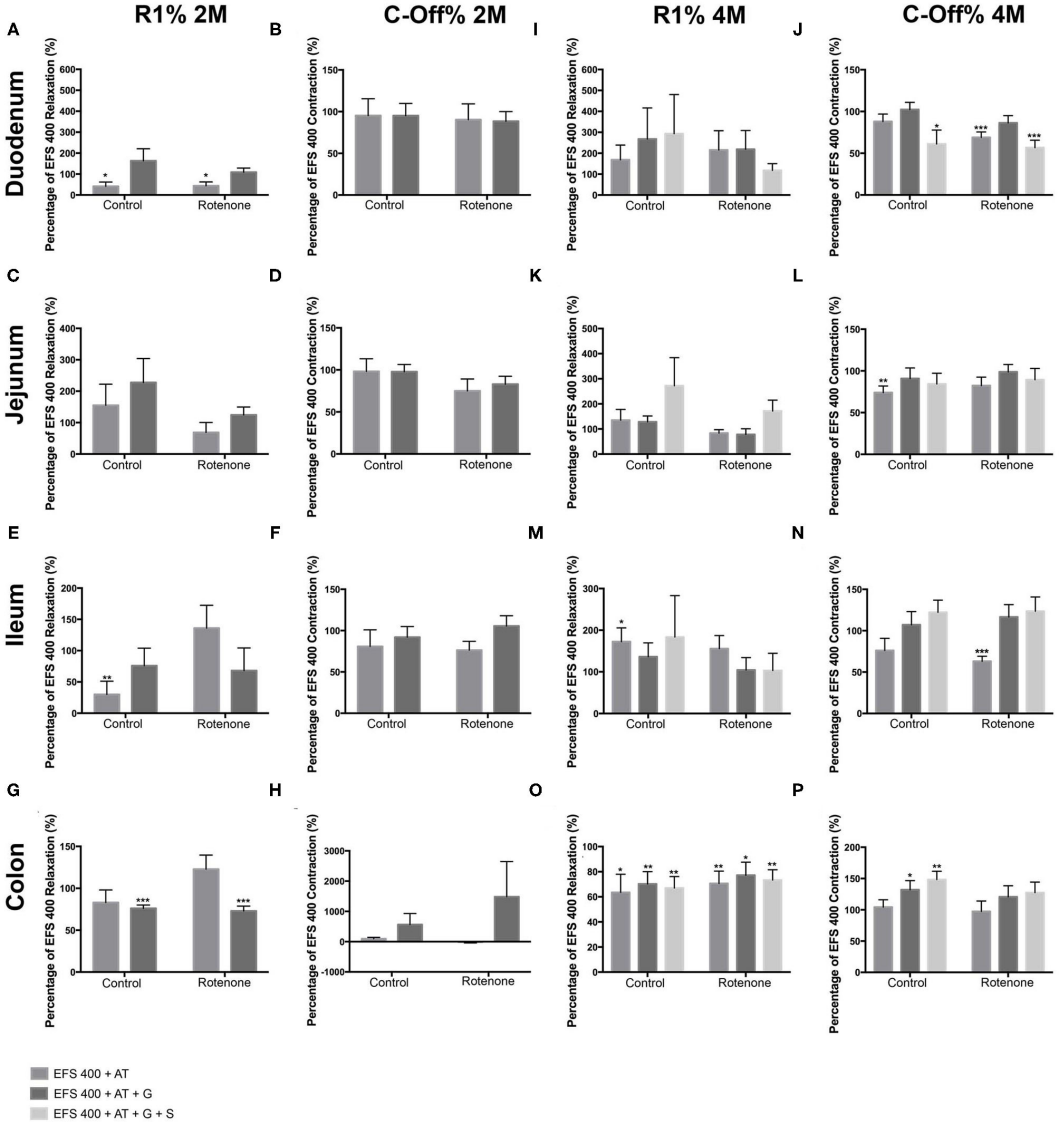
Effect of a 400 μs electric field stimulation (EFS 400) on the different intestinal regions after 2 and 4 months of exposure. In all regions, AT induced a deeper relaxation and a lower contraction when compared to the reaction observed without atropine (AT) or guanethidine (G) when EFS 200 and EFS 400 were applied. Data in figures is shown as a percentage of the EFS 200 or EFS 400 relaxation/contraction obtained without substances and the *p*-values refer to this percentage, if an additional significant difference between control and exposed samples is shown, the *p*-value will be written separately. **(A–H)** Graphics showing the R1 and the C-Off induced by EFS 200 on the duodenum **(A,B)**, jejunum **(C,D)**, ileum **(E,F)**, and colon **(G,H)** in Krebs solution alone, in the presence of atropine or in the presence of atropine and guanethidine after 2 months of rotenone exposure. **(I–P)** Graphics showing the R1 and the C-Off induced by EFS 200 on the duodenum **(I,J)**, jejunum **(K,L)**, ileum **(M,N)**, and colon **(O,P)** in Krebs solution alone, in the presence of atropine or in the presence of atropine and guanethidine after 4 months of rotenone exposure. Data was analyzed using a 2-way ANOVA Values of the effect of exposure are shown under the corresponding graph. *, **, *** correspond to *P* < 0.05, *P* < 0.01, and *P* < 0.001, respectively. Error bars represent SEM in all graphics. Data was analyzed using a Student's *t*-test, *, **, *** correspond to *P* < 0.05, *P* < 0.01, and *P* < 0.001, respectively. Error bars represent SEM in all graphics.

In the duodenum, we did not observe differences in the effect of AT on the R1-relaxation between controls and exposed mice after 2 months of rotenone exposure (see Figures 6A, 7A). The inhibitory effect of AT on the C-Off contraction induced by EFS 200 and EFS 400 was slightly stronger on exposed mice when compared to control mice (see Figures 6B, 7B). Four months of rotenone exposure did not change the effect of AT on the R1-relaxation between controls and exposed mice (see Figures 6I, 7I), but the effect of AT on the C-Off contraction induced by EFS 200 and EFS 400 on exposed mice was stronger than on control mice (see Figures 6J, 7J and Tables 3, 4 for a data summary).

In the jejunum, we observed no differences in the effect of AT on the R1 or C-Off phases of EFS 200 or EFS 400 after 2 months of exposure (see Figures 6C,D, 7C,D). Four months after exposure, the effect of AT on R1-relaxation induced by EFS 200 and EFS 400 was significantly different between control and exposed samples (*p* < 0.05). AT induced larger R1-relaxation on control samples and reduced the R1-relaxation in exposed samples (see Figures 6K, 7K). The differences observed on the effect of AT on the C-Off phase between rotenone exposed and control samples was opposite to the one observed in the duodenum, the ileum and the colon. AT induced a significant decrease in the contractility of control mice, whereas the decrease observed on exposed mice was smaller and non-significant (see Figures 6L, 7L and Tables 3, 4).

In the ileum, 2 months exposure induced a higher response to AT (*p* < 0.05) in the R1 phase with higher relaxations during EFS 400 in rotenone-exposed mice when compared to control samples (see Figures 6E, 7E). We also observed a significant reduction of the C-Off contraction in the presence of AT with the EFS 200 on exposed samples but not in controls (see Figures 6F, 7F). Four months of rotenone exposure induced a slightly higher R1-relaxation in the presence of AT on control mice than in exposed mice (see Figures 6M, 7M). The effect of AT on the C-Off contractility was higher on exposed samples than controls (see Figures 6N, 7N and Tables 3, 4).

In the colon EFS 200 and EFS 400 induced an opposite reaction to the one observed in all other intestinal segments in rotenone-exposed samples with a contraction in the R1 phase instead of a relaxation (see Figures 6G,O, 7G,O). The contraction during the R1 reaction was opposite and significantly different (*p* < 0.05) to the relaxation observed in control samples during the EFS 200 (see Figure 6G). Additionally, we also observed an increased effect of AT on the C-Off phase during the EFS 200 with a significant reduction of the C-Off contraction in the presence of AT on exposed samples but not in controls (see Figure 6H). After 4 months of exposure, AT had no effect on the C-Off contractility in control or exposed mice (see Figures 6P, 7P). Interestingly, the effect of AT on the R1 EFS-induced relaxation after 4 months was opposite to all other intestinal regions and to the effect after 2 months of exposure. AT diminished the relaxation induced by EFS in both control and exposed samples (see Figures 6O, 7O). No difference between exposures was observed (see Tables 3, 4).

Overall the increased relaxation in the R1 phase and reduced percentage of contraction in the C-off phase suggest that also the amount of ChAT intrinsic to the enteric nervous system is also reduced in rotenone exposed mice when compared to control mice.

### Two Months but Not 4 Months Rotenone Exposure Influences the Noradrenergic, Non-cholinergic (NANC) Reaction to EFS 200 or EFS 400

We then analyzed the NANC reaction of the different regions to the EFS 200 and EFS 400. For this we added 10^−5^ M guanethidine to the Krebs solution, which already contained AT. As expected when inhibiting the noradrenergic input to the ENS, the NANC reaction to EFS showed non-significant increases in the R1 relaxation and non-significant higher C-Off contractions in most of the regions when compared to the reaction with AT at any time point in control mice. After 2 months of rotenone exposure, the only region showing a significant difference between the reaction to AT alone and to AT+G was the colon. In exposed samples but not in controls, the addition of guanethidine induced an increased contraction in the C-Off phase during EFS 200 and a reduced relaxation in the R1 phase during EFS (see Figures 7G,H). After 4 months of exposure, we only observed a significant increase in the C-Off contraction after addition of guanethidine during EFS 200 and EFS 400 in the ileum of rotenone-exposed mice, the same region where the effect of AT had been the highest (see Figures 6P, 7P). Our results showed no significant differences in the NANC reaction to EFS 200 and EFS 400 between rotenone exposed and control samples in all other regions studied (see Tables 5, 6).

**Table 5:** Shows the mean values (mean), the standard error of the mean (SEM), and the significance (*p*) of the comparison (unpaired Student's *t*-test) between the relaxation (R1) obtained with the EFS (200 and 400 ms) in the presence of atropine (AT) and in the presence of AT and guanethidine (G).

**R1 AT + G**		**Control AT**		**Control AT + G**		***p***	**Rotenone AT**		**Rotenone AT + G**		***p***	***p* control vs. rotenone**
		**Mean**	**SEM**	**Mean**	**SEM**		**Mean**	**SEM**	**Mean**	**SEM**		
Duodenum	2M EFS 200	315.191	175.939	813.131	426.648	0.315	116.545	85.781	217.024	115.050	0.500	0.216
	2M EFS 400	41.398	20.512	162.965	57.991	0.076	44.004	18.337	108.959	19.453	**0.035**	0.398
	4M EFS 200	146.393	45.084	129.178	24.202	0.742	153.464	41.517	181.382	55.165	0.691	0.169
	4M EFS 400	168.833	69.876	267.884	148.191	0.555	215.126	92.574	218.659	89.956	0.979	0.25
Jejunum	2M EFS 200	118.449	33.240	227.785	52.248	0.108	72.171	42.528	118.189	49.128	0.495	0.157
	2M EFS 400	155.139	67.374	227.716	76.153	0.492	68.621	31.924	124.575	24.811	0.196	0.227
	4M EFS 200	201.091	49.656	300.511	132.088	0.491	81.788	18.877	83.871	22.815	0.945	0.083
	4M EFS 400	134.984	42.752	128.653	23.275	0.898	83.759	13.506	78.240	22.770	0.838	0.746
Ileum	2M EFS 200	341.479	280.242	565.512	454.462	0.684	122.493	46.901	82.262	44.370	0.547	0.315
	2M EFS 400	30.042	21.168	76.008	27.997	0.219	136.076	36.440	68.049	36.293	0.215	0.866
	4M EFS 200	257.809	102.348	202.297	101.587	0.706	185.114	57.208	128.629	59.287	0.503	0.125
	4M EFS 400	172.554	33.006	136.134	33.290	0.450	155.883	31.187	104.455	29.719	0.250	0.61
Colon	2M EFS 200	80.848	15.441	83.618	6.007	0.871	230.193	98.750	70.959	24.153	0.148	0.622
	2M EFS 400	82.976	15.025	76.104	3.871	0.667	122.885	16.826	72.993	5.818	**0.019**	0.666
	4M EFS 200	63.827	14.303	67.565	11.668	0.842	70.743	10.085	76.206	11.101	0.720	0.594
	4M EFS 400	63.568	14.458	70.230	9.817	0.708	70.603	9.899	77.240	10.367	0.650	0.63

*The absence of any blocker is considered as 100% and all other values were normalized to that value. The far-right column shows the significance when comparing the results obtained in the presence of AT and G between control and rotenone exposed mice. Bold values are the significant values*.

**Table 6:** Shows the mean values (mean), the standard error of the mean (SEM), and the significance (*p*) of the comparison (unpaired Student's *t*-test) between the contraction (C-Off) obtained with the EFS (200 and 400 ms) in the presence of atropine (AT) and in the presence of AT and guanethidine (G).

**C-Off AT + G**		**Control AT**		**Control AT + G**		***p***	**Rotenone AT**		**Rotenone AT + G**		***P***	***p* control vs. rotenone**
		**Mean**	**SEM**	**Mean**	**SEM**		**Mean**	**SEM**	**Mean**	**SEM**		
Duodenum	2M EFS 200	79.173	14.143	90.052	7.056	0.507	73.393	11.249	103.054	14.259	0.133	0.433
	2M EFS 400	95.322	20.113	95.215	14.599	0.997	90.426	18.887	88.545	11.469	0.934	0.727
	4M EFS 200	81.353	9.268	79.459	9.085	0.886	65.884	6.538	74.802	6.934	0.363	0.686
	4M EFS 400	88.081	8.791	102.368	8.660	0.266	68.931	6.566	86.449	8.614	0.125	0.214
Jejunum	2M EFS 200	95.973	15.359	91.761	12.065	0.833	78.510	32.023	100.143	25.408	0.608	0.772
	2M EFS 400	98.213	15.010	97.778	8.622	0.981	75.032	14.189	82.999	9.473	0.651	0.275
	4M EFS 200	68.503	10.650	78.574	8.404	0.569	79.165	10.480	95.389	9.728	0.273	0.209
	4M EFS 400	74.035	7.839	90.882	16.642	0.274	82.448	10.150	98.868	8.893	0.241	0.612
Ileum	2M EFS 200	73.371	25.338	84.767	13.115	0.698	65.949	12.992	97.729	14.628	0.135	0.524
	2M EFS 400	80.838	20.061	91.997	12.890	0.650	76.370	10.713	105.610	12.336	0.104	0.463
	4M EFS 200	81.073	21.466	106.900	20.971	0.404	59.825	6.443	121.527	17.834	**0.004**	0.601
	4M EFS 400	76.077	14.726	107.241	15.902	0.172	63.085	6.077	116.604	24.860	**0.004**	0.673
Colon	2M EFS 200	99.826	36.553	190.669	32.531	0.093	27.022	11.878	234.174	39.160	**<0.001**	0.413
	2M EFS 400	94.823	47.659	564.570	365.080	0.231	−16.350	27.606	1484.669	1165.451	0.227	0.468
	4M EFS 200	109.166	16.862	145.785	16.408	0.139	97.017	13.881	110.867	14.259	0.496	0.128
	4M EFS 400	104.363	11.605	132.025	14.554	0.157	97.498	16.565	120.712	17.657	0.352	0.628

*The absence of any blocker is considered as 100% and all other values were normalized to that value. The far-right column shows the significance when comparing the results obtained in the presence of AT and G between control and rotenone exposed mice. Bold values are the significant values*.

### ENS Reaction to Serotonin Is Not Altered by Rotenone Exposure

**Figure 8:**
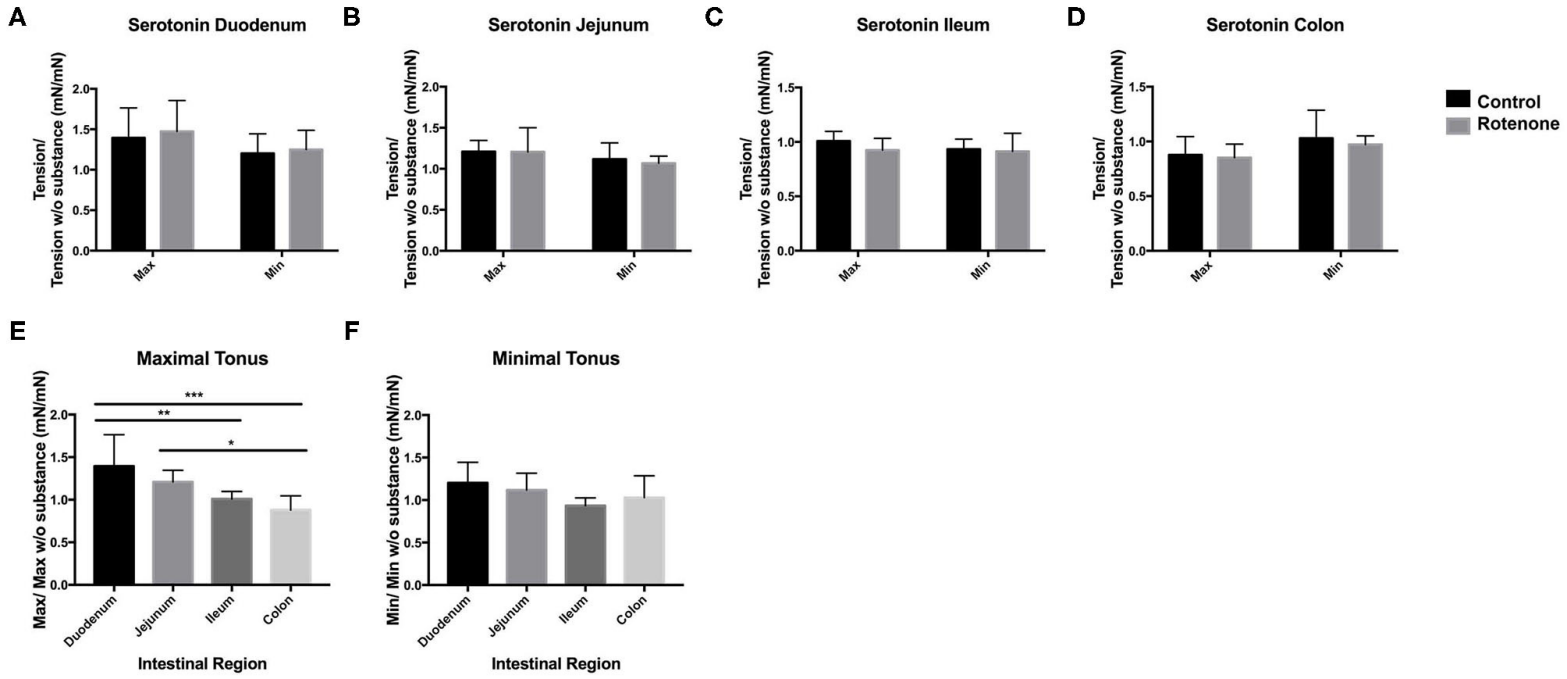
Effect of serotonin (S) on the maximal contractility force and the tone on all intestinal regions. **(A–D)** Bar graphs showing the effect of serotonin on the duodenum **(A)**, jejunum **(B)**, ileum **(C)**, and colon **(D)** of control (black) and rotenone-exposed (gray) mice. Bar graph in **(E)** shows a comparison of the maximal contractility force exerted by serotonin between intestinal regions in control mice. Bar graph in **(F)** shows a comparison of the effect of serotonin on the tone between intestinal regions in control mice. Data was analyzed using a Student's *t*-test. *, **, *** correspond to *P* < 0.05, *P* < 0.01, and *P* < 0.001, respectively. Error bars represent SEM in all graphics.

In PD, the serotoninergic system in the brainstem is affected during the course of the disease. Therefore, we tested whether rotenone exposure affected the serotoninergic system in the gut. For this we added 10^−4^ M of serotonin in the presence of AT and G. In all regions and exposure groups except the colon, serotonin addition induced an increased contractility with higher contractility forces. The magnitude of this increase was region dependent and no difference was observed between control and rotenone-exposed mice. In both groups the maximal contractility force was significantly higher (*p* < 0.05) in the duodenum when compared to the ileum and the colon and the maximal contractility force in the jejunum was significantly higher to the one exerted in the colon (*p* < 0.05) (see Figures 8A–F). The effect of serotonin on the intestinal tonus depended on the region. It induced an increase of tonus in the duodenum and the jejunum, it had no effect on the colon and it decreased the tonus in the ileum. We did no observe any differences between rotenone-exposed and control samples (*p* > 0.05) (see Figures 8A–F and Tables 7, 8).

**Table 7:** Shows the mean values (mean) and the standard error of the mean (SEM) for maximal tension exerted by serotonin (S) when added in the presence of AT and G.

**Serotonin max**	**Control**		**Rotenone**	
	**Mean**	**SEM**	**Mean**	**SEM**
Duodenum	1.395	0.13	1.474	0.127
Jejunum	1.209	0.046	1.205	0.099
Ileum	1.009	0.031	0.924	0.035
Colon	0.877	0.055	0.851	0.042
***p***	**Jejunum**	**Ileum**	**Colon**	
Duodenum	0.300	**0.006**	**<0.001**	
Jejunum		0.240	**0.013**	
Ileum			0.594

*The P-value table shows the significance of the difference in the effect that S had between regions of the intestine. Bold values are the significant values*.

**Table 8:** Shows the mean values (mean) and the standard error of the mean (SEM) for the muscle tone (Min.) by serotonin (S) when added in the presence of AT and G.

**Serotonin min**	**Control**		**Rotenone**	
	**Mean**	**SEM**	**Mean**	**SEM**
Duodenum	1.202	0.086	1.249	0.080
Jejunum	1.118	0.066	1.068	0.029
Ileum	0.933	0.033	0.914	0.055
Colon	1.030	0.085	0.972	0.026
***p***	**Jejunum**	**Ileum**	**Colon**	
Duodenum	0.840	**0.069**	0.342	
Jejunum		0.284	0.807	
Ileum			0.778	

*The P-value table shows the significance of the difference in the effect that S had between regions of the intestine. Bold values are the significant values*.

Altogether, our results on contractility show that, in general, intestine sections from rotenone-exposed mice have an increased sensibility to carbachol and dopamine. Such increases are normally due to an up-regulation in the expression of the corresponding receptors as a result of a reduced neurotransmitter release, either due to alterations in the synapse or to the degeneration of the producing neurons. EFS experiments also suggest that the amount of acetylcholine might be reduced in the ENS, which could be caused by a reduction in the number of cholinergic neurons. In two previous studies, it has been shown that the cholinergic and adrenergic innervation of the gut is significantly reduced after rotenone exposure in mice and guinea pig (13, 14). Therefore, we investigated the effect or rotenone exposure both on the expression of different postsynaptic receptors and on the enteric neuronal subpopulations.

### Cholinergic, Adrenergic, and Dopaminergic Postsynaptic Receptors Are Up Regulated in Rotenone-Exposed Mice When Compared to Controls

**Figure 9:**
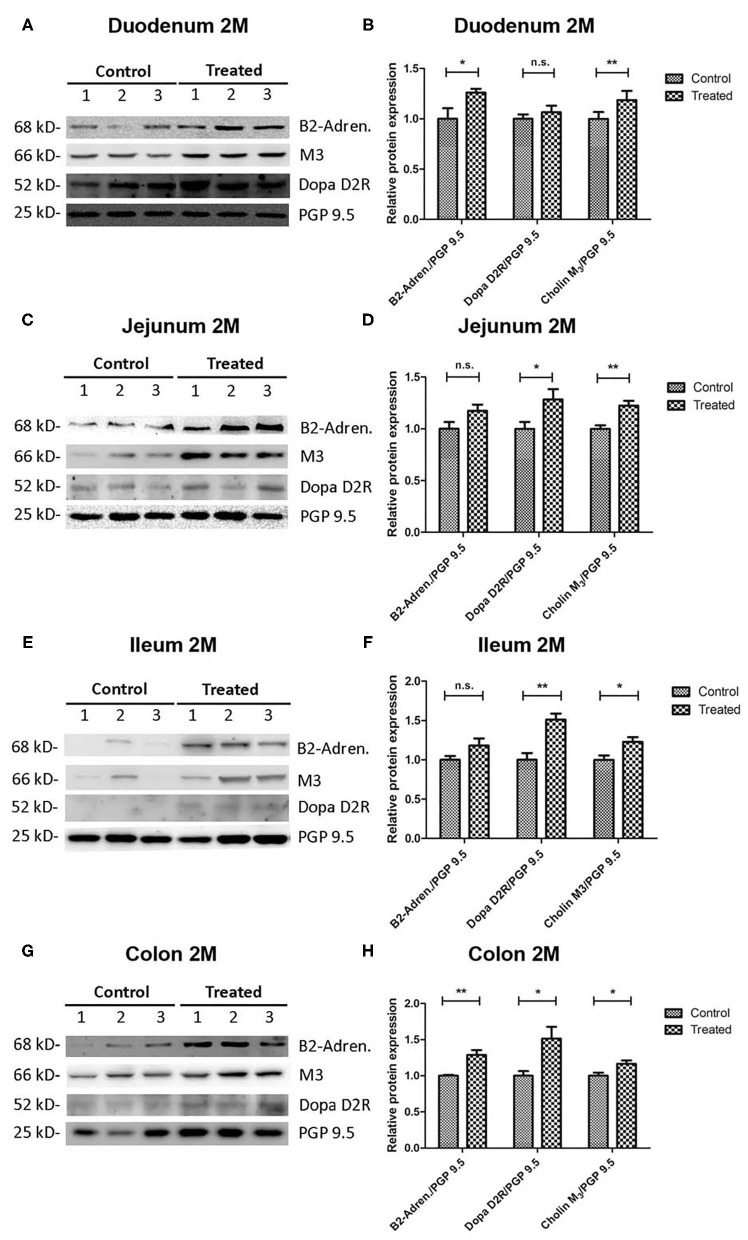
Two-Month rotenone exposure induces an up regulation of the adrenergic (ß2-subunit), cholinergic (M3-muscarinic), and dopaminergic (D2) receptors in the gut. Blots in **(A,C,E,G)** show 3 representative examples per exposure group for the protein levels of adrenergic (ß2-subunit) (B2-Adren.), cholinergic (M3-muscarinic) (M3), and dopaminergic (D2) (Dopa D2R) receptors in duodenum **(A)**, jejunum **(C)**, ileum **(E)**, and colon **(G)**. The protein gene product 9.5 (PGP 9.5) was used as a panneuronal marker to determine the neuronal population. Images were quantified using FIJI as described above. Protein expression for each marker was normalized to the amount of neuronal tissue in the sample by dividing it to the values obtained for PGP9.5 expression. Bar graphs in **(B,D,F,H)** show the quantification of the original blots (*n* = 5). Data was analyzed using a Student's *t*-test (Mann-Whitney Test). *, **, correspond to *P* < 0.05, and *P* < 0.01, respectively. n.s. non-significant. Error bars represent SEM in all graphics.

**Figure 10:**
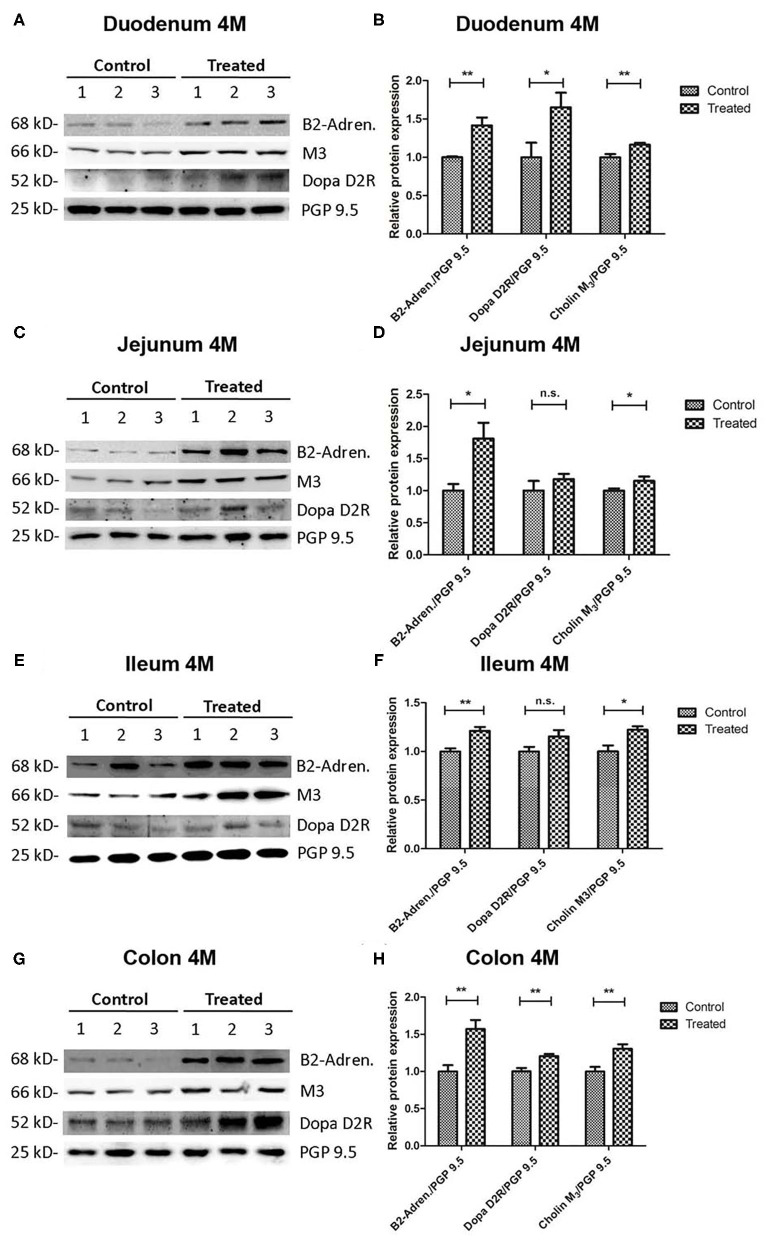
Four-Month rotenone exposure induces an up regulation of the adrenergic (ß2-subunit), cholinergic (M3-muscarinic), and dopaminergic (D2) receptors in the gut. Westenr blots in **(A,C,E,G)** show 3 representative examples per exposure group for the protein levels of adrenergic (ß2-subunit) (B2-Adren.), cholinergic (M3-muscarinic) (M3), and dopaminergic (D2) (Dopa D2R) receptors in duodenum **(A)**, jejunum **(C)**, ileum **(E)**, and colon **(G)**. The protein gene product 9.5 (PGP 9.5) was used as a panneuronal marker to determine the total neuronal population. Images were quantified using FIJI as described in the material and methods section. Protein expression for each marker was normalized to the amount of neuronal tissue in the sample by dividing it to the values obtained for PGP9.5 expression. Bar graphs in **(B,D,F,H)** show the quantification of the original blots (*n* = 5). Data was analyzed using a Student's *t*-test (Mann-Whitney Test). *, **, correspond to *P* < 0.05, and *P* < 0.01 respectively. n.s. non-significant. Error bars represent SEM in all graphics.

First we compared the expression of muscarinic cholinergic- (M3), dopaminergic- (D2R), and adrenergic- (ß2-subunit) receptors between rotenone-exposed and control mice normalized to the total amount of neurons (PGP9.5) (*n* = 5). These values were then normalized to the mean value in the control group. We could observe a region- and time-dependent up-regulation of all three kinds of receptors in rotenone-exposed mice. The expression of M3-muscarinic cholinergic receptors was up regulated in duodenum, jejunum, ileum and colon 2 and 4 months after rotenone exposure. The expression of adrenergic-(ß2-subunit) receptors was also up-regulated in duodenum, jejunum, ileum, and colon after rotenone exposure. Likewise, the expression of D2-dopaminergic-receptors was generally up-regulated in the duodenum, jejunum, ileum, and colon in rotenone-exposed mice (see Figures 9, 10 and Tables 9–11).

**Table 9:** Shows the mean values (mean), the standard error of the mean (SEM), and the significance (*p*) of the difference between control and rotenone exposed mice in the expression of the muscarinic cholinergic Receptor (M3) normalized to the total amount of neurons (PGP9.5) and to the mean of these values in the control group.

**M3-receptor**		**Control**		**Rotenone**		***p***
		**Mean**	**SEM**	**Mean**	**SEM**	
Duodenum	2M	1	0.068	1.187	0.091	**<0.01**
	4M	1	0.040	1.163	0.025	**<0.01**
Jejunum	2M	1	0.033	1.224	0.046	**<0.01**
	4M	1	0.033	1.151	0.068	**<0.05**
Ileum	2M	1	0.057	1.229	0.059	**<0.05**
	4M	1	0.062	1.223	0.035	**<0.05**
Colon	2M	1	0.042	1.166	0.456	**<0.05**
	4M	1	0.061	1.305	0.060	**<0.01**

*Bold values are the significant values*.

**Table 10:** Shows the mean values (mean), the standard error of the mean (SEM), and the significance (*p*) of the difference between control and rotenone exposed mice in the expression of the adrenergic Receptor (ß2-subunit) normalized to the total amount of neurons (PGP9.5) and to the mean of these values in the control group.

**ß2-subunit**		**Control**		**Rotenone**		***p***
		**Mean**	**SEM**	**Mean**	**SEM**	
Duodenum	2M	1	0.105	1.260	0.038	**<0.05**
	4M	1	0.012	1.425	0.104	**<0.01**
Jejunum	2M	1	0.065	1.174	0.059	>0.05
	4M	1	0.033	1.809	0.240	**<0.05**
Ileum	2M	1	0.040	1.181	0.091	>0.05
	4M	1	0.031	1.212	0.040	**<0.01**
Colon	2M	1	0.012	1.287	0.067	**<0.01**
	4M	1	0.084	1.570	0.122	**<0.01**

*Bold values are the significant values*.

**Table 11:** Shows the mean values (mean), the standard error of the mean (SEM), and the significance (*p*) of the difference between control and rotenone exposed mice in the expression of the dopaminergic Receptor (D2) normalized to the total amount of neurons (PGP9.5) and to the mean of these values in the control group.

**D2-receptor**		**Control**		**Rotenone**		***p***
		**Mean**	**SEM**	**Mean**	**SEM**	
Duodenum	2M	1	0.044	1.065	0.066	>0.05
	4M	1	0.190	1.653	0.189	**<0.05**
Jejunum	2M	1	0.060	1.283	0.100	**<0.05**
	4M	1	0.150	1.179	0.080	>0.05
Ileum	2M	1	0.005	1.151	0.078	**<0.01**
	4M	1	0.047	1.153	0.060	>0.05
Colon	2M	1	0.064	1.514	0.016	**<0.05**
	4M	1	0.046	1.205	0.029	**<0.01**

*Bold values are the significant values*.

### Rotenone Exposure Induces Modifications of Neuronal-Specific Markers in the Murine Intestine

In order to analyze the effect of rotenone on the enteric neurons of rotenone-exposed mice, we studied the expression of different neuronal subpopulation markers present in the duodenum, jejunum, ileum, and colon from vehicle- and rotenone-exposed mice. Our results show that rotenone induces changes in all tested regions. These alterations are region and exposure dependent.

#### Exposure to Rotenone Does Not Alter PGP9.5 in the Intestine of Mice Exposed for 2 or 4 Months

**Figure 11:**
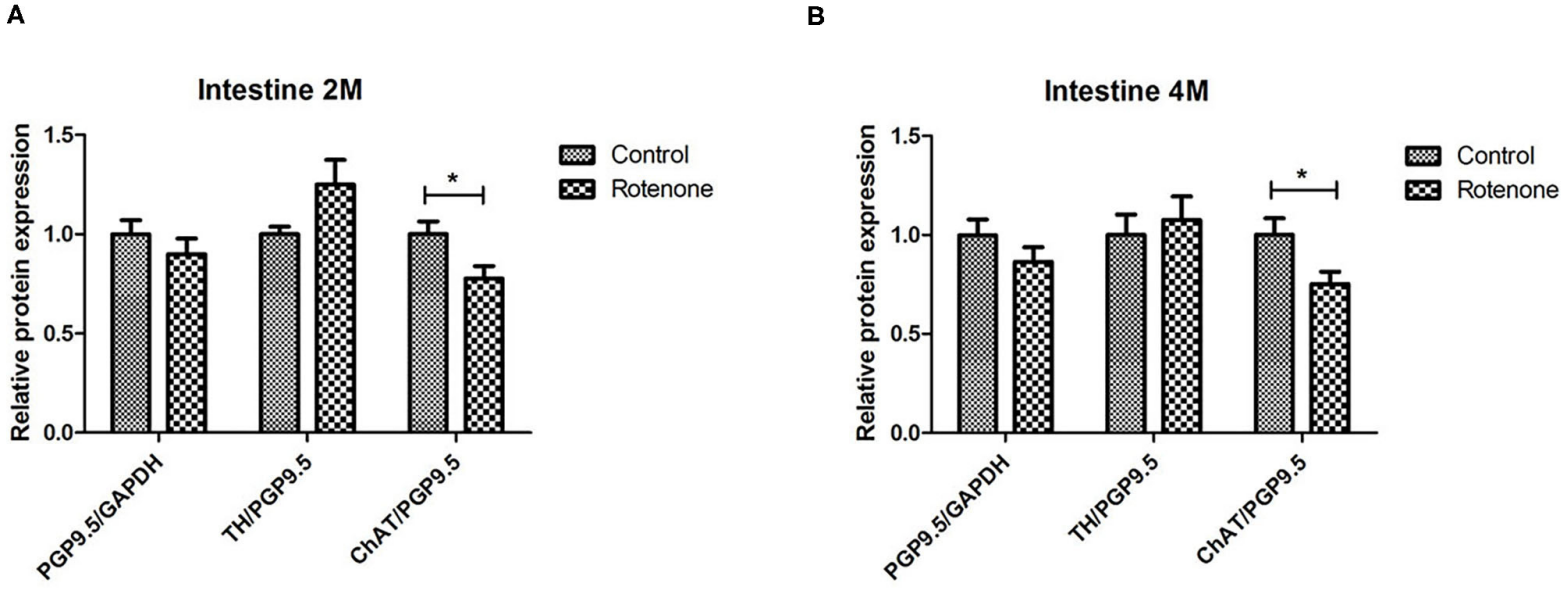
Overall effect of 2- and 4-month rotenone exposure on the expression of ChAT, TH, and PGP9.5 in the gut. Bar graph in **(A)** shows the protein expression levels of ChAT, TH and PGP9.5 in control and 2 months rotenone-exposed mice as the average of the values obtained for all regions. Bar graph in **(B)** shows the protein expression levels of ChAT, TH and PGP9.5 in 4 months control and rotenone-exposed mice as the average of the values obtained for all regions. ChAT and TH were normalized to the total neuronal tissue, determined by PGP9.5 expression levels. PGP9.5 was normalized to GAPDH. Data was analyzed using a Student's *t*-test (Mann-Whitney Test). *Corresponds to *P* < 0.05. n.s. non-significant. Error bars represent SEM in all graphics.

**Figure 12:**
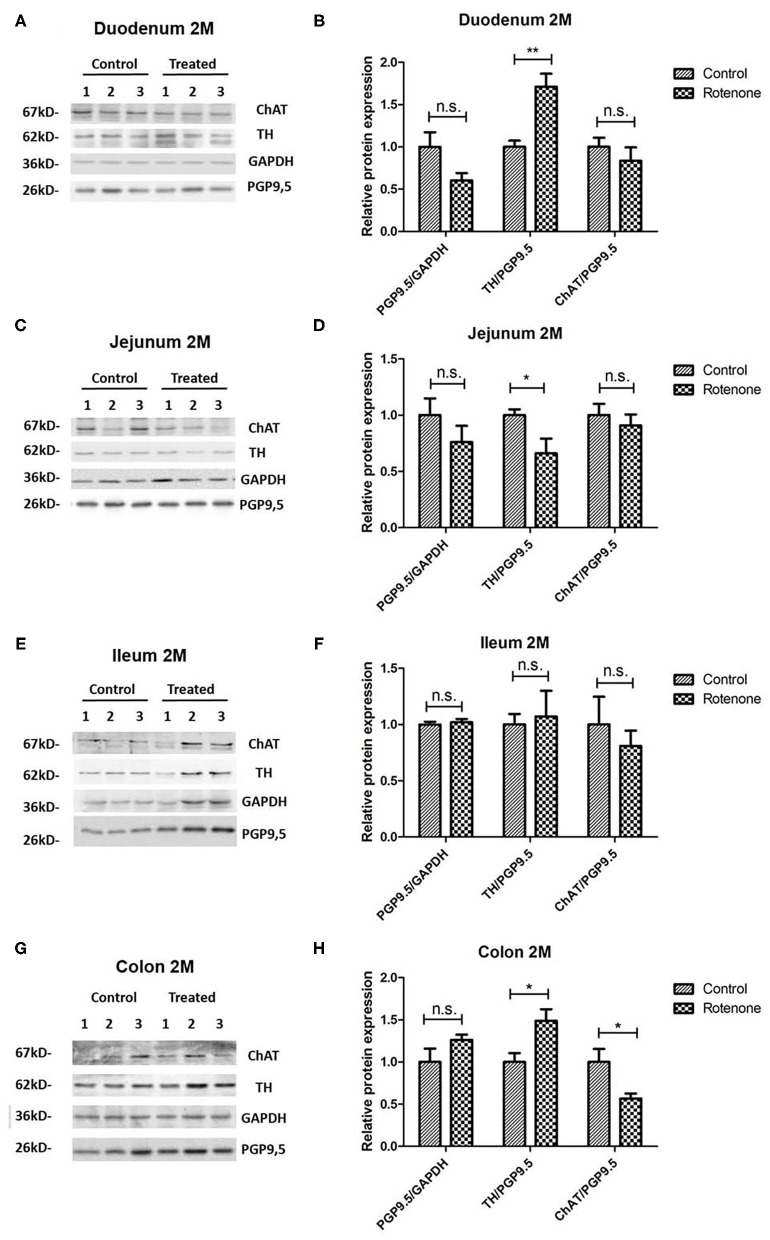
Effect of 2-month rotenone on the expression of ChAT, TH, and PGP9.5 in each intestinal region. Western blots in **(A,C,E,G)** show three representative examples per exposure group for the protein levels of ChAT, TH, GAPDH, and PGP9.5 in duodenum **(A)**, jejunum **(C)**, ileum **(E)**, and colon **(G)**. The protein gene product 9.5 (PGP 9.5) was used as a panneuronal marker to determine the ratio of enteric neuronal population within the sample. Images were quantified using FIJI as described in the material and methods section. ChAT and TH signals were normalized to the total amount of neuronal tissue in the sample by dividing it to the values obtained for PGP9.5 expression. The proportion of enteric neurons was determined by normalizing PGP9.5 signal to the total protein measured with GAPDH. Bar graphs in **(B,D,F,H)** show the quantification of the original blots (*n* = 5). Data was analyzed using a Student's *t*-test (Mann-Whitney Test). *, ** correspond to *P* < 0.05 and *P* < 0.01, respectively. n.s. non-significant. Error bars represent SEM in all graphics.

**Figure 13:**
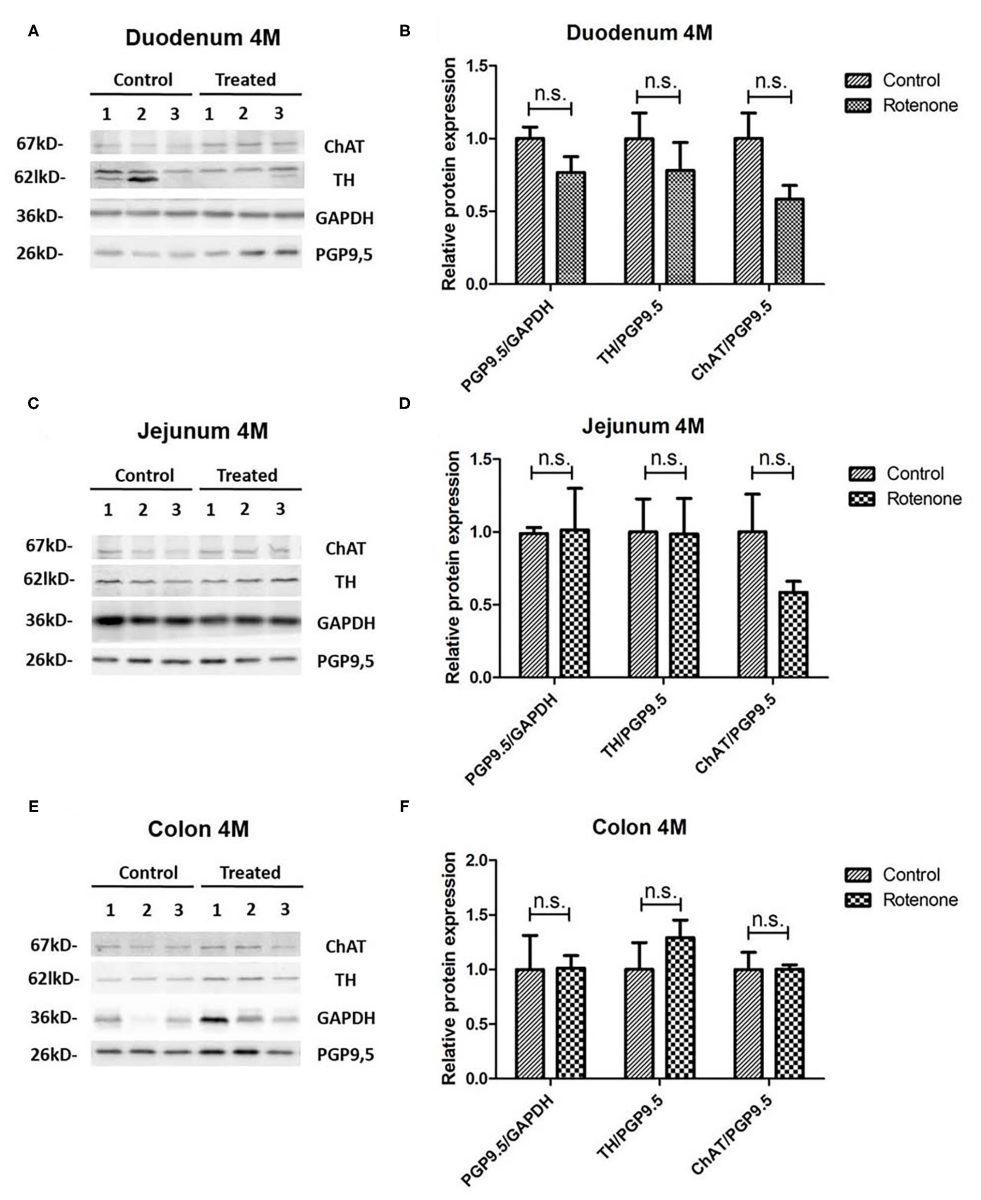
Effect of 4-month rotenone on the expression of ChAT, TH, and PGP9.5 in each intestinal region. Western blots in **(A,C,E)** show three representative examples per exposure group for the protein levels of ChAT, TH, GAPDH, and PGP9.5 in duodenum **(A)**, jejunum **(C)**, and colon **(E)**. The protein gene product 9.5 (PGP 9.5) was used as a panneuronal marker to determine the ratio of enteric neuronal population within the sample. Images were quantified using FIJI as described in the material and methods section. ChAT and TH signals were normalized to the total amount of neuronal tissue in the sample by dividing it to the values obtained for PGP9.5 expression. The proportion of enteric neurons was determined by normalizing PGP9.5 signal to the total protein measured with GAPDH. Bar graphs in **(B,D,F)** show the quantification of the original blots (*n* = 5). Data was analyzed using a Student's *t*-test. n.s. non-significant (Mann-Whitney Test). Error bars represent SEM in all graphics.

To study the effect on rotenone exposure on neuronal cells we analyzed the protein expression of PGP9.5, an ubiquitin carboxy-terminal hydrolase mainly expressed in neurons and we normalized it to the expression of Glycerinaldehyd-3-phosphat-Dehydrogenase (GAPDH), which is stably expressed among the different cell populations used here as a loading control. We compared the protein expression of PGP9.5 in all intestinal regions in 2 and 4 months-rotenone- and vehicle-exposed mice. Neither analyzing all regions together (see Figure 11) or separately (see Figures 12, 13) showed significant differences between exposure groups. We could observe minimal non-significant differences in the effect of rotenone exposure in the relative protein expression level of PGP9.5 between intestinal regions (see Figures 12, 13). Unfortunately, we ran out of protein extract from ileum 4 months, so that no statistical analysis was possible for any of the markers tested (see Table 12).

**Table 12:** Shows the mean values (mean), the standard error of the mean (SEM), and the significance (*p*) of the difference between control and rotenone exposed mice in the expression of PGP 9.5 normalized to the mean of the control group.

**PGP9.5**		**Control**		**Rotenone**		***p***
		**Mean**	**SEM**	**Mean**	**SEM**	
Duodenum	2M	1	0.17	0.60	0.09	>0.05
	4M	1	0.08	0.77	0.11	>0.05
Jejunum	2M	1	0.15	0.76	0.15	>0.05
	4M	1	0.03	1.01	0.28	>0.05
Ileum	2M	1	0.02	1.02	0.03	>0.05
Colon	2M	1	0.16	1.26	0.06	>0.05
	4M	1	0.31	1.01	0.11	>0.05

#### Exposure to Rotenone for 2 and 4 Months Decreases Enteric Choline Acetyltransferase Levels

A reduction in the cholinergic innervation of the gut has been described in patients (18). Therefore, we analyzed the protein expression pattern of choline acetyltransferase (ChAT), a protein expressed in cholinergic neurons, in the duodenum, jejunum, ileum and colon of control and exposed mice. When all regions were analyzed together, we observed a significant decrease in the amount of enteric ChAT expressed in rotenone-exposed mice when compared to controls after 2 months and 4 months of rotenone exposure (see Figures 11A,B). Taken separately, all intestinal regions from rotenone-exposed mice showed a decrease in ChAT expression levels when compared to controls after 2 months of exposure, which was significant only in the colon (see Figure 12). After 4 months of exposure, we observed a non-significant decrease in the amount of enteric ChAT expressed in rotenone-exposed mice when compared to controls in the duodenum and the jejunum but not in the colon (see Figure 13 and Table 13 for a summary of the results).

**Table 13:** Shows the mean values (mean), the standard error of the mean (SEM), and the significance (*p*) of the difference between control and rotenone exposed mice in the expression of choline acetyltransferase (ChAT) normalized to the total amount of neurons (PGP9.5) and to the mean of these values in the control group.

**ChAT**		**Control**		**Rotenone**		***p***
		**Mean**	**SEM**	**Mean**	**SEM**	
Duodenum	2M	1	0.11	0.84	0.16	>0.05
	4M	1	0.17	0.58	0.09	>0.05
Jejunum	2M	1	0.10	0.90	0.10	>0.05
	4M	1	0.26	0.58	0.07	>0.05
Colon	2M	1	0.15	0.57	0.06	**<0.05**
	4M	1	0.26	2.00	0.04	>0.05
All regions	2M	1	0.06	0.77	0.06	**<0.05**
	4M	1	0.08	0.75	0.06	**<0.05**

*Bold values are the significant values*.

#### Rotenone Exposure Modifies Tyrosine Hydroxylase Protein Levels in the Intestine of Rotenone-Exposed Mice

We also analyzed the relative protein level of the tyrosine hydroxylase (TH/PGP9.5), implicated in the synthesis of the catecholamines dopamine, epinephrine and norepinephrine in the nervous system. We compared the protein expression of TH in all different segments from 2 and 4 months rotenone-exposed mice and controls. After 2 and 4 months of exposure, we observed no differences in the protein expression of TH in rotenone-exposed mice compared to control mice when all regions of the intestine were analyzed together (see Figure 11). However, when taken separately, TH levels were significantly higher in rotenone-exposed samples compared to control samples in duodenum and colon after two months of rotenone exposure. In the meantime, no difference was observed in the ileum and TH levels were lower in the jejunum of rotenone-exposed mice, highlighting the region-specific effect of rotenone exposure in the intestine. After 4 months of rotenone exposure we observed a non-significant increase in TH protein expression in the colon of rotenone-exposed mice compared to control mice whereas this expression was either similar in the jejunum or lower in the duodenum of rotenone-exposed mice compared to control mice (see Figures 12, 13 and Table 14).

**Table 14:** Shows the mean values (mean), the standard error of the mean (SEM), and the significance (*p*) of the difference between control and rotenone exposed mice in the expression of tyrosine hydroxylase (TH) normalized to the total amount of neurons (PGP9.5) and to the mean of these values in the control group.

**TH**		**Control**		**Rotenone**		***p***
		**Mean**	**SEM**	**Mean**	**SEM**	
Duodenum	2M	1	0.07	1.71	0.15	**<0.01**
	4M	1	0.17	0.78	0.19	>0.05
Jejunum	2M	1	0.05	0.66	0.13	**<0.05**
	4M	1	0.23	0.98	0.24	>0.05
Ileum	2M	1	0.09	1.07	0.23	>0.05
Colon	2M	1	0.10	1.48	0.14	**<0.05**
	4M	1	0.24	1.29	0.16	>0.05
All regions	2M	1	0.06	0.78	0.06	>0.05
	4M	1	0.08	0.75	0.06	>0.05

*Bold values are the significant values*.

#### Changes in the Level of $\alpha_{4}\beta_{2}^{*}$ Nicotinic Acetylcholine Receptors in the Gastrointestinal Tract Are Not Detectable by PET After 2 or 4 Months Exposure to Rotenone

**Figure 14:**
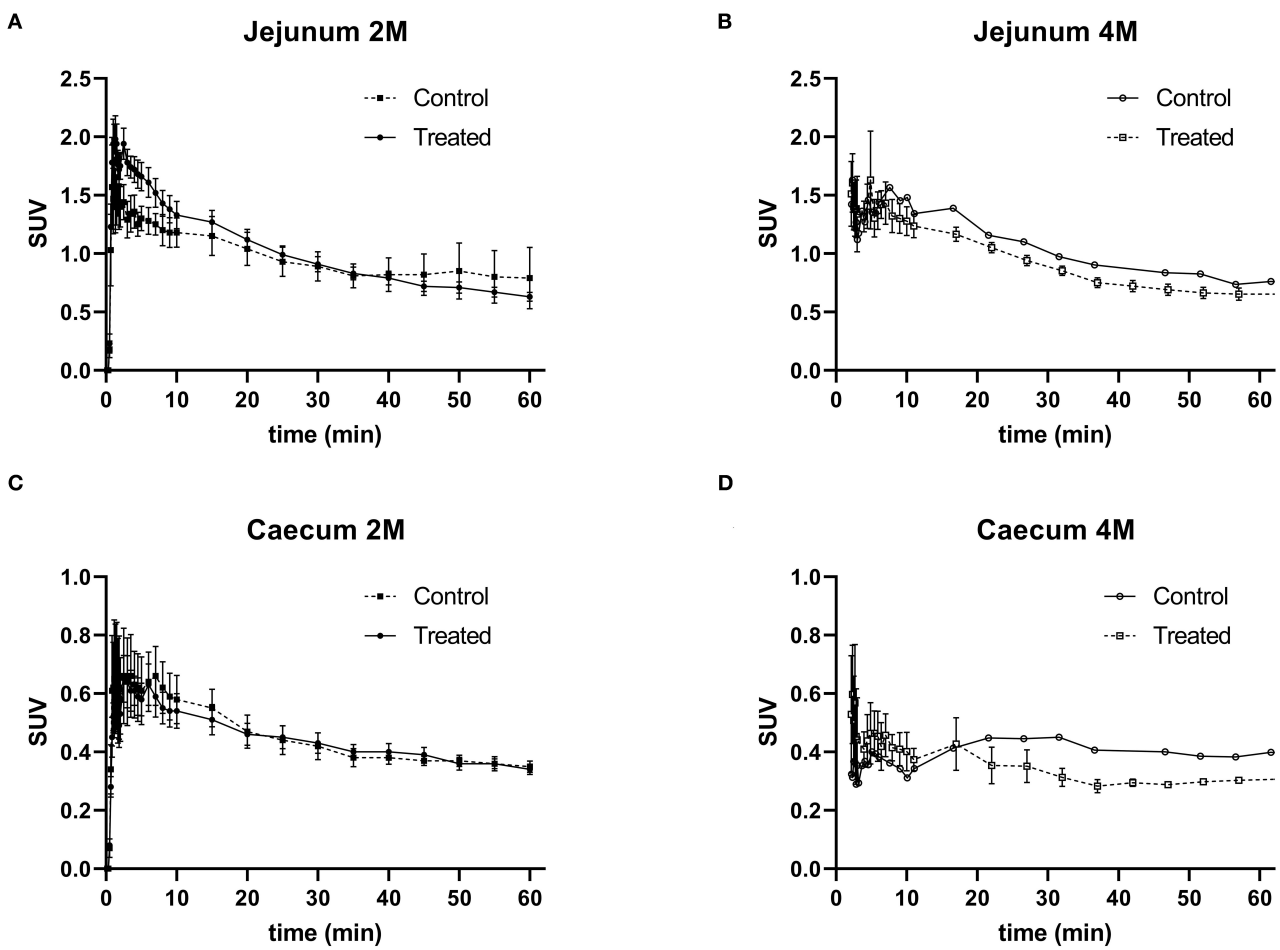
(–)-[^18^F]Flubatine-PET imaging of enteric $\alpha_{4}\beta_{2}^{*}$ nicotinic acetylcholine receptor density after 4 months exposure to rotenone. Time-activity curves of the jejunum after exposure to rotenone of 2 months (2M) **(A)** or 4 months (4M) **(B)**; time-activity curves of the caecum after exposure to rotenone of 2 months (2M) **(C)** or 4 months (4M) **(D)**; after injection of (–)-[^18^F]Flubatine in C57/BL6J mice control or treated. Error bars represent SEM.

For the *in vivo* PET imaging two regions were selected for receptor density evaluation: the jejunum and the caecum. After 2 months of exposure to rotenone no alteration of $\alpha_{4}\beta_{2}^{*}$ nicotinic acetylcholine receptors density could be detected with PET imaging in the jejunum as well as in the caecum region when compared to controls (see Figures 14A–C). After 4 months of exposure to rotenone we observed a non-significant decrease in the density of enteric $\alpha_{4}\beta_{2}^{*}$ nicotinic acetylcholine receptors in the jejunum and the caecum when compared to controls (see Figures 14B–D).

## Discussion

Non-motor symptoms in Parkinson's disease are widely spread among patients. The prevalence varies depending on the type of symptom and, unlike motor symptoms, they are not always observed in PD patients (19–25). According to Braak's pathological staging, most of the impaired nervous structures giving rise to non-motor symptoms (e.g., enteric, sympathetic, or parasympathetic systems) are affected in very early stages of the disease and show PD-pathology in the form of LB and LN (1). Specifically, gastrointestinal alterations can appear up to 15 years before the appearance of the motor symptoms in PD patients. In this study we focused on the analysis of gastrointestinal symptoms in a toxic model of Parkinson's disease based on the oral administration of rotenone. In previous studies we have shown that the exposure to oral administered rotenone induces PD-like changes in the ENS and triggers the progression of PD throughout the nervous system until it reaches the substantia nigra (11, 12). Complementarily, the results of the present study suggest that the gastrointestinal alterations present in PD patients could also be explained by such an exposure to environmental toxins due to the modification of the neuronal population, the impairment of the vagal and sympathetic innervation of the ENS and the subsequent up-regulation in the expression of cholinergic, adrenergic and dopaminergic receptors within the intestine in a region-, age- and exposure-dependent manner. Interestingly, the latter changes appear already at early time-points after rotenone administration (2 months) before the onset of motor symptoms (that in this animal model occur after 3 months of exposure), thus, mimicking the progression pattern observed in PD patients. The oral exposure to rotenone in this model mimics a realistic exposure to pesticide as it occurs in the general population and would also explain that the intestinal symptoms are the first to appear.

In two recent studies, it was shown that rotenone exposure reduces the sympathetic noradrenergic (13) and the vagal cholinergic (14) innervation of the gut. In order to determine whether enteric neurotransmitters did also play a role in these alterations, we analyzed the differences in the total enteric neuronal population and its subpopulations between control and rotenone-exposed mice. Although there was a general decrease in the expression of PGP9.5, ChAT, and TH in rotenone-exposed mice (suggesting a general neuronal loss) when analyzed by intestinal regions, this decrease was not significant in most regions. Moreover, the changes observed in the different analyzed regions did not remain constant with time, even under exposure, suggesting the presence of neuronal plasticity in response to pesticide exposure. This could also provide an explanation for the contradictory results between regions and within the same region between different exposure times. In this regard, it was recently shown that the enteric nervous system is able to regenerate through neurogenesis (26, 27), a process already described in other degenerative processes of the gut. These results would also agree with a recent study published by Annerino et al. (28). In this study they did not observe a neuronal loss in the myenteric ganglia of PD patients when compared to controls.

Overall these results support the hypothesis that the sympathetic and cholinergic innervation of the gut plays a main role in the pathophysiology of the functional gastrointestinal alterations and that the role of the intrinsic neurotransmitters is, if something, marginal and transitory. How the extrinsic innervation of the gut was able to alter the expression of TH in the gut remains unclear. We speculate that the activation of postsynaptic enteric neurons by ACh and NA in the ENS leads to the activation of dopaminergic neurons and in the absence of this input there is an up-regulation in the expression of enteric dopaminergic receptors, but it needs further clarification.

The early appearance, the wide consistency across time and intestinal regions of this up-regulation and the accessibility to obtain biopsies (specially for the colon) make these radiological markers, in combination with the diagnosis of other premotor symptoms such as idiopathic REM sleep behavior disorder, ideal for the early detection of PD. Therefore, we analyzed if there were variations in the expression of the α4β2^*^ nicotinic cholinergic receptor using (–)-[^18^F]Flubatine as a radiotracer. The reasons for using this radiotracer being that (–)-[^18^F]Flubatine has been previously used in the clinic as a radiological marker for Alzheimer's disease (29), that it is not eliminated through the intestine, an important pharmacokinetic characteristic when investigating expression levels in the gut and that we had access to it. The results only showed a non-significant reduction in the PET signal intensity. In this regard, it is important to note that the metabolism of (–)-[^18^F]Flubatine is higher in mice than human (30) and, together with the peristaltic movement, could be confounding factors for the PET data evaluation. Additionally, the PET signal cannot be normalized to the amount of neurons present in that region as was done with the western-blot results. Therefore, it could be possible that the signal observed is the result of an up regulated receptor expression in a diminished neuronal population. Finally, it has been shown that α4β2^*^ nicotinic cholinergic receptors expressed in the brain have a paradoxical effect to stimulation with nicotine, as they are up regulated by it (31, 32). It is therefore possible that a reduction in the cholinergic input could lead to a down regulation of this receptor instead of an up regulation. Regretfully, determining the expression levels of this specific receptor was not possible with western-blot. There are only antibodies against the subunits (α4 or β2) and it has been shown that there are many different subtypes of nicotinic cholinergic receptors using at least on of these subunits (33). Therefore, these antibodies were not useful for our purposes as we would not have been able to specifically target our protein of interest. Overall, these findings may be in favor of a cholinergic denervation as described in patients (30), but further investigation using radiotracers targeting other receptors would be needed in order to discriminate properly. These results suggest that using a suitable radiotracer, or a combination of them, together with other diagnostic techniques (i.e., EEG for REM-sleep disorders or high-speed MRI for gastric motility alterations) could increase the sensibility and specificity in the early diagnosis of PD, which would allow for an earlier therapeutic intervention and a better outcome.

## Data Availability Statement

The original contributions presented in the study are included in the article/Supplementary Material, further inquiries can be directed to the corresponding author/s.

## Ethics Statement

The animal study was reviewed and approved by the Saxonian Committee for Animal Research in Germany and the Bavarian Committee for Animal Research in Germany.

## Author Contributions

FP-M designed the study. YD exposed the mice. UR and FP-M designed the contractility experiments. RK, YD, and FP-M performed the contractility experiments. KB wrote the Matlab quantification program. UR, GS, KB, and FP-M analyzed the contractility results. AS, QS, and FP-M performed the western-blots. CV-B, AS, QS, RF, and FP-M analyzed the western-blot results. PB, MT, MK, and DG performed the PET and analyzed the PET data. MD, KB, RF, and FP-M wrote and critically corrected the manuscript. All authors contributed to the article and approved the submitted version.

## Conflict of Interest

The authors declare that the research was conducted in the absence of any commercial or financial relationships that could be construed as a potential conflict of interest.
