# Supplementary material for: Pathophysiological Changes in the Enteric Nervous System of Rotenone-Exposed Mice as Early Radiological Markers for Parkinson's Disease
Source: Front Neurol. 2021 Mar 22;12:642604. doi: 10.3389/fneur.2021.642604 (PMC8030242; doi:10.3389/fneur.2021.642604)
Supplement: Supplementary file 1 [file Data_Sheet_1.PDF]

Supp. Fig. 1

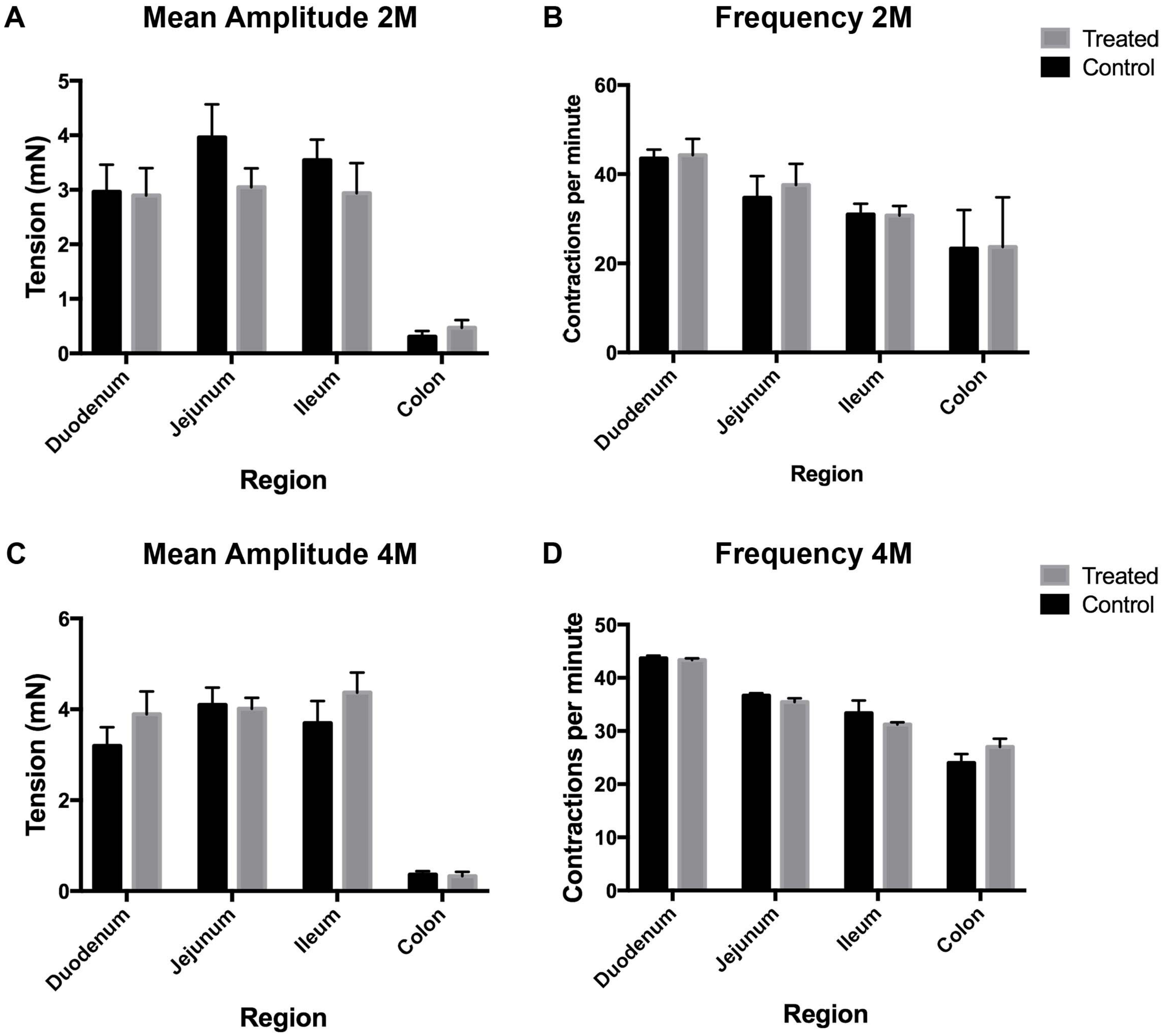
A
Mean Amplitude 2M
Tension (mN)
0
1
2
3
4
5
Duodenum
Jejunum
Ileum
Colon
Region
B
Frequency 2M
Contractions per minute
0
20
40
60
Duodenum
Jejunum
Ileum
Colon
Region
Treated
Control
C
Mean Amplitude 4M
Tension (mN)
0
2
4
6
Duodenum
Jejunum
Ileum
Colon
Region
D
Frequency 4M
Contractions per minute
0
10
20
30
40
50
Duodenum
Jejunum
Ileum
Colon
Region
Treated
Control

Supp. Fig. 2

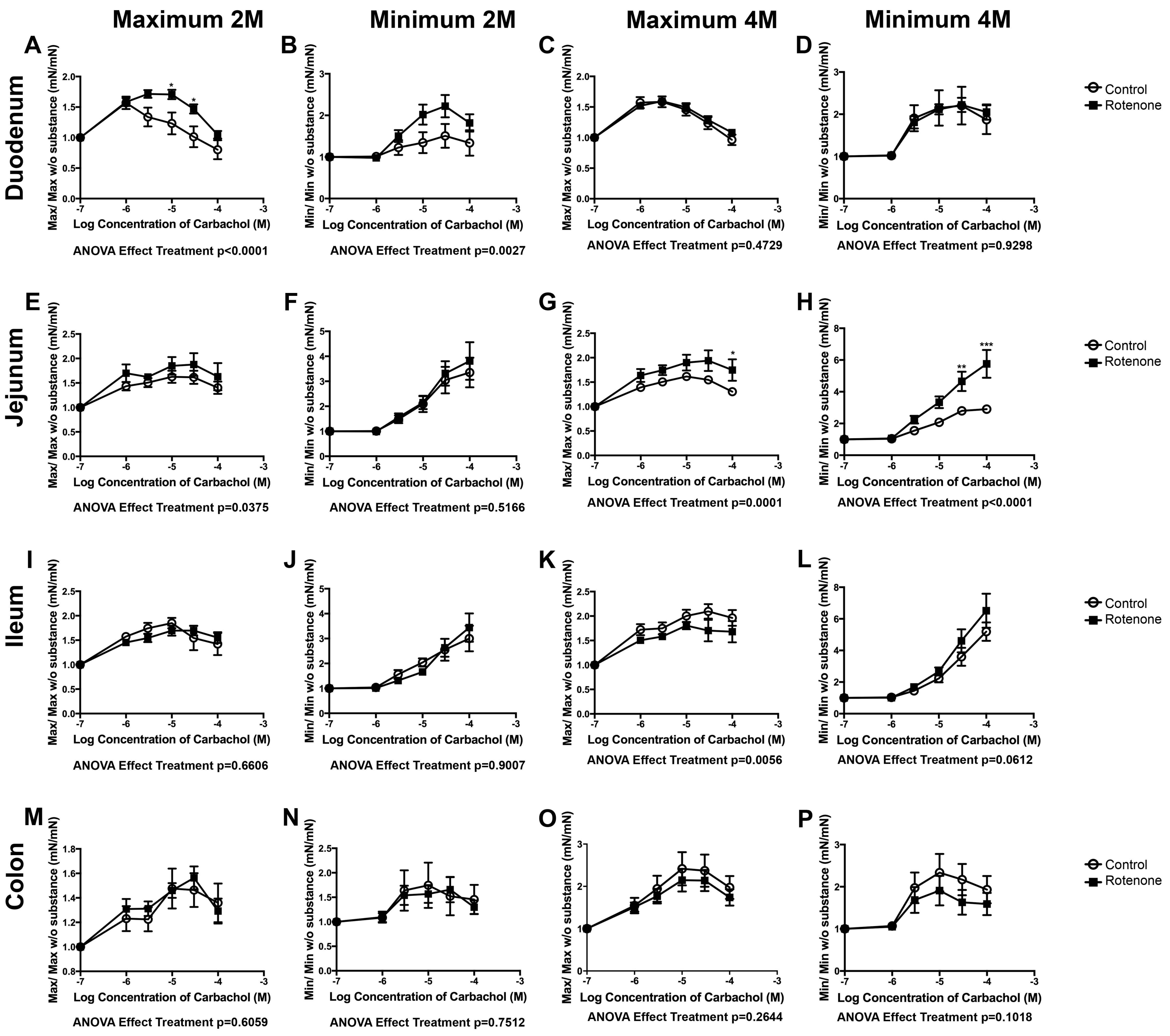

Maximum 2M
Minimum 2M
Maximum 4M
Minimum 4M
Duodenum
Jejunum
Ileum
Colon
Control
Rotenone
Max/ Max w/o substance (mN/mN)
Min/ Min w/o substance (mN/mN)
Log Concentration of Carbachol (M)
A
ANOVA Effect Treatment p<0.0001
B
ANOVA Effect Treatment p=0.0027
C
ANOVA Effect Treatment p=0.4729
D
ANOVA Effect Treatment p=0.9298
E
ANOVA Effect Treatment p=0.0375
F
ANOVA Effect Treatment p=0.5166
G
ANOVA Effect Treatment p=0.0001
H
ANOVA Effect Treatment p<0.0001
I
ANOVA Effect Treatment p=0.6606
J
ANOVA Effect Treatment p=0.9007
K
ANOVA Effect Treatment p=0.0056
L
ANOVA Effect Treatment p=0.0612
M
ANOVA Effect Treatment p=0.6059
N
ANOVA Effect Treatment p=0.7512
O
ANOVA Effect Treatment p=0.2644
P
ANOVA Effect Treatment p=0.1018

Supp. Fig. 3

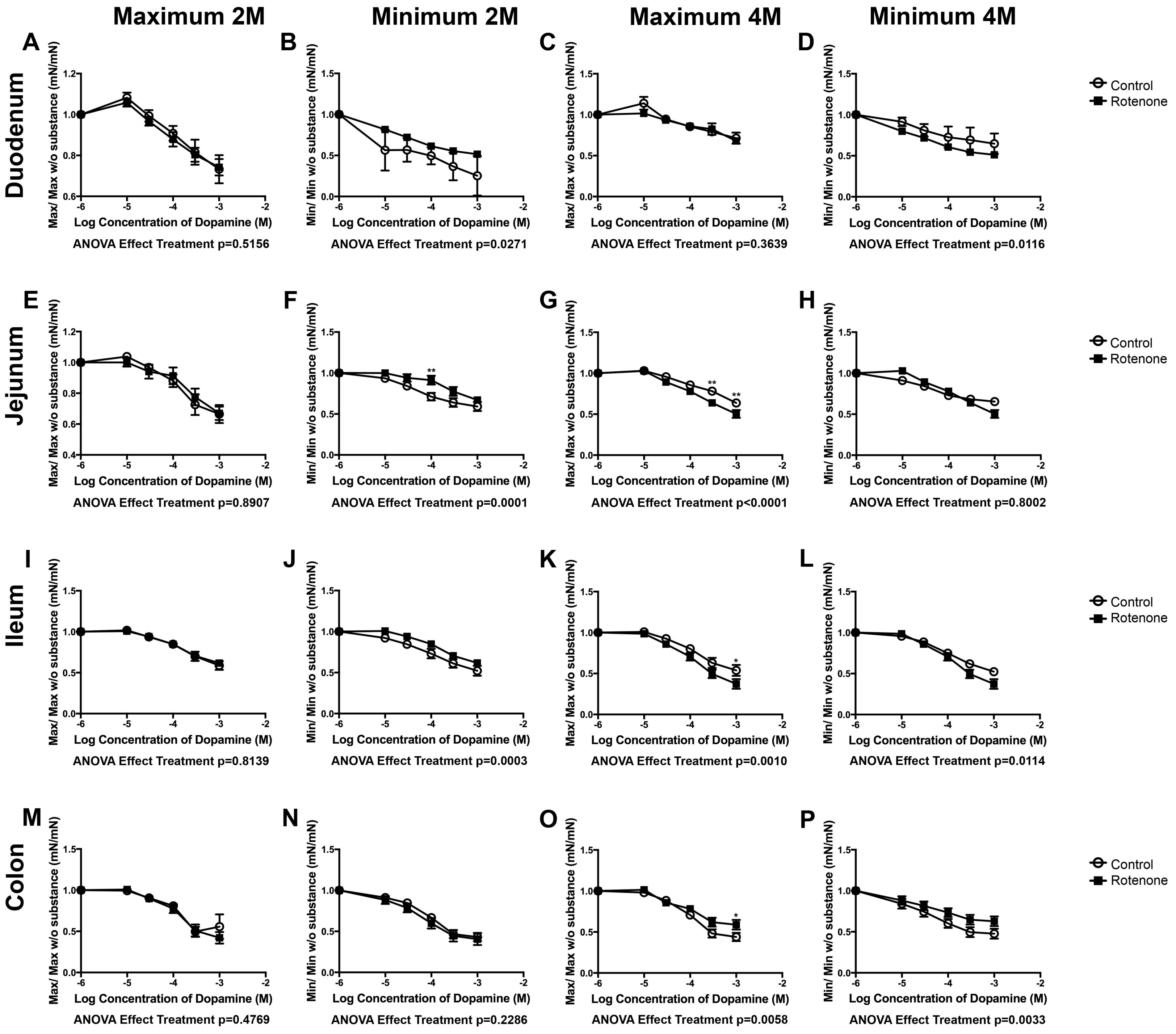
Maximum 2M
Minimum 2M
Maximum 4M
Minimum 4M
Duodenum
Jejunum
Ileum
Colon
Control
Rotenone
Max/ Max w/o substance (mN/mN)
Min/ Min w/o substance (mN/mN)
Log Concentration of Dopamine (M)
A ANOVA Effect Treatment p=0.5156
B ANOVA Effect Treatment p=0.0271
C ANOVA Effect Treatment p=0.3639
D ANOVA Effect Treatment p=0.0116
E ANOVA Effect Treatment p=0.8907
F ANOVA Effect Treatment p=0.0001
G ANOVA Effect Treatment p<0.0001
H ANOVA Effect Treatment p=0.8002
I ANOVA Effect Treatment p=0.8139
J ANOVA Effect Treatment p=0.0003
K ANOVA Effect Treatment p=0.0010
L ANOVA Effect Treatment p=0.0114
M ANOVA Effect Treatment p=0.4769
N ANOVA Effect Treatment p=0.2286
O ANOVA Effect Treatment p=0.0058
P ANOVA Effect Treatment p=0.0033

Supp. Fig. 4

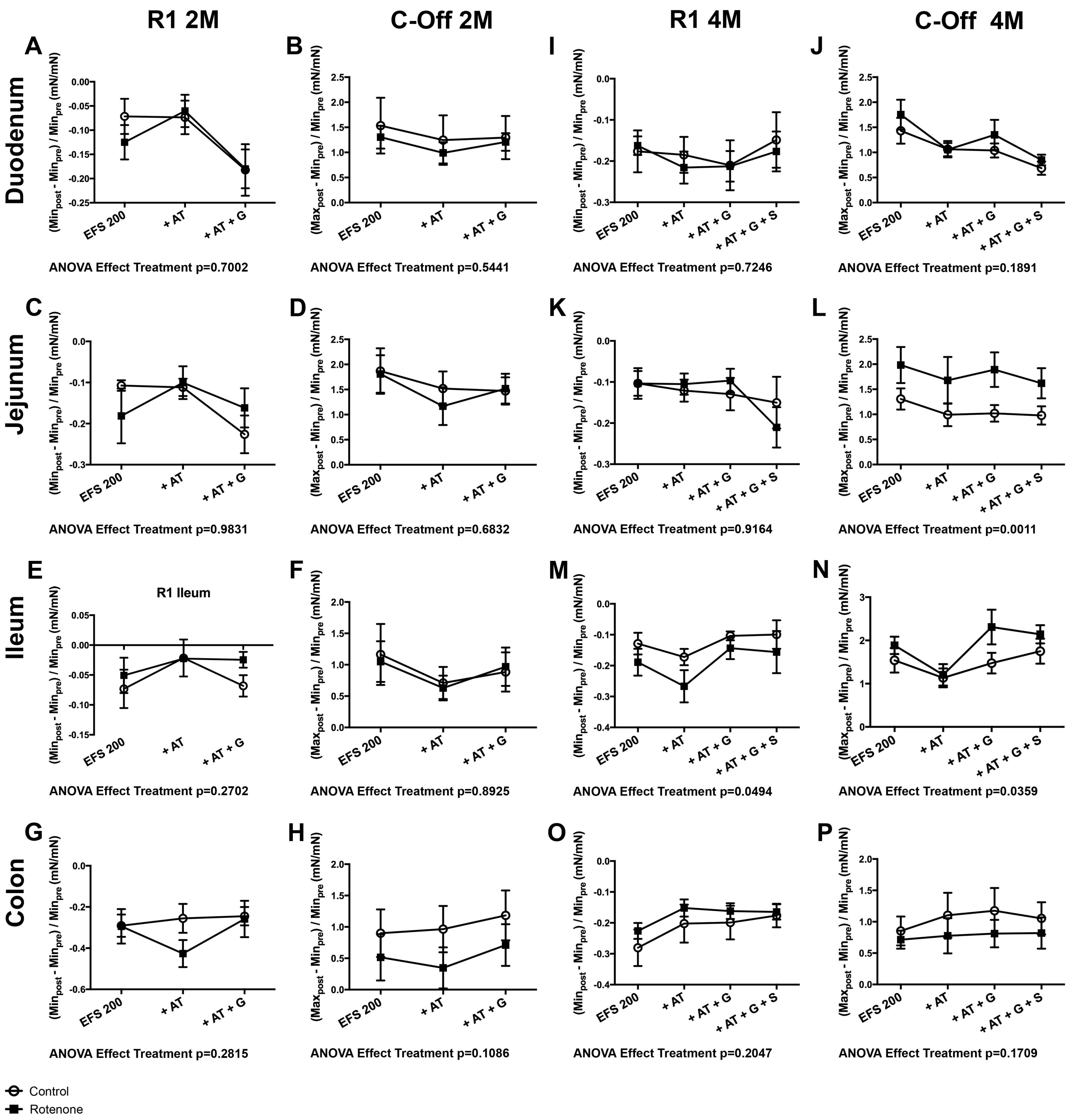
R1 2M
C-Off 2M
R1 4M
C-Off 4M
Duodenum
Jejunum
Ileum
Colon
A
B
I
J
C
D
K
L
E
F
M
N
G
H
O
P
R1 Ileum
$(Min_{post} - Min_{pre}) / Min_{pre}$ (mN/mN)
$(Max_{post} - Min_{pre}) / Min_{pre}$ (mN/mN)
EFS 200
+ AT
+ AT + G
+ AT + G + S
ANOVA Effect Treatment p=0.7002
ANOVA Effect Treatment p=0.5441
ANOVA Effect Treatment p=0.7246
ANOVA Effect Treatment p=0.1891
ANOVA Effect Treatment p=0.9831
ANOVA Effect Treatment p=0.6832
ANOVA Effect Treatment p=0.9164
ANOVA Effect Treatment p=0.0011
ANOVA Effect Treatment p=0.2702
ANOVA Effect Treatment p=0.8925
ANOVA Effect Treatment p=0.0494
ANOVA Effect Treatment p=0.0359
ANOVA Effect Treatment p=0.2815
ANOVA Effect Treatment p=0.1086
ANOVA Effect Treatment p=0.2047
ANOVA Effect Treatment p=0.1709
Control
Rotenone

Supp. Fig. 5

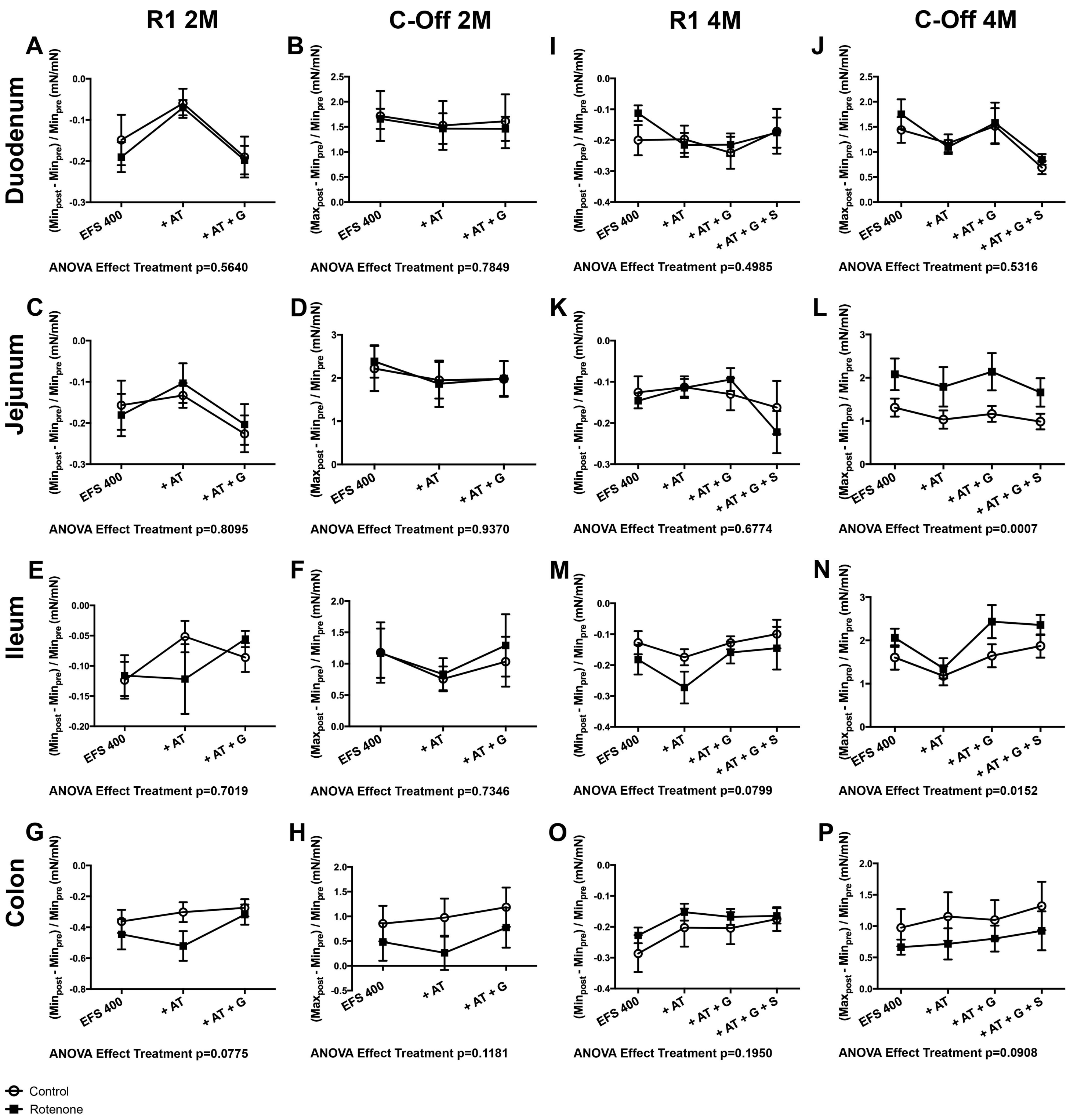

R1 2M
C-Off 2M
R1 4M
C-Off 4M
Duodenum
Jejunum
Ileum
Colon
A
B
I
J
C
D
K
L
E
F
M
N
G
H
O
P
(Min post - Min pre) / Min pre (mN/mN)
(Max post - Min pre) / Min pre (mN/mN)
EFS 400
+ AT
+ AT + G
+ AT + G + S
ANOVA Effect Treatment p=0.5640
ANOVA Effect Treatment p=0.7849
ANOVA Effect Treatment p=0.4985
ANOVA Effect Treatment p=0.5316
ANOVA Effect Treatment p=0.8095
ANOVA Effect Treatment p=0.9370
ANOVA Effect Treatment p=0.6774
ANOVA Effect Treatment p=0.0007
ANOVA Effect Treatment p=0.7019
ANOVA Effect Treatment p=0.7346
ANOVA Effect Treatment p=0.0799
ANOVA Effect Treatment p=0.0152
ANOVA Effect Treatment p=0.0775
ANOVA Effect Treatment p=0.1181
ANOVA Effect Treatment p=0.1950
ANOVA Effect Treatment p=0.0908
Control
Rotenone

Supplementary figure 1: Mean Amplitude and Frequency of contractions in the regions of the intestine after 2 and 4 months of exposure to rotenone. Bar graphics in **A** to **D** show the comparison of the Mean Amplitude (**A** and **C**) and the Frequency of contraction (**B** and **D**) after 2 months (**A** and **B**) and 4 months (**C** and **D**) of rotenone exposure. No significant difference was observed in any of the parameters analyzed. Error bars represent SEM.

Supplementary figure 2: Comparison between the concentration curves of carbachol and its effects on the different regions of the intestine between control and rotenone exposed mice. Graphics in A to P show the effect of different concentrations of carbachol on the maximal exerted contractility force (Max) first and third columns and on the intestinal muscle tone (Min) second and fourth columns in two months (2M) and four months (4M) vehicle (empty circle) and rotenone exposed (black square) mice. *, *** correspond to $P<0.05$ and $P<0.001$ respectively. Error bars represent SEM.

Supplementary figure 3: Comparison between the concentration curves of dopamine and its effects on the different regions of the intestine between control and rotenone exposed mice. Graphics in A to P show the effect of different concentrations of dopamine on the maximal exerted contractility force (Max) first and third columns and on the intestinal muscle tone (Min) second and fourth columns in two months (2M) and four months (4M) vehicle (empty circle) and rotenone exposed (black square) mice. ** correspond to $P<0.01$. Error bars represent SEM.

Supplementary figure 4: Comparison of the effect of a 200 µs electric field stimulation (EFS 200) on the different intestinal regions after 2 and 4 months of rotenone exposure in mice. Graphics in A to P show variations in the R1 (first and third columns) and C-Off (second and fourth columns) responses to the EFS 200 alone and after the addition of AT alone, AT and G or AT, G and S (serotonin only after 4 months of exposure). The overall response was compared between control and rotenone exposed mice with a 2-way ANOVA. Significance is written under each graphic.

Supplementary figure 5: Comparison of the effect of a 400 µs electric field stimulation (EFS 400) on the different intestinal regions after 2 and 4 months of rotenone exposure in mice. Graphics in A to P show variations in the in the R1 (first and third columns) and C-Off (second and fourth columns) responses to the EFS 400 alone and after the addition of AT alone, AT and G or AT, G and S (serotonin only after 4 months of exposure). The overall response was compared between control and rotenone exposed mice with a 2-way ANOVA. Significance is written under each graphic.

Table 1

Carbachol		p mean amplitude	p maximum	p minimum
		2-way ANOVA Treatment p value		
Duodenum	2M	**<0,001**	**<0,001**	**0,003**
	4M	0,263	0,473	0,930
Jejunum	2M	**<0,001**	**0,038**	0,517
	4M	**<0,001**	**<0,001**	**<0,001**
Ileum	2M	0,009	0,660	0,901
	4M	0,288	**0,006**	0,061
Colon	2M	0,085	0,606	0,751
	4M	**0,043**	0,264	0,102

Table 2

Dopamine		p mean amplitude	p maximum	p minimum
		2-way ANOVA Treatment p value		
Duodenum	2M	0,395	0,516	**0,027**
	4M	**0,022**	0,364	**0,012**
Jejunum	2M	0,689	0,891	**<0,001**
	4M	**0,012**	**<0,001**	0,800
Ileum	2M	0,890	0,814	**<0,001**
	4M	**0,009**	**0,001**	**0,011**
Colon	2M	0,186	0,477	0,229
	4M	0,286	**0,006**	**0,003**

Table 3

R1 AT		Control		p	Rotenone		p	p Control vs Rotenone
		Mean	SEM		Mean	SEM		
Duodenum	2M EFS 200	315,191	175,939	0,249	116,545	85,781	0,851	0,333
	2M EFS 400	41,398	20,512	**0,017**	44,004	18,337	**0,012**	0,926
	4M EFS 200	146,393	45,084	0,290	153,464	41,517	0,216	0,148
	4M EFS 400	168,833	69,876	0,311	215,126	92,574	0,232	0,342
Jejunum	2M EFS 200	118,449	33,240	0,591	72,171	42,528	0,528	0,397
	2M EFS 400	155,139	67,374	0,432	66,621	31,924	0,349	0,272
	4M EFS 200	201,091	49,656	0,059	81,788	18,877	0,349	**0,018**
	4M EFS 400	134,984	42,752	0,425	83,759	13,506	0,247	0,499
Ileum	2M EFS 200	341,479	280,242	0,409	122,493	46,901	0,642	0,459
	2M EFS 400	30,042	21,168	**0,008**	136,076	36,440	0,346	**0,031**
	4M EFS 200	257,809	102,348	0,121	185,114	57,208	0,156	0,116
	4M EFS 400	59,825	6,443	**0,033**	155,883	31,187	0,092	0,908
Colon	2M EFS 200	80,848	15,441	0,243	230,193	98,750	0,217	0,166
	2M EFS 400	59,825	6,443	0,283	122,885	16,826	0,204	0,107
	4M EFS 200	63,827	14,303	**0,022**	70,743	10,085	**0,010**	0,698
	4M EFS 400	63,568	14,458	**0,022**	70,603	9,899	**0,009**	0,693

Table 4

C-Off AT		Control		p	Rotenone		p	p Control vs Rotenone
		Mean	SEM		Mean	SEM		
Duodenum	2M EFS 200	79,173	14,143	0,171	73,393	11,249	**0,040**	0,756
	2M EFS 400	95,322	20,113	0,821	90,426	18,887	0,623	0,863
	4M EFS 200	81,353	9,268	**0,049**	65,884	6,538	**<0,001**	0,185
	4M EFS 400	88,081	8,791	0,169	68,931	6,566	**<0,001**	0,097
Jejunum	2M EFS 200	95,973	15,359	0,799	78,510	32,023	0,517	0,634
	2M EFS 400	98,213	15,010	0,908	75,032	14,189	0,109	0,288
	4M EFS 200	68,503	10,650	**0,009**	79,165	10,480	0,064	0,486
	4M EFS 400	74,035	7,839	**0,004**	82,448	10,150	0,103	0,521
Ileum	2M EFS 200	73,371	25,338	0,318	65,949	12,992	**0,026**	0,8
	2M EFS 400	80,838	20,061	0,362	76,370	10,713	0,052	0,848
	4M EFS 200	81,073	21,466	0,363	59,825	6,443	**<0,001**	0,334
	4M EFS 400	76,077	14,726	0,104	63,085	6,077	**<0,001**	0,408
Colon	2M EFS 200	99,826	36,553	0,996	27,022	11,878	**<0,001**	0,087
	2M EFS 400	94,823	47,659	0,916	-16,350	27,606	**0,002**	0,08
	4M EFS 200	109,166	16,862	0,594	97,017	13,881	0,833	0,586
	4M EFS 400	104,363	11,605	0,712	97,498	16,565	0,882	0,739

Table 5

R1 AT + G		Control AT		Control AT + G		p	Rotenone AT		Rotenone AT + G		p	p Control vs Rotenone
		Mean	SEM	Mean	SEM		Mean	SEM	Mean	SEM		
Duodenum	2M EFS 200	315,191	175,939	813,131	426,648	0,315	116,545	85,781	217,024	115,050	0,500	0,216
	2M EFS 400	41,398	20,512	162,965	57,991	0,076	44,004	18,337	108,959	19,453	**0,035**	0,398
	4M EFS 200	146,393	45,084	129,178	24,202	0,742	153,464	41,517	181,382	55,165	0,691	0,169
	4M EFS 400	168,833	69,876	267,884	148,191	0,555	215,126	92,574	218,659	89,956	0,979	0,25
Jejunum	2M EFS 200	118,449	33,240	227,785	52,248	0,108	72,171	42,528	118,189	49,128	0,495	0,157
	2M EFS 400	155,139	67,374	227,716	76,153	0,492	68,621	31,924	124,575	24,811	0,196	0,227
	4M EFS 200	201,091	49,656	300,511	132,088	0,491	81,788	18,877	83,871	22,815	0,945	0,083
	4M EFS 400	134,984	42,752	128,653	23,275	0,898	83,759	13,506	78,240	22,770	0,838	0,746
Ileum	2M EFS 200	341,479	280,242	565,512	454,462	0,684	122,493	46,901	82,262	44,370	0,547	0,315
	2M EFS 400	30,042	21,168	76,008	27,997	0,219	136,076	36,440	68,049	36,293	0,215	0,866
	4M EFS 200	257,809	102,348	202,297	101,587	0,706	185,114	57,208	128,629	59,287	0,503	0,125
	4M EFS 400	172,554	33,006	136,134	33,290	0,450	155,883	31,187	104,455	29,719	0,250	0,61
Colon	2M EFS 200	80,848	15,441	83,618	6,007	0,871	230,193	98,750	70,959	24,153	0,148	0,622
	2M EFS 400	82,976	15,025	76,104	3,871	0,667	122,885	16,826	72,993	5,818	**0,019**	0,666
	4M EFS 200	63,827	14,303	67,565	11,668	0,842	70,743	10,085	76,206	11,101	0,720	0,594
	4M EFS 400	63,568	14,458	70,230	9,817	0,708	70,603	9,899	77,240	10,367	0,650	0,63

Table 6

C-Off AT + G		Control AT		Control AT + G		p	Rotenone AT		Rotenone AT + G		p	p Control vs Rotenone
		Mean	SEM	Mean	SEM		Mean	SEM	Mean	SEM		
Duodenum	2M EFS 200	79,173	14,143	90,052	7,056	0,507	73,393	11,249	103,054	14,259	0,133	0,433
	2M EFS 400	95,322	20,113	95,215	14,599	0,997	90,426	18,887	88,545	11,469	0,934	0,727
	4M EFS 200	81,353	9,268	79,459	9,085	0,886	65,884	6,538	74,802	6,934	0,363	0,686
	4M EFS 400	88,081	8,791	102,368	8,660	0,266	68,931	6,566	86,449	8,614	0,125	0,214
Jejunum	2M EFS 200	95,973	15,359	91,761	12,065	0,833	78,510	32,023	100,143	25,408	0,608	0,772
	2M EFS 400	98,213	15,010	97,778	8,622	0,981	75,032	14,189	82,999	9,473	0,651	0,275
	4M EFS 200	68,503	10,650	78,574	8,404	0,569	79,165	10,480	95,389	9,728	0,273	0,209
	4M EFS 400	74,035	7,839	90,882	16,642	0,274	82,448	10,150	98,868	8,893	0,241	0,612
Ileum	2M EFS 200	73,371	25,338	84,767	13,115	0,698	65,949	12,992	97,729	14,628	0,135	0,524
	2M EFS 400	80,838	20,061	91,997	12,890	0,650	76,370	10,713	105,610	12,336	0,104	0,463
	4M EFS 200	81,073	21,466	106,900	20,971	0,404	59,825	6,443	121,527	17,834	**0,004**	0,601
	4M EFS 400	76,077	14,726	107,241	15,902	0,172	63,085	6,077	116,604	24,860	**0,004**	0,673
Colon	2M EFS 200	99,826	36,553	190,669	32,531	0,093	27,022	11,878	234,174	39,160	**<0,001**	0,413
	2M EFS 400	94,823	47,659	564,570	365,080	0,231	-16,350	27,606	1484,669	1165,451	0,227	0,468
	4M EFS 200	109,166	16,862	145,785	16,408	0,139	97,017	13,881	110,867	14,259	0,496	0,128
	4M EFS 400	104,363	11,605	132,025	14,554	0,157	97,498	16,565	120,712	17,657	0,352	0,628

Table 7

Serotonin max	Control		Rotenone	
	Mean	SEM	Mean	SEM
Duodenum	1,395	0,13	1,474	0,127
Jejunum	1,209	0,046	1,205	0,099
Ileum	1,009	0,031	0,924	0,035
Colon	0,877	0,055	0,851	0,042

p	Jejunum	Ileum	Colon
Duodenum	0,300	**0,006**	**<0,001**
Jejunum		0,240	**0,013**
Ileum			0,594

Table 8

Serotonin min	Control		Rotenone	
	Mean	SEM	Mean	SEM
Duodenum	1,202	0,086	1,249	0,080
Jejunum	1,118	0,066	1,068	0,029
Ileum	0,933	0,033	0,914	0,055
Colon	1,030	0,085	0,972	0,026

p	Jejunum	Ileum	Colon
Duodenum	0,840	**0,069**	0,342
Jejunum		0,284	0,807
Ileum			0,778

Table 9

M3-Receptor		Control		Rotenone		p
		Mean	SEM	Mean	SEM	
Duodenum	2M	1	0,068	1,187	0,091	**<0,01**
	4M	1	0,040	1,163	0,025	**<0,01**
Jejunum	2M	1	0,033	1,224	0,046	**<0,01**
	4M	1	0,033	1,151	0,068	**<0,05**
Ileum	2M	1	0,057	1,229	0,059	**<0,05**
	4M	1	0,062	1,223	0,035	**<0,05**
Colon	2M	1	0,042	1,166	0,456	**<0,05**
	4M	1	0,061	1,305	0,060	**<0,01**

Table 10

ß2-subunit		Control		Rotenone		p
		Mean	SEM	Mean	SEM	
Duodenum	2M	1	0,105	1,260	0,038	**<0,05**
	4M	1	0,012	1,425	0,104	**<0,01**
Jejunum	2M	1	0,065	1,174	0,059	>0,05
	4M	1	0,033	1,809	0,240	**<0,05**
Ileum	2M	1	0,040	1,181	0,091	>0,05
	4M	1	0,031	1,212	0,040	**<0,01**
Colon	2M	1	0,012	1,287	0,067	**<0,01**
	4M	1	0,084	1,570	0,122	**<0,01**

Table 11

D2-Receptor		Control		Rotenone		p
		Mean	SEM	Mean	SEM	
Duodenum	2M	1	0,044	1,065	0,066	>0,05
	4M	1	0,190	1,653	0,189	**<0,05**
Jejunum	2M	1	0,060	1,283	0,100	**<0,05**
	4M	1	0,150	1,179	0,080	>0,05
Ileum	2M	1	0,005	1,151	0,078	**<0,01**
	4M	1	0,047	1,153	0,060	>0,05
Colon	2M	1	0,064	1,514	0,016	**<0,05**
	4M	1	0,046	1,205	0,029	**<0,01**

Table 12

PGP9.5		Control		Rotenone		p
		Mean	SEM	Mean	SEM	
Duodenum	2M	1	0,17	0,60	0,09	>0,05
	4M	1	0,08	0,77	0,11	>0,05
Jejunum	2M	1	0,15	0,76	0,15	>0,05
	4M	1	0,03	1,01	0,28	>0,05
Ileum	2M	1	0,02	1,02	0,03	>0,05
Colon	2M	1	0,16	1,26	0,06	>0,05
	4M	1	0,31	1,01	0,11	>0,05

Table 13

ChAT		Control		Rotenone		p
		Mean	SEM	Mean	SEM	
Duodenum	2M	1	0,11	0,84	0,16	>0,05
	4M	1	0,17	0,58	0,09	>0,05
Jejunum	2M	1	0,10	0,90	0,10	>0,05
	4M	1	0,26	0,58	0,07	>0,05
Colon	2M	1	0,15	0,57	0,06	**<0,05**
	4M	1	0,26	2,00	0,04	>0,05
All regions	2M	1	0,06	0,77	0,06	**<0,05**
	4M	1	0,08	0,75	0,06	**<0,05**

Table 14

TH		Control		Rotenone		p
		Mean	SEM	Mean	SEM	
Duodenum	2M	1	0,07	1,71	0,15	**<0,01**
	4M	1	0,17	0,78	0,19	>0,05
Jejunum	2M	1	0,05	0,66	0,13	**<0,05**
	4M	1	0,23	0,98	0,24	>0,05
Ileum	2M	1	0,09	1,07	0,23	>0,05
Colon	2M	1	0,10	1,48	0,14	**<0,05**
	4M	1	0,24	1,29	0,16	>0,05
All regions	2M	1	0,06	0,78	0,06	>0,05
	4M	1	0,08	0,75	0,06	>0,05

Table 1 Statistical results comparing the reaction to carbachol between vehicle and rotenone-exposed mice. A 2-way ANOVA was used to compare the effect of carbachol on the amplitude, the maximal exerted contraction force and the muscle tone between vehicle and rotenone-exposed mice. The level of significance is shown as the p value.

Table 2 Statistical results comparing the reaction to dopamine between vehicle and rotenone-exposed mice. A 2-way ANOVA was used to compare the effect of dopamine on the amplitude, the maximal exerted contraction force and the muscle tone between vehicle and rotenone-exposed mice. The level of significance is shown as the p value.

Table 3 shows the mean values (mean), the standard error of the mean (SEM) and the significance (p) of the comparison (unpaired Student´s t-test) between the relaxation observed in R1 induced by the EFS (200 and 400 m µs) in the absence of any blocker (consider as 100%) and the relaxation obtained in the presence of atropine (AT) normalized to the value without substance and shown as percentage. The far-right column shows the significance when comparing the results obtained in the presence of AT between control and rotenone exposed mice.

Table 4 shows the mean values (mean), the standard error of the mean (SEM) and the significance (p) of the comparison (unpaired Student´s t-test) between the contraction observed in C-Off induced by the EFS (200 and 400 µs) in the absence of any blocker (consider as 100%) and the contraction obtained in the presence of atropine (AT). The far-right column shows the significance when comparing the results obtained in the presence of AT between control and rotenone exposed mice.

Table 5 shows the mean values (mean), the standard error of the mean (SEM) and the significance (p) of the comparison (unpaired Student´s t-test) between the relaxation (R1) obtained with the EFS (200 and 400 µs) in the presence of atropine (AT) and in the presence of AT and guanethidine (G). The absence of any blocker is considered as 100% and all other values were normalized to that value. The far-right column shows the significance when comparing the results obtained in the presence of AT and G between control and rotenone exposed mice.

Table 6 shows the mean values (mean), the standard error of the mean (SEM) and the significance (p) of the comparison (unpaired Student´s t-test) between the contraction (C-Off) obtained with the EFS (200 and 400 µs) in the presence of atropine (AT) and in the presence of AT and guanethidine (G). The absence of any blocker is considered as 100% and all other values were normalized to that value.

The far-right column shows the significance when comparing the results obtained in the presence of AT and G between control and rotenone exposed mice.

Table 7 shows the mean values (mean) and the standard error of the mean (SEM) for maximal tension exerted by serotonin (S) when added in the presence of AT and G. The *P*-value table shows the significance of the difference in the effect that S had between regions of the intestine.

Table 8 shows the mean values (mean) and the standard error of the mean (SEM) for the muscle tone (Min.) by serotonin (S) when added in the presence of AT and G. The *P*-value table shows the significance of the difference in the effect that S had between regions of the intestine.

Table 9 shows the mean values (mean), the standard error of the mean (SEM) and the significance (p) of the difference between control and rotenone exposed mice in the expression of the muscarinic cholinergic Receptor (M3) normalized to the total amount of neurons (PGP9.5) and to the mean of these values in the control group.

Table 10 shows the mean values (mean), the standard error of the mean (SEM) and the significance (p) of the difference between control and rotenone exposed mice in the expression of the adrenergic Receptor (ß2-subunit) normalized to the total amount of neurons (PGP9.5) and to the mean of these values in the control group.

Table 11 shows the mean values (mean), the standard error of the mean (SEM) and the significance (p) of the difference between control and rotenone exposed mice in the expression of the dopaminergic Receptor (D2) normalized to the total amount of neurons (PGP9.5) and to the mean of these values in the control group.

Table 12 shows the mean values (mean), the standard error of the mean (SEM) and the significance (p) of the difference between control and rotenone exposed mice in the expression of PGP 9.5 normalized to the mean of the control group.

Table 13 shows the mean values (mean), the standard error of the mean (SEM) and the significance (p) of the difference between control and rotenone exposed mice in the expression of choline acetyltransferase (ChAT) normalized to the total amount of neurons (PGP9.5) and to the mean of these values in the control group.

Table 14 shows the mean values (mean), the standard error of the mean (SEM) and the significance (p) of the difference between control and rotenone exposed mice in the expression of tyrosine hydroxylase (TH) normalized to the total amount of neurons (PGP9.5) and to the mean of these values in the control group.
