# Supplementary material for: Pathophysiological Changes in the Enteric Nervous System of Rotenone-Exposed Mice as Early Radiological Markers for Parkinson's Disease
Source: Front Neurol. 2021 Mar 22;12:642604. doi: 10.3389/fneur.2021.642604 (PMC8030242; doi:10.3389/fneur.2021.642604)

PGP9.5 Duodenum 2M

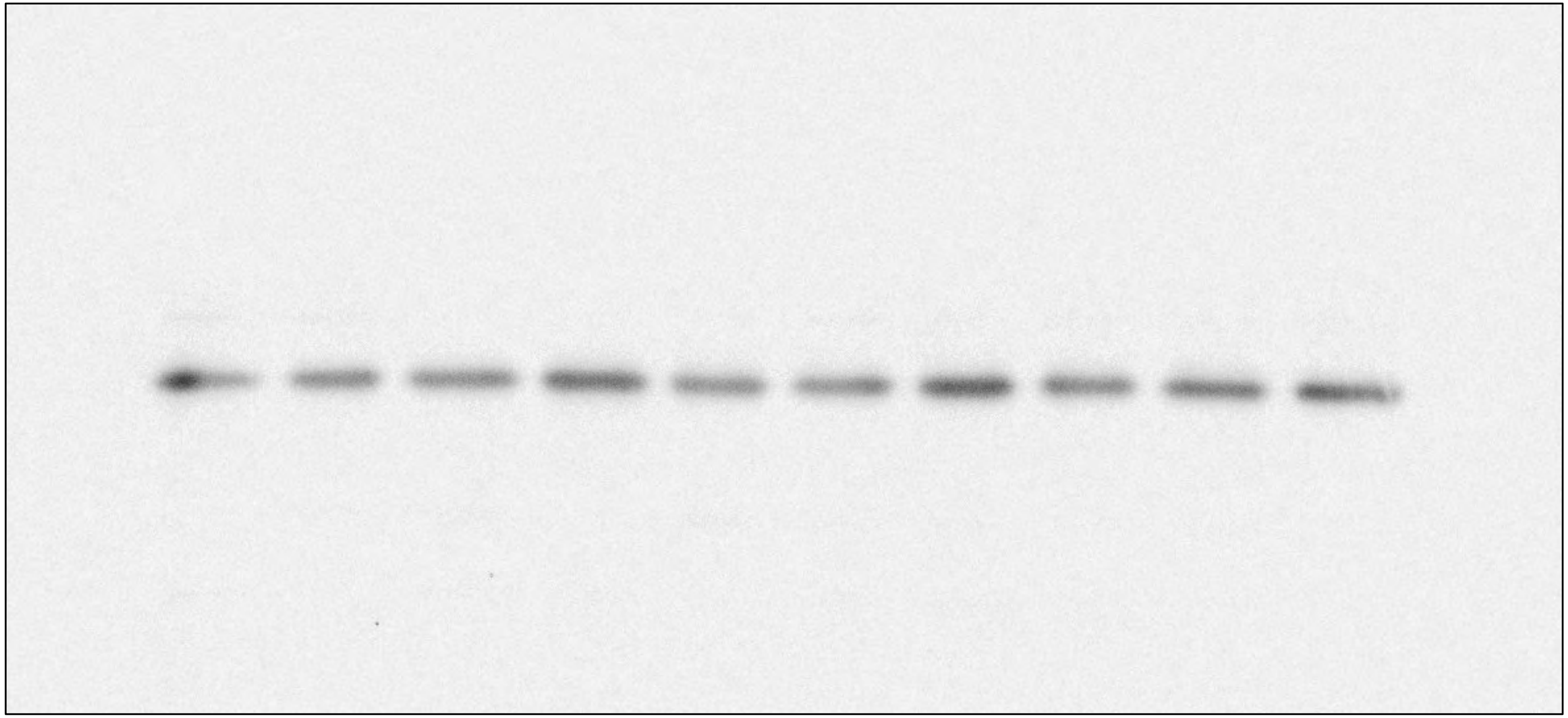

GAPDH Duodenum 2M

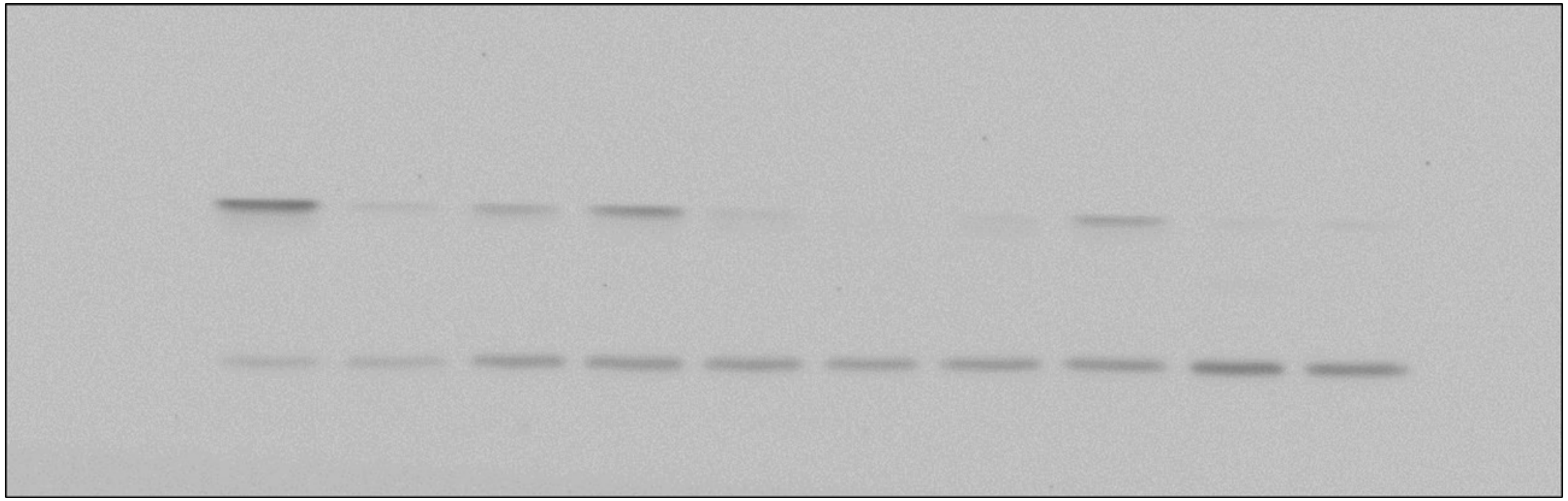

TH Duodenum 2M

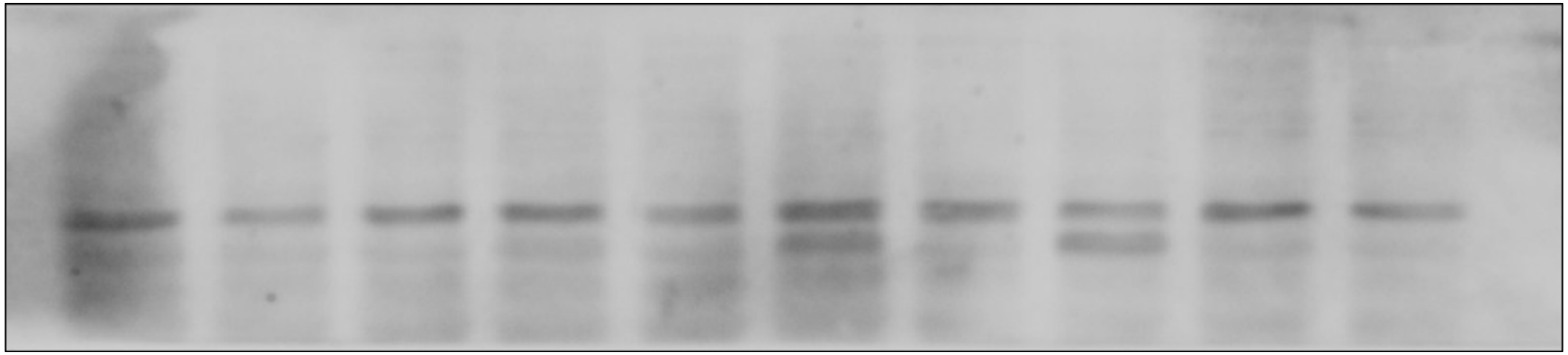

ChAT Duodenum 2M

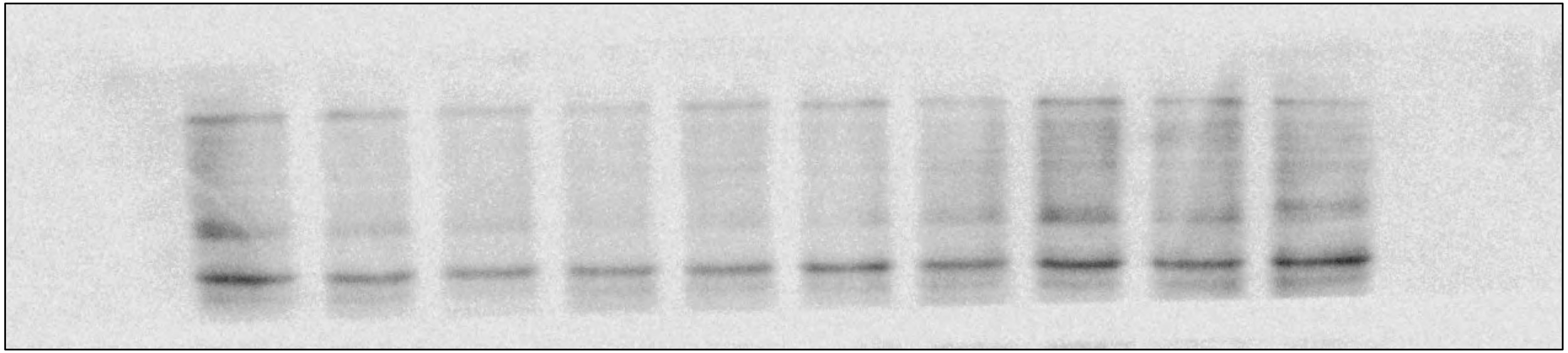

PGP9.5 Duodenum 4M

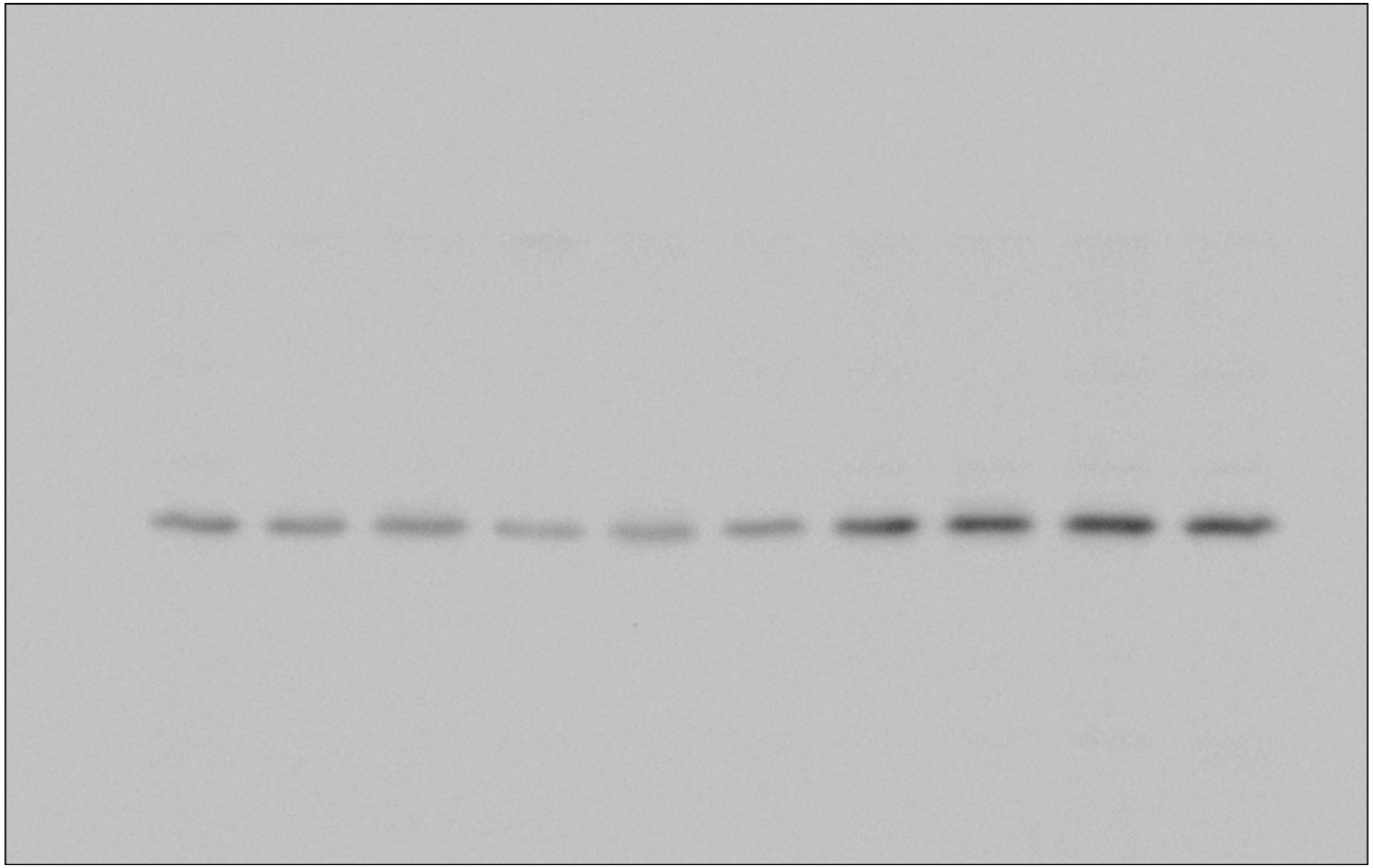

GAPDH Duodenum 4M

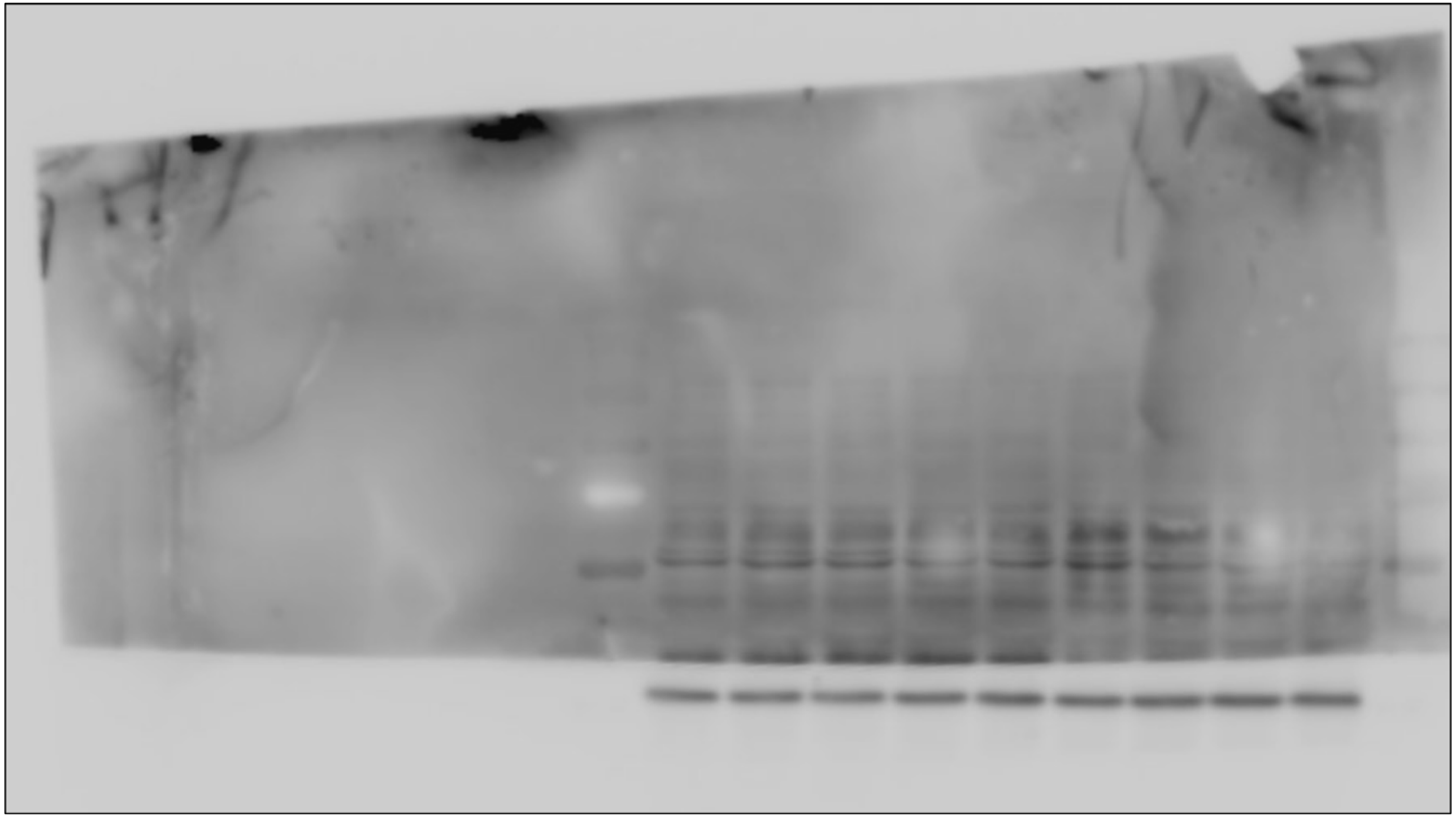

TH Duodenum 4M

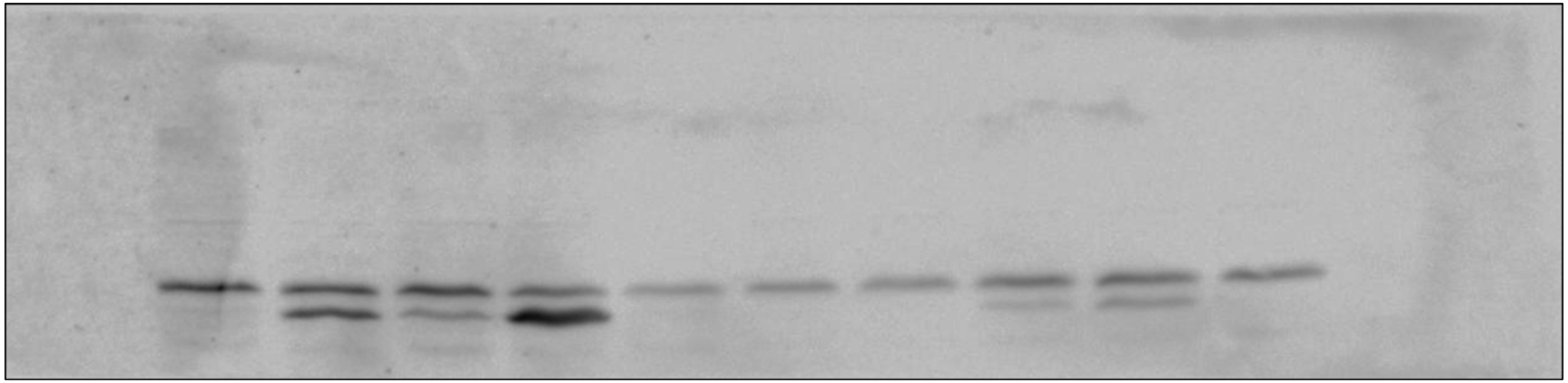

# ChAT Duodenum 4M

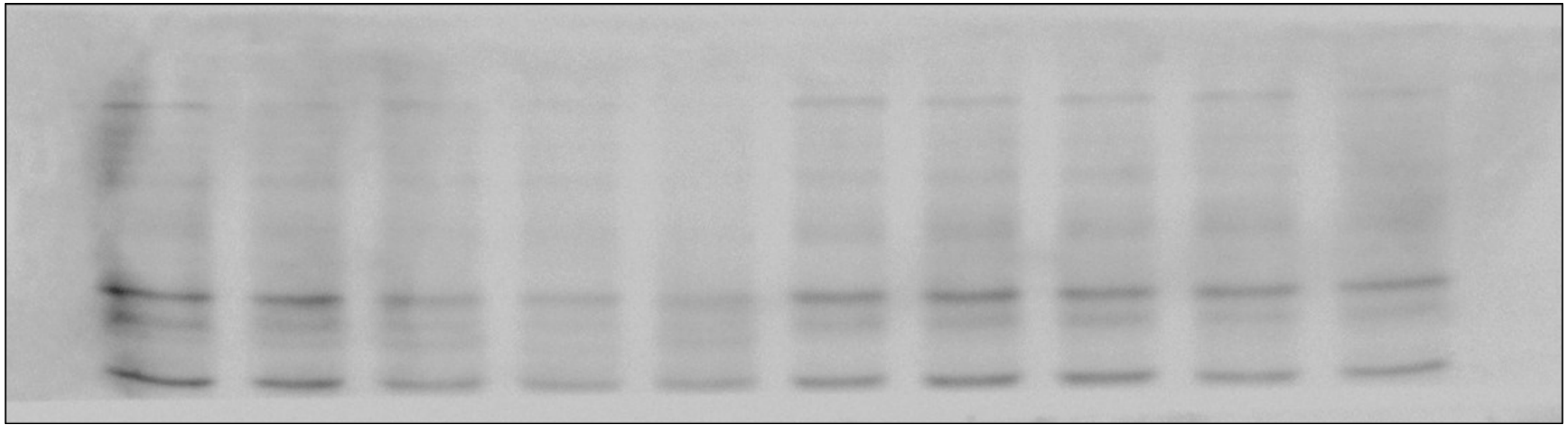

PGP9.5 Jejunum 2M

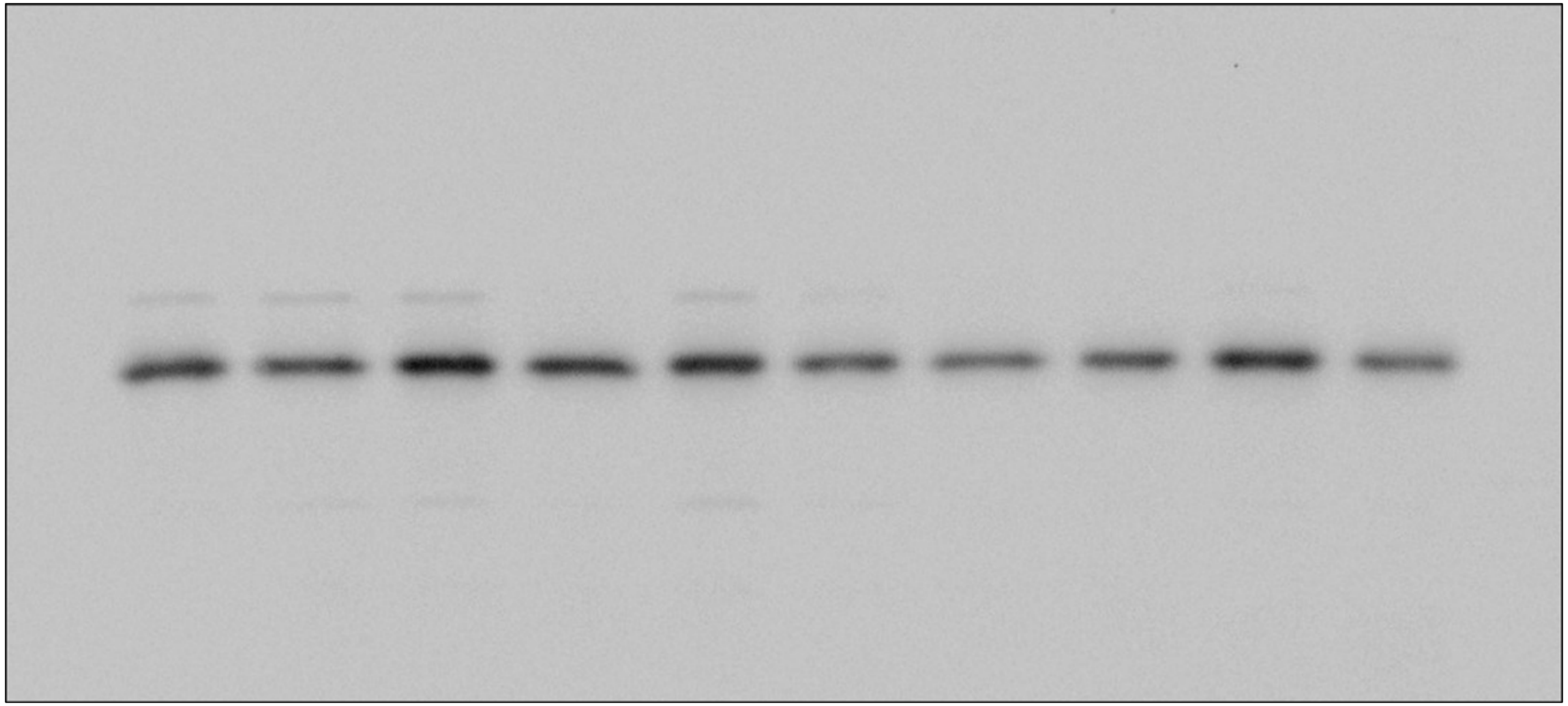

GAPDH Jejunum 2M

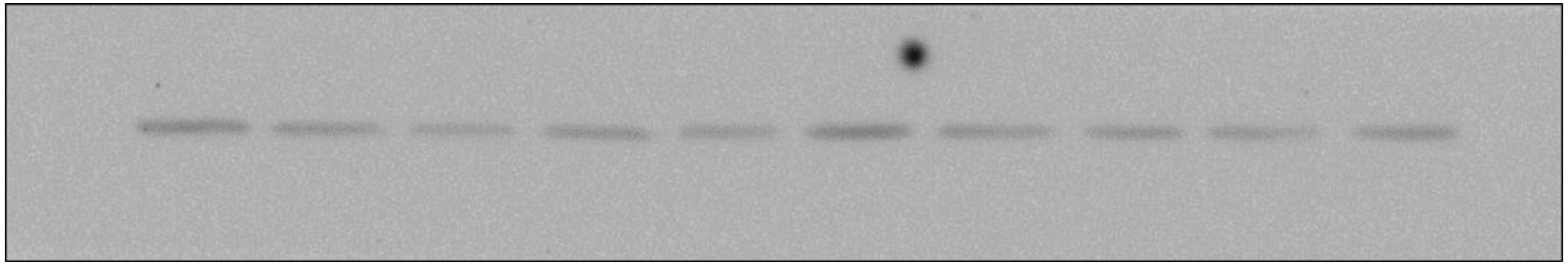

TH Jejunum 2M

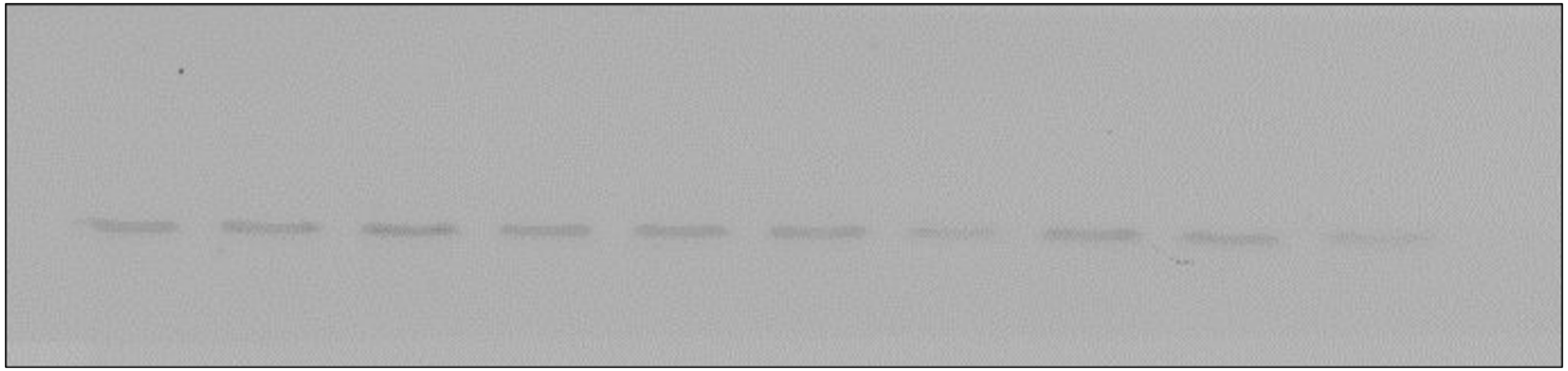

ChAT Jejunum 2M

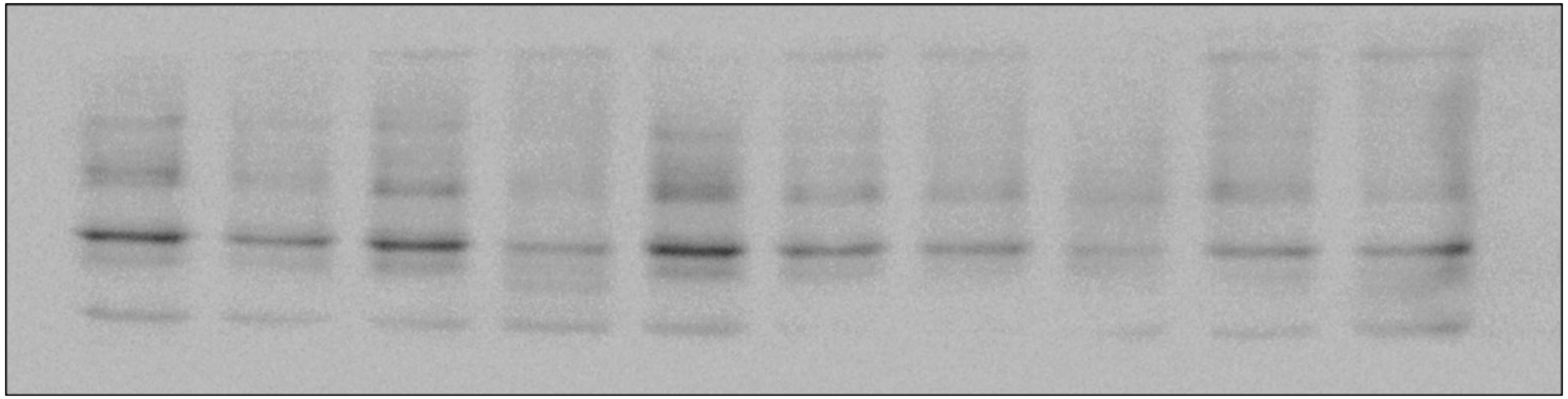

PGP9.5 Jejunum 4M

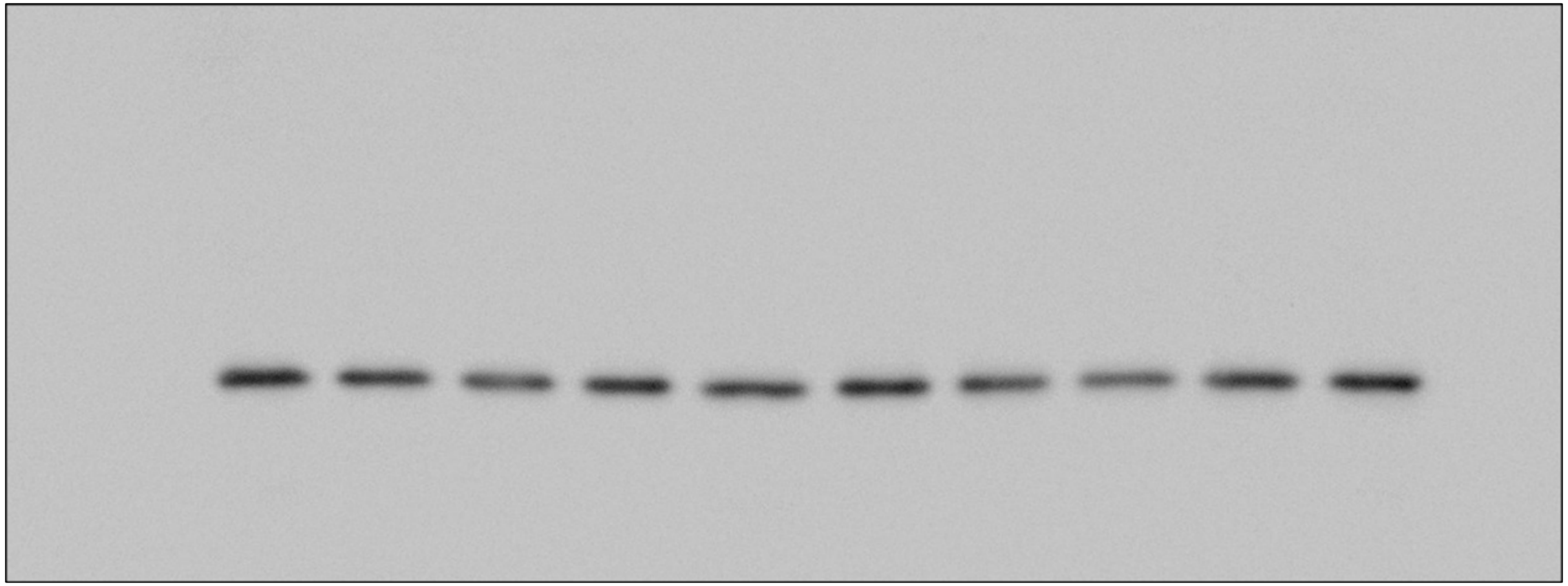

GAPDH Jejunum 4M

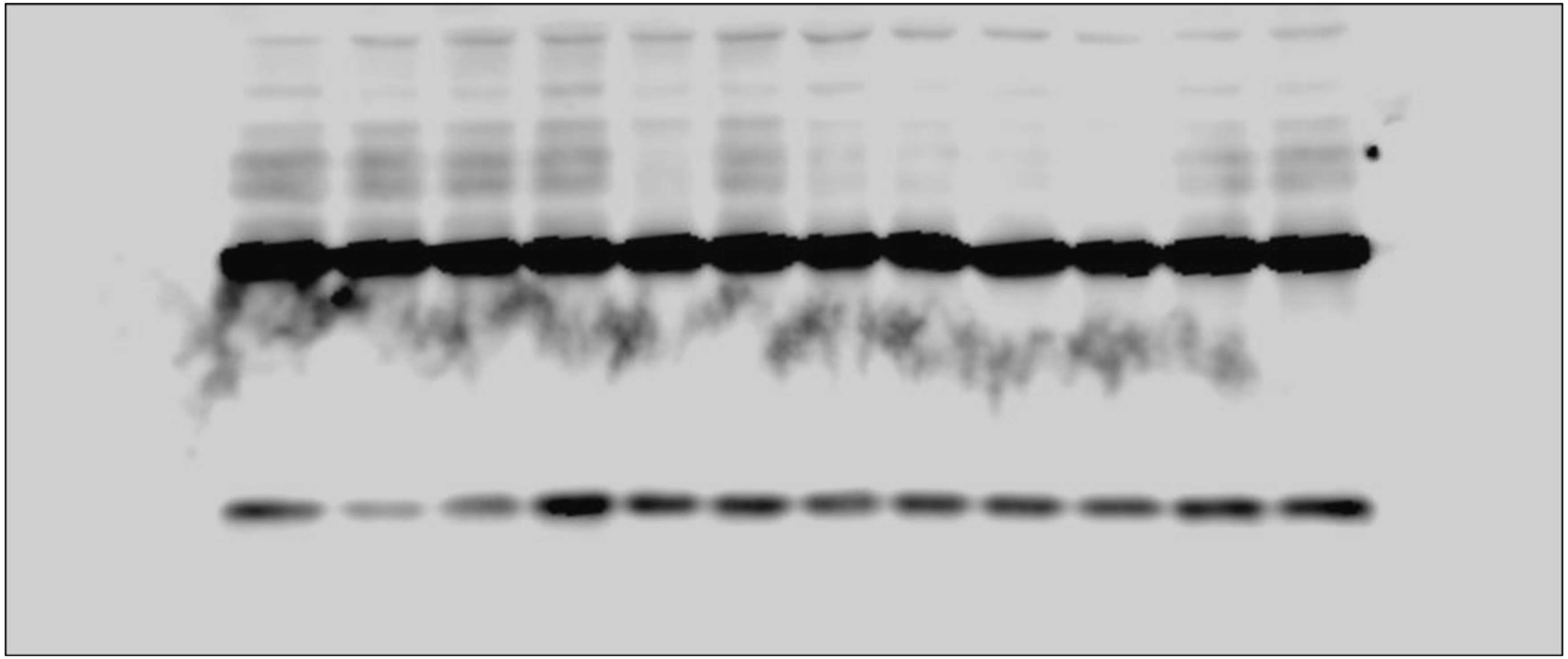

TH Jejunum 4M

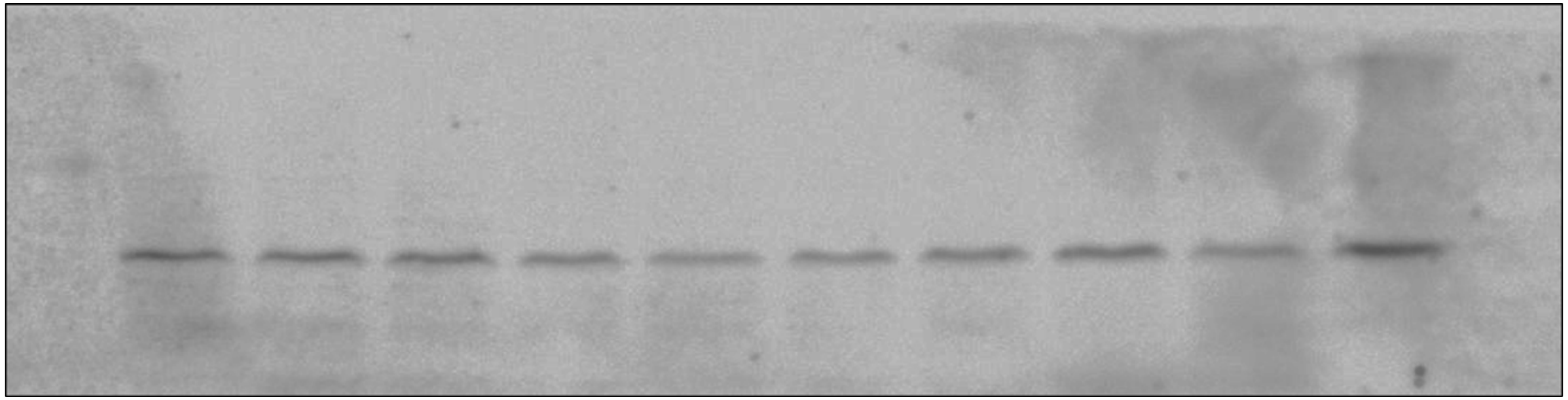

ChAT Jejunum 4M

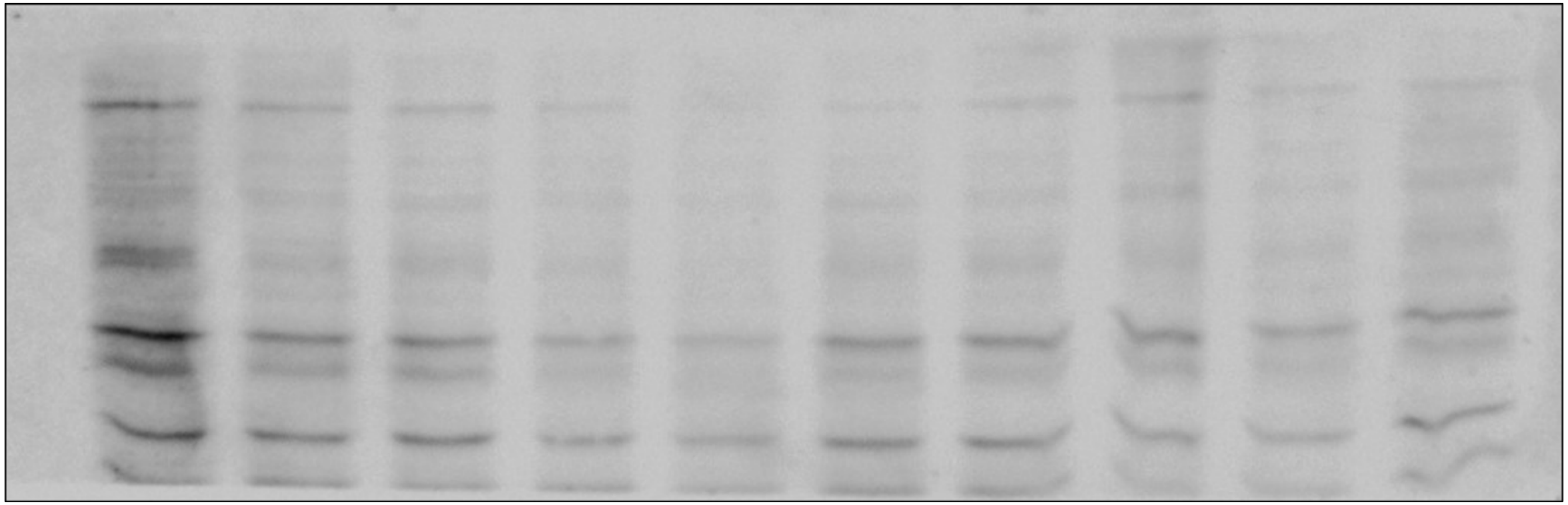

PGP9.5 Ileum 2M

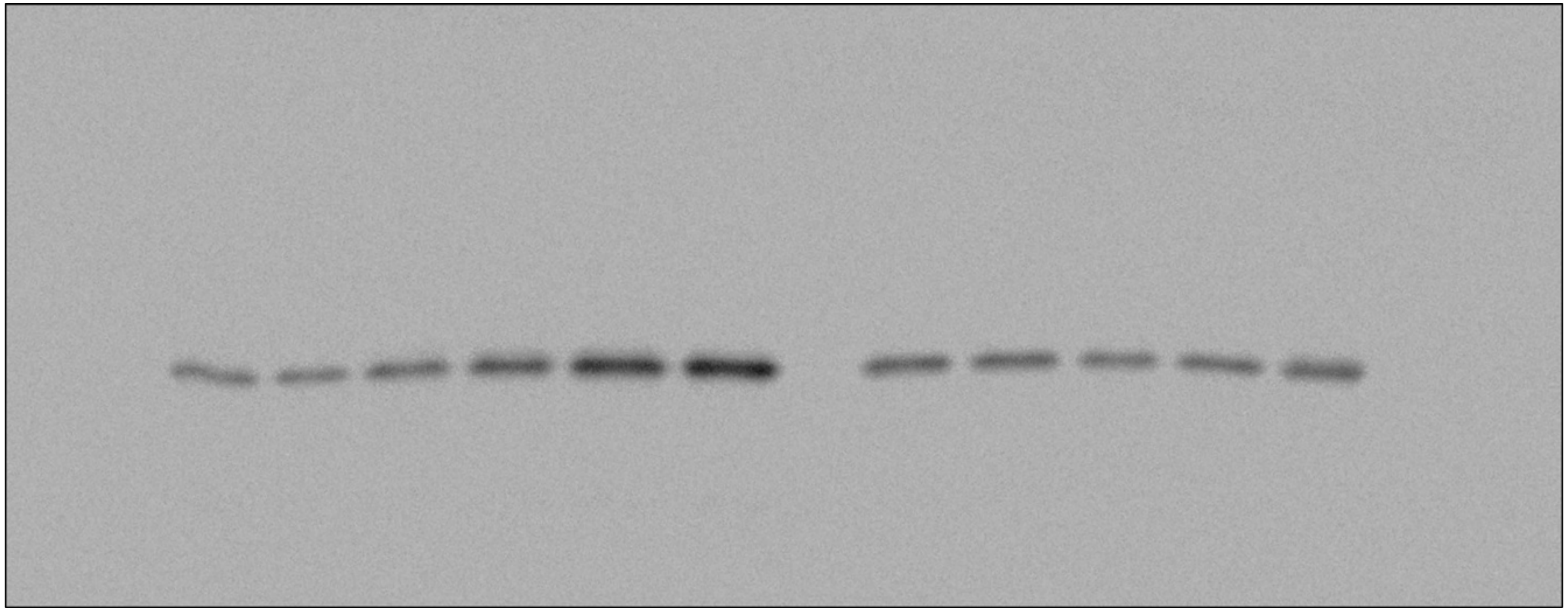

GAPDH Ileum 2M

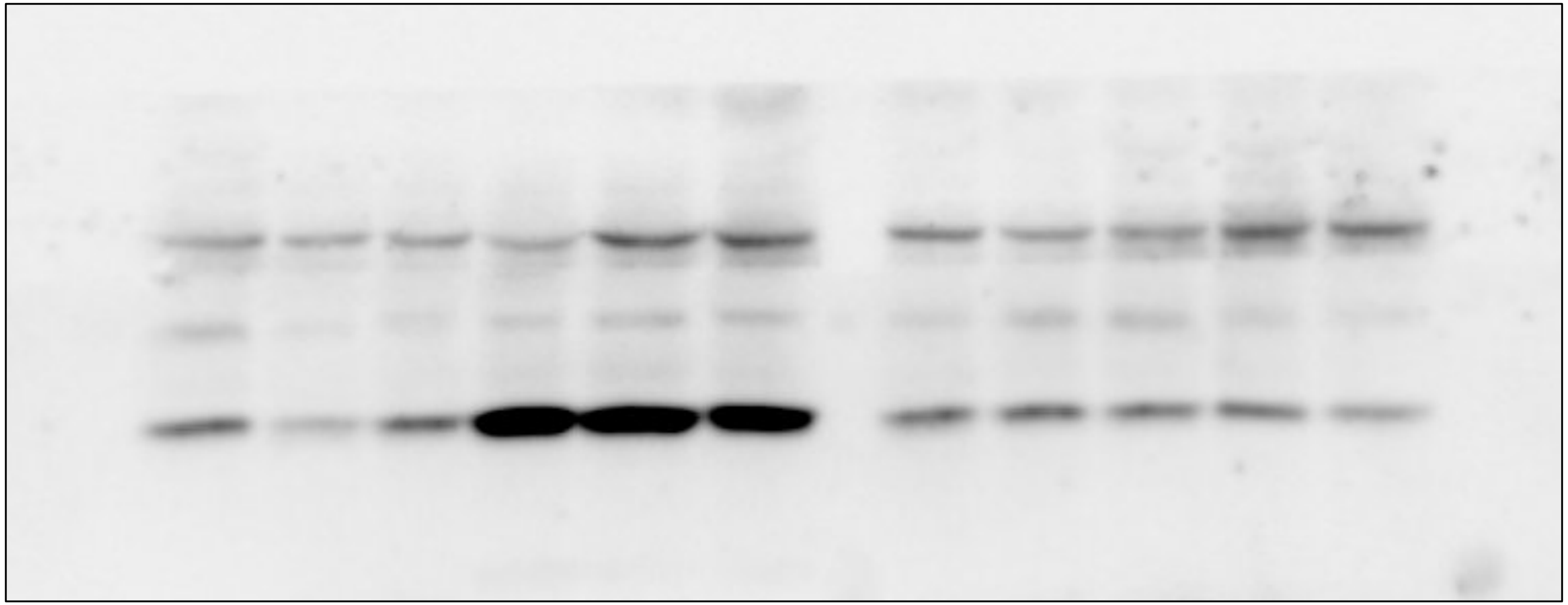

TH Ileum 2M

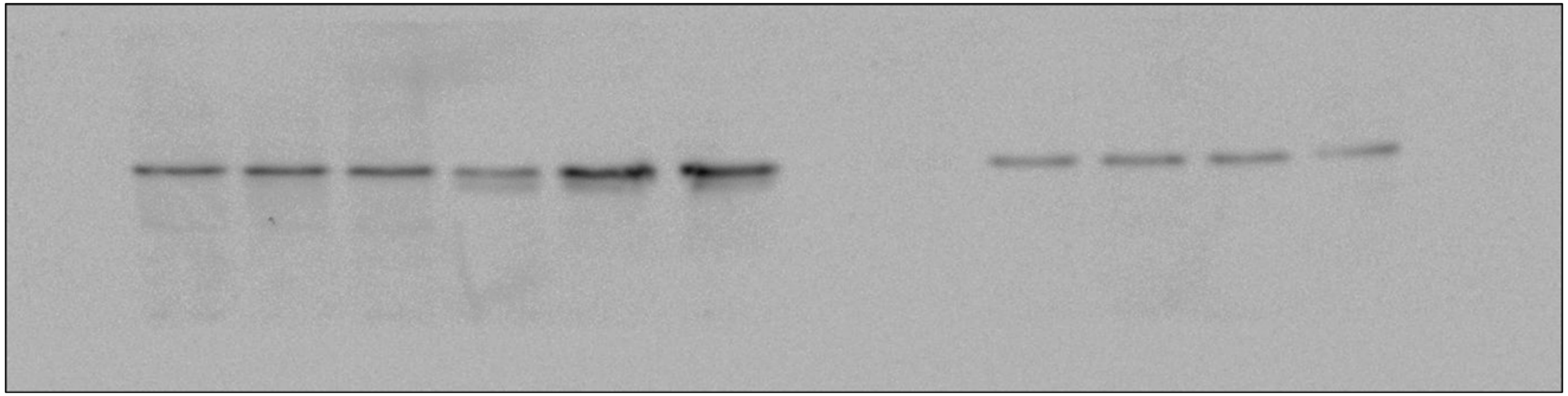

ChAT Ileum 2M

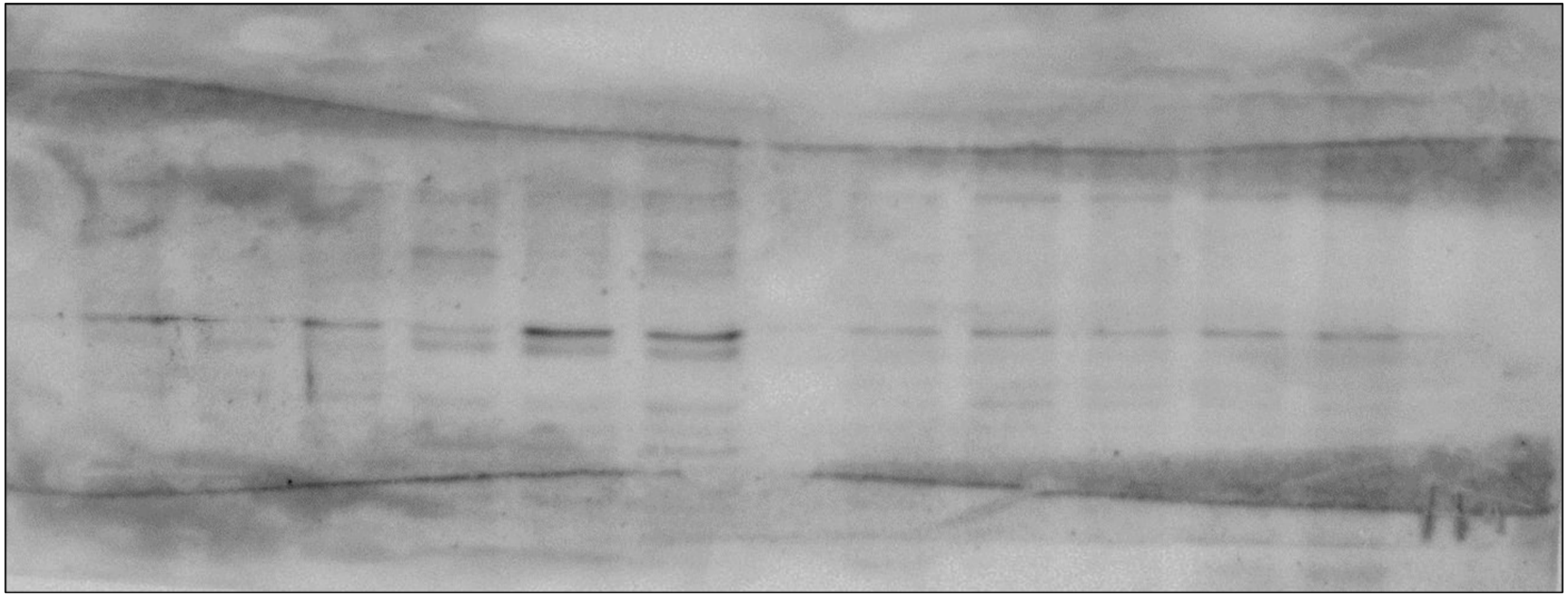

PGP9.5 Ileum 4M

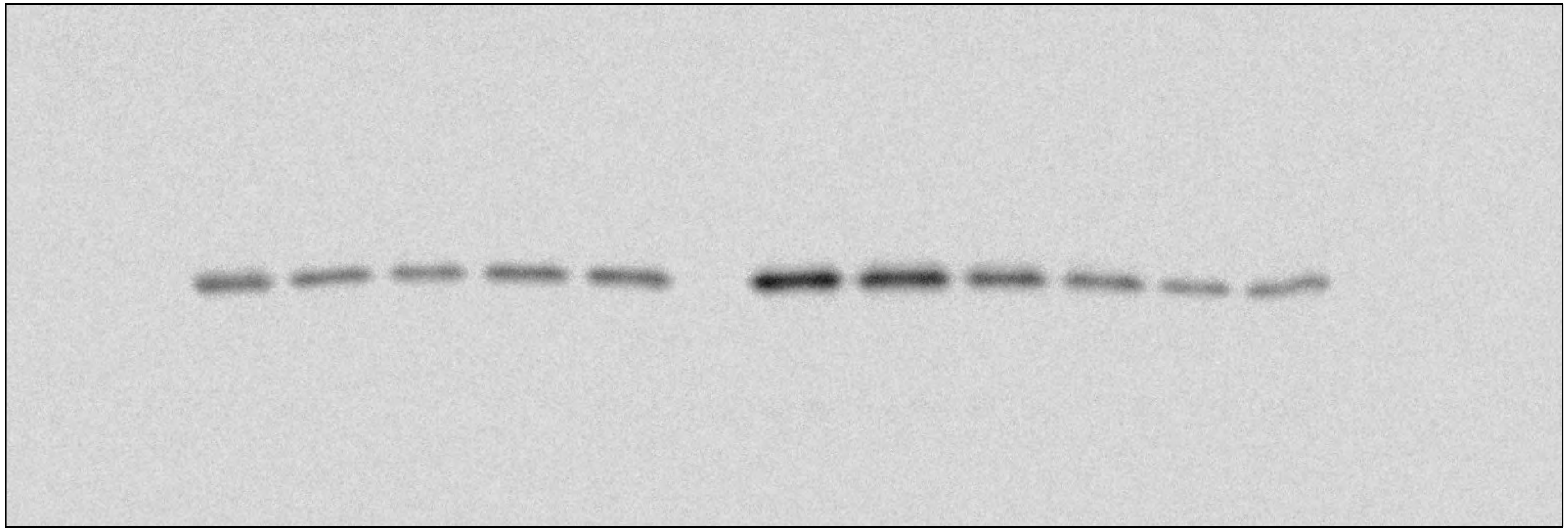

GAPDH Ileum 4M

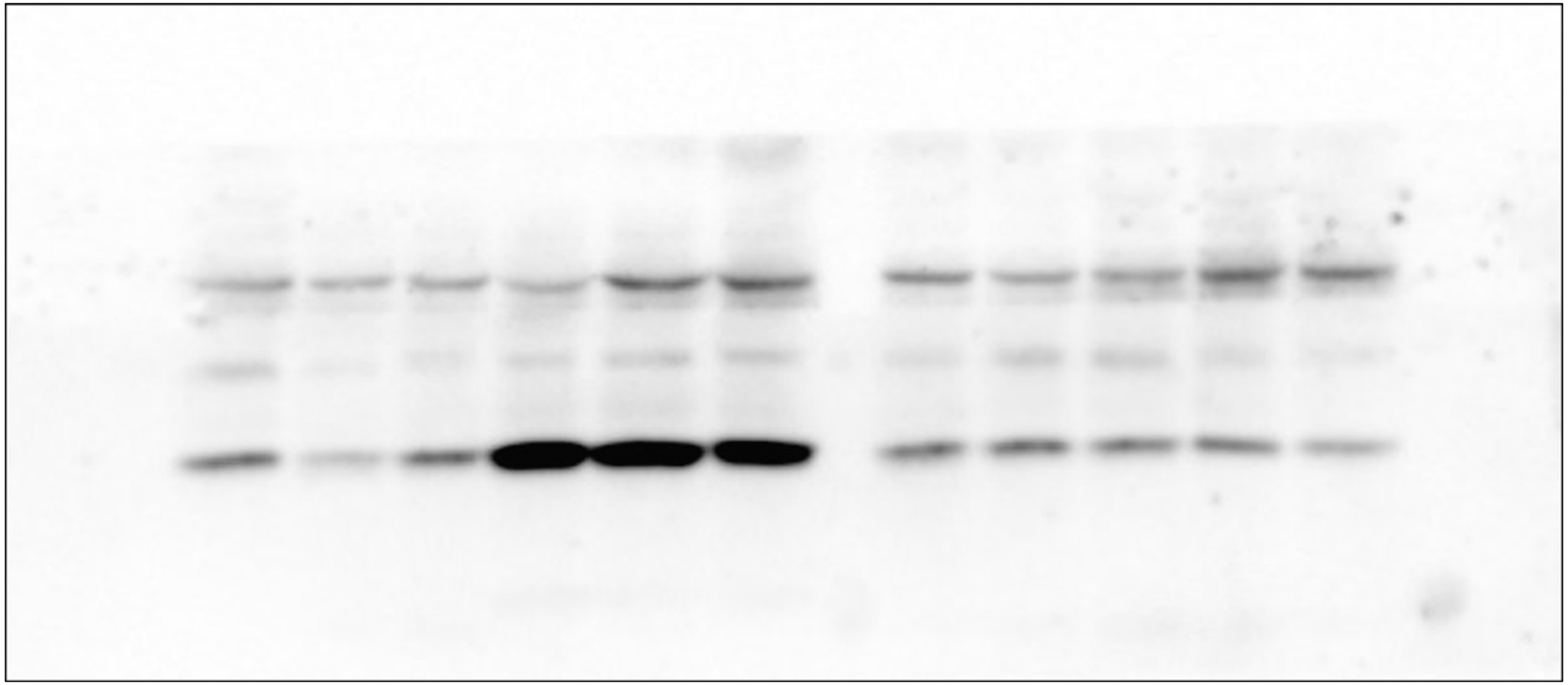

TH Ileum 4M

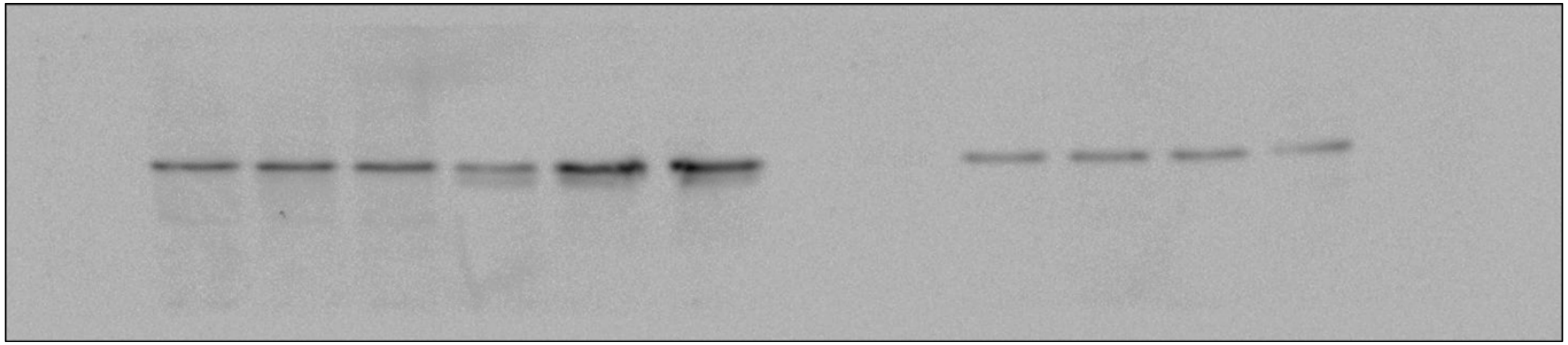

ChAT Ileum 4M

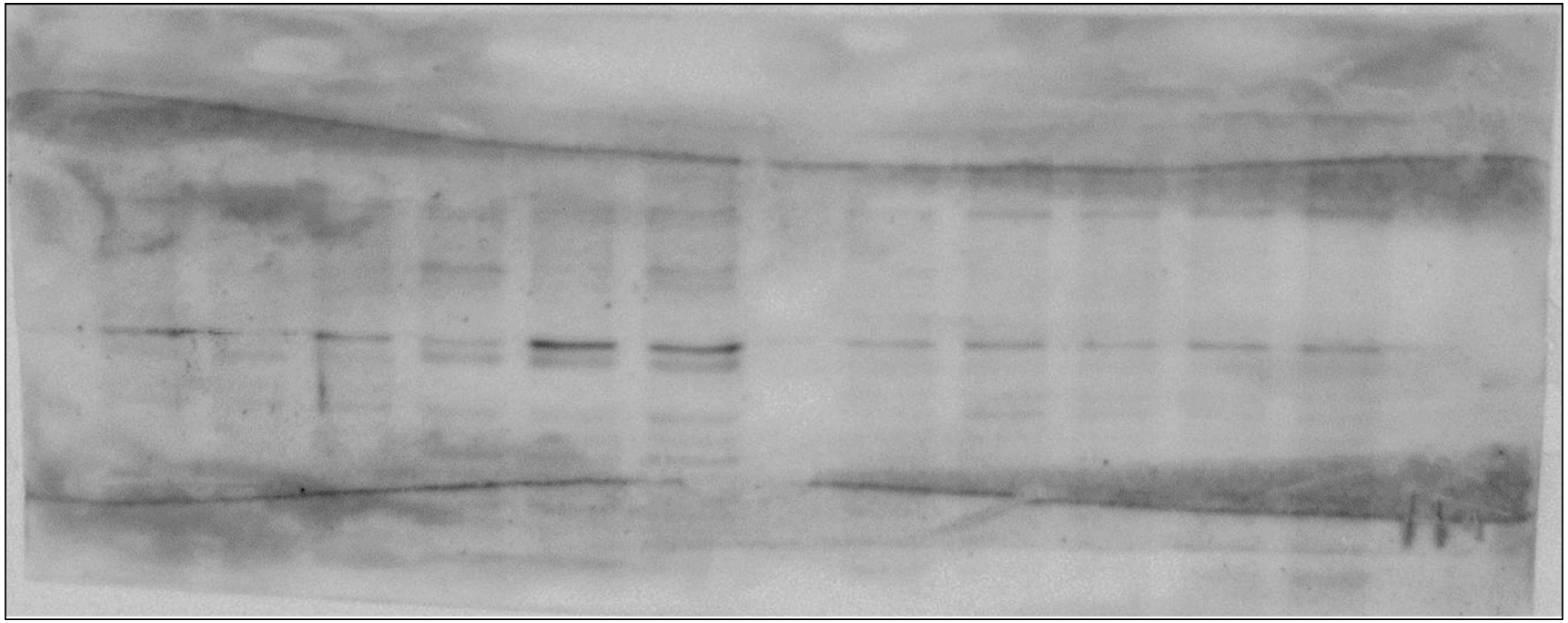

PGP9.5 Colon 2M

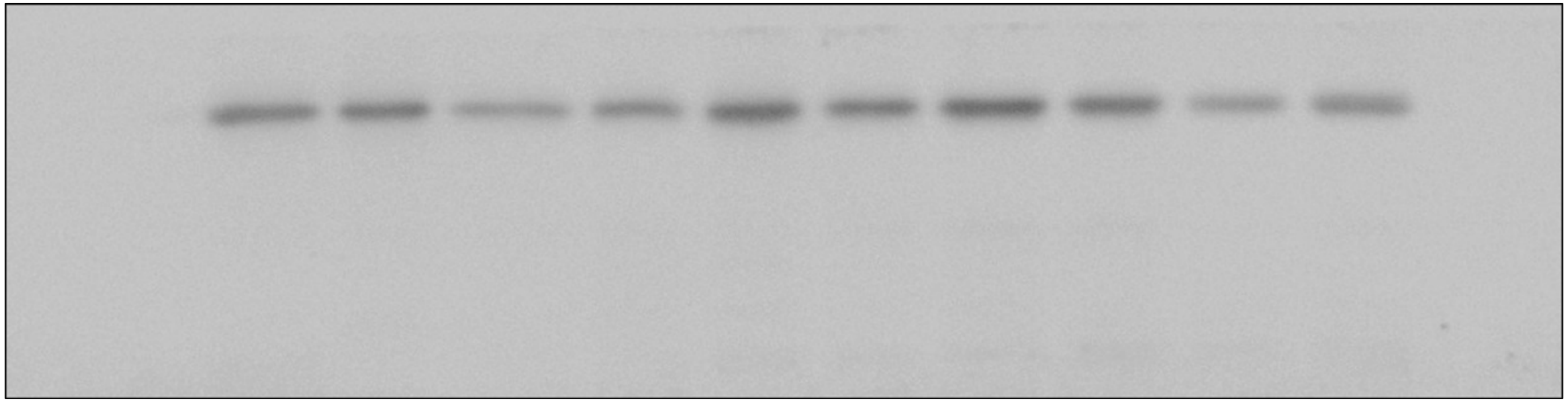

GAPDH Colon 2M

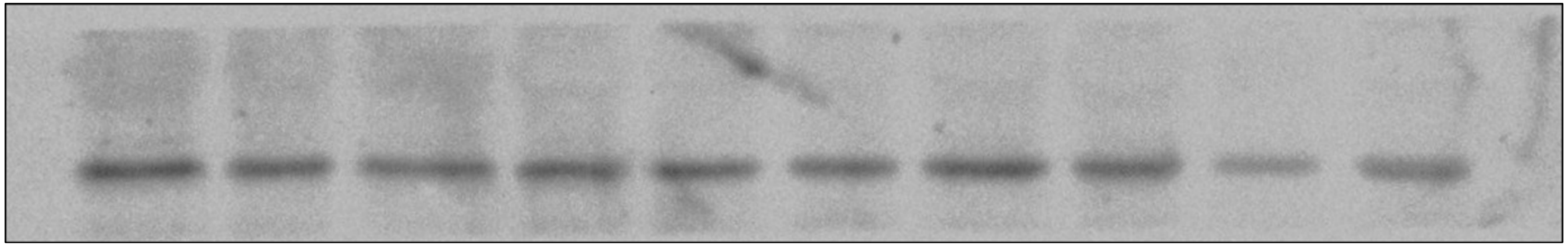

TH Colon 2M

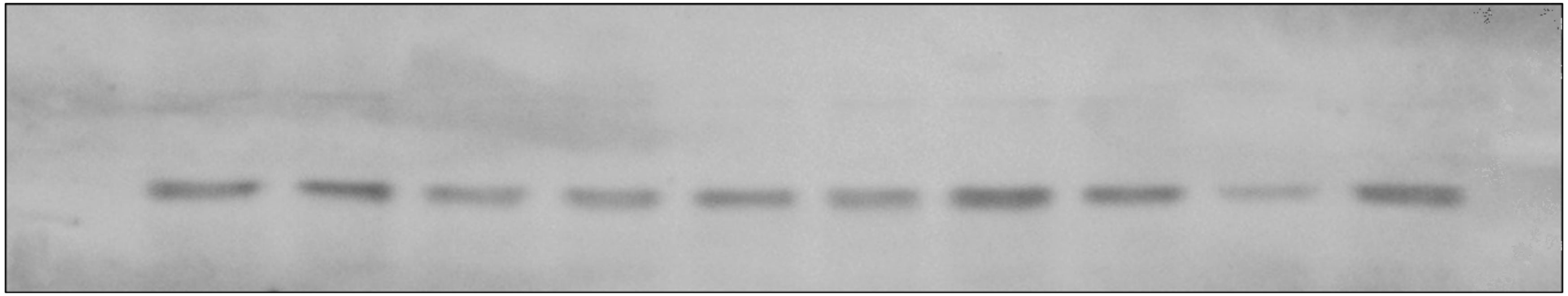

ChAT Colon 2M

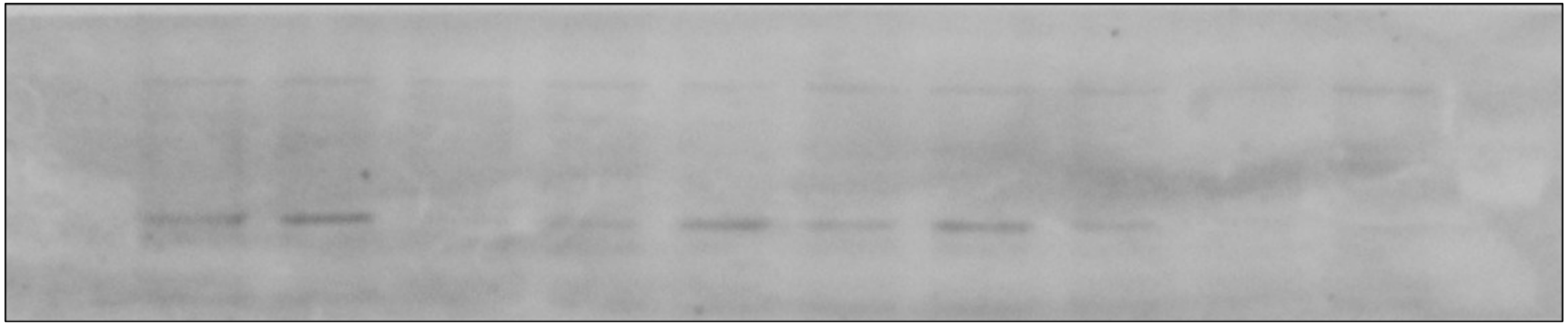

PGP9.5 Colon 4M

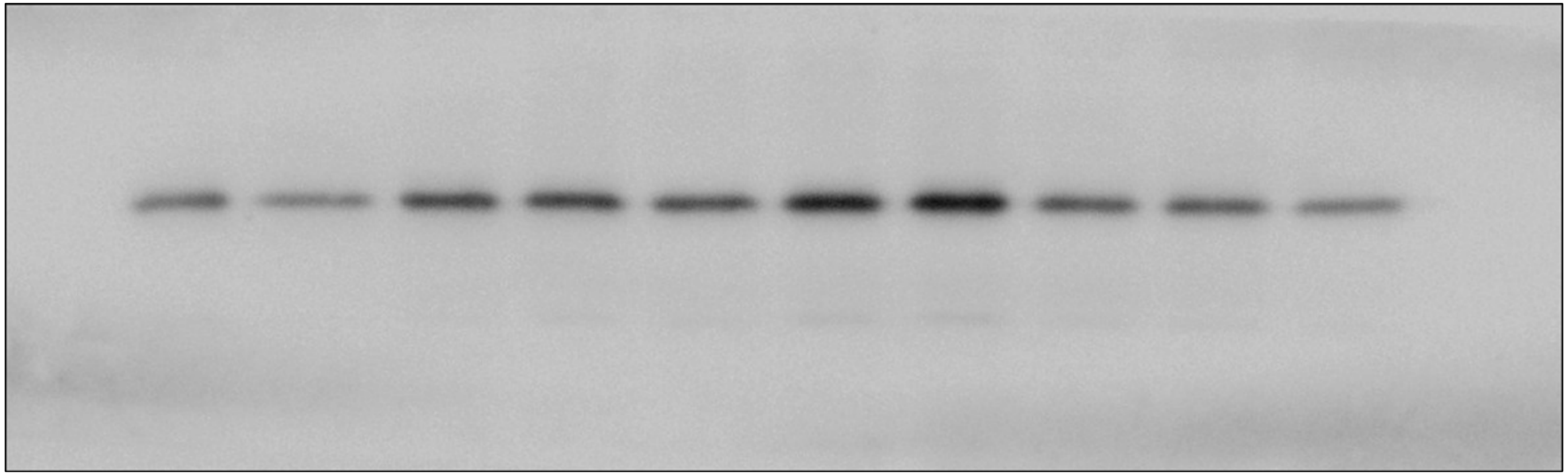

GAPDH Colon 4M

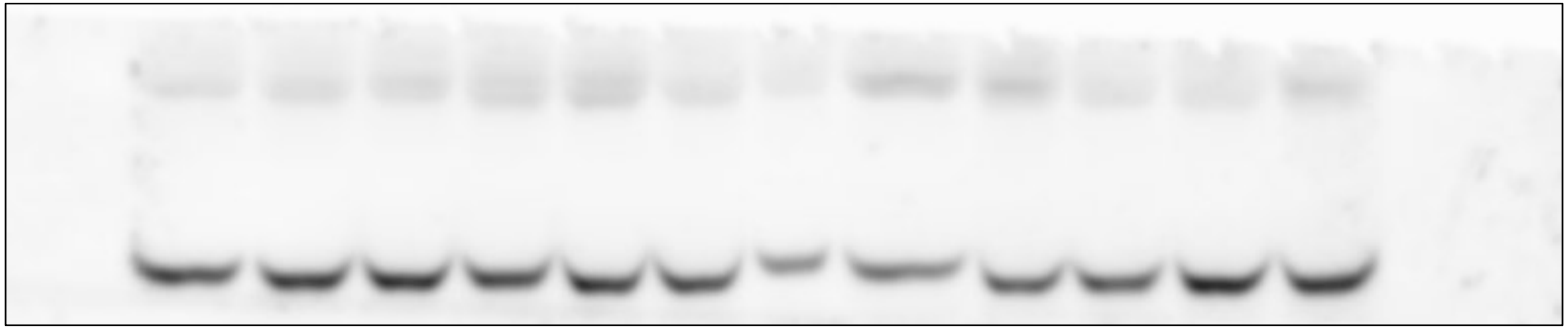

TH Colon 4M

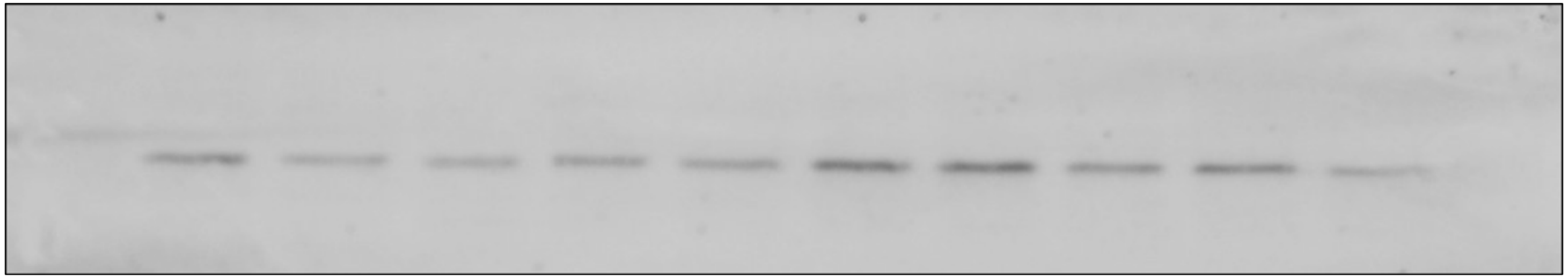

ChAT Colon 4M

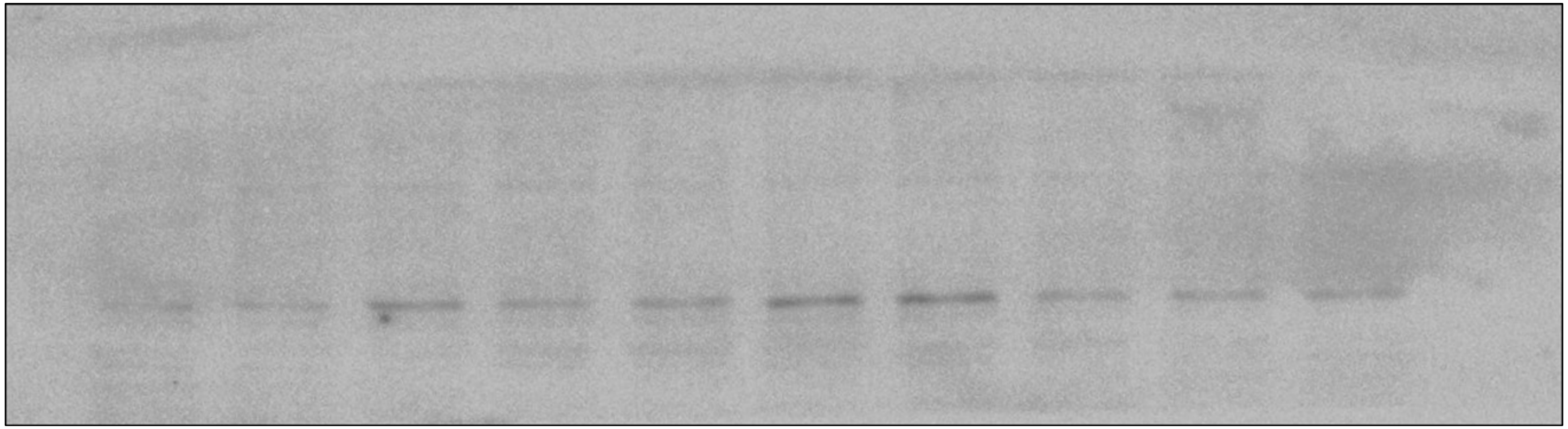

Supplement: Supplementary file 2 [file Data_Sheet_2.PDF]
