# Supplementary material for: Pathophysiological Changes in the Enteric Nervous System of Rotenone-Exposed Mice as Early Radiological Markers for Parkinson's Disease
Source: Front Neurol. 2021 Mar 22;12:642604. doi: 10.3389/fneur.2021.642604 (PMC8030242; doi:10.3389/fneur.2021.642604)
Supplement: Supplementary file 3 [file Table_1.DOCX]

function paco5

% letzte ─nderung: K.B. 10.01.2017

close all

clear

global setup ftsize b a b2 a2 erg

% Think pad : FontSize',12,

% GNR6PN: FontSize',10, screen size: 1 1 1024 768

ftsize = 12;

scrsz = get(0,'ScreenSize');

f = figure('Position',[1,20,scrsz(3), scrsz(4)*0.96],'Toolbar','none',...

'MenuBar','none');

setup.ErgFilename = 'PacoErgebnis.txt'; % Hier den Dateinamen Σndern auf dem die Ergebnisse gespeichert weren (.txt) !

setup.ErgFilepath = 'D:\A-Data\Paco\Auswertung'; % Hier den Ordner Σndern, in dem die Ergebnis-Datei steht

setup.DataFilename = ' ';

setup.DataFilepath = 'D:\A-Data\Paco'; % Hier den Ordner eintragen, in dem nach Daten gesucht werden soll

uicontrol('Style','pushbutton','String','Input-Datei',...

'Units','normalized',...

'Position',[0.05, 0.03, 0.06, 0.03],...

'Callback',@ReadData_Callback);

uicontrol('Style','pushbutton','String','auswΣhlen',... % Zeitbereich auswΣhlen

'Units','normalized',...

'Position',[0.12, 0.03, 0.06, 0.03],...

'Callback',@select_Callback);

uicontrol('Style','pushbutton','String','alles zeigen',... % Zeitbereich auswΣhlen

'Units','normalized',...

'Position',[0.19, 0.03, 0.06, 0.03],...

'Callback',@showall_Callback);

uicontrol('Style','pushbutton','String','<',... % Zeitbereich auswΣhlen

'Units','normalized',...

'Position',[0.26, 0.03, 0.03, 0.03],...

'Callback',@back_Callback);

uicontrol('Style','pushbutton','String','>',... % Zeitbereich auswΣhlen

'Units','normalized',...

'Position',[0.31, 0.03, 0.03, 0.03],...

'Callback',@forward_Callback);

uicontrol('Style','pushbutton','String',' >>',... % next com

'Units','normalized',...

'Position',[0.36, 0.03, 0.03, 0.03],...

'Callback',@next_Callback);

uicontrol('Style','pushbutton','String',' messen',... % next com

'Units','normalized',...

'Position',[0.41, 0.03, 0.06, 0.03],...

'Callback',@messen_Callback);

uicontrol('Style','text','String','Pre-time (min):',... % pretime auswΣhlen

'Units','normalized','BackgroundColor',[0.8 0.8 0.8],...

'Position',[0.75, 0.024, 0.07, 0.03]);

uicontrol('Style','edit','String','1',... % pretime auswΣhlen

'Units','normalized',...

'Position',[0.82, 0.03, 0.03, 0.03],...

'Callback',@pretime_Callback);

uicontrol('Style','text','String','Post-time (min):',... % pretime auswΣhlen

'Units','normalized','BackgroundColor',[0.8 0.8 0.8],...

'Position',[0.86, 0.024, 0.07, 0.03]);

uicontrol('Style','edit','String','1',... % pretime auswΣhlen

'Units','normalized',...

'Position',[0.93, 0.03, 0.03, 0.03],...

'Callback',@posttime_Callback);

uicontrol('Style','pushbutton','String','Markierung versetzen',... % next com

'Units','normalized',...

'Position',[0.55, 0.03, 0.08, 0.03],...

'Callback',@Markierung_versetzen_Callback);

uicontrol('Style','pushbutton','String','Grafik von diesem Abschnitt',... % next com

'Units','normalized',...

'Position',[0.65, 0.03, 0.1, 0.03],...

'Callback',@Grafik_Abschnitt_Callback);

f2 = figure('Position',[20,90,scrsz(3)*0.95, scrsz(4)*0.8],'Toolbar','none',...

'MenuBar','none','vis','off');

uicontrol('Style','pushbutton','String',' weiter ohne speichern',... % next com

'Units','normalized',...

'Position',[0.05, 0.03, 0.2, 0.03],...

'Callback',@weiter_ohne_speichern_Callback);

uicontrol('Style','pushbutton','String',' Anstieg messen',... % next com

'Units','normalized',...

'Position',[0.35, 0.03, 0.2, 0.03],...

'Callback',@anstieg_messen_Callback);

uicontrol('Style','pushbutton','String',' weiter mit speichern',... % next com

'Units','normalized',...

'Position',[0.7, 0.03, 0.2, 0.03],...

'Callback',@weiter_mit_speichern_Callback);

message = uicontrol('Style','text','String','Beginn des Anstiegs markieren',... % pretime auswΣhlen

'Units','normalized','FontSize',12,'vis','off',...

'Position',[0.35, 0.2, 0.3, 0.04]);

% f3 = figure('Position',[40,90,scrsz(3)*0.9, scrsz(4)*0.5],'vis','off');

extra_figures = cell(1,5);

extra_figures{1} = figure('Position',[20,90,scrsz(3)*0.2, scrsz(4)*0.5],'vis','off');

extra_figures{2} = figure('Position',[scrsz(3)*0.23,90,scrsz(3)*0.2, scrsz(4)*0.5],'vis','off');

extra_figures{3} = figure('Position',[scrsz(3)*0.45,90,scrsz(3)*0.2, scrsz(4)*0.5],'vis','off');

extra_figures{4} = figure('Position',[scrsz(3)*0.67,90,scrsz(3)*0.2, scrsz(4)*0.5],'vis','off');

extra_figures{5} = figure('Position',[scrsz(3)*0.89,90,scrsz(3)*0.05, scrsz(4)*0.5],'vis','off');

uicontrol('Style','pushbutton','String',' weiter',... % next com

'Units','normalized',...

'Position',[0.05, 0.4, 0.9, 0.3],...

'Callback',@weiter);

% uicontrol('Style','pushbutton','String','3',... % posttime auswΣhlen

% 'Units','normalized',...

% 'Position',[0.31, 0.03, 0.03, 0.03],...

% 'Callback',@posttime_Callback);

% 'FontSize',ftsize,'FontName','Times','FontWeight','bold',...

% d =

%

% data: [1x34006016 double]

% datastart: [8x2 double]

% dataend: [8x2 double]

% titles: [8x10 char]

% rangemin: [8x2 double]

% rangemax: [8x2 double]

% unittext: 'mN'

% unittextmap: [8x2 double]

% blocktimes: [7.3452e+05 7.3452e+05]

% tickrate: [400 400]

% samplerate: [8x2 double]

% firstsampleoffset: [8x2 double]

% comtext: [17x18 char]

% com: [31x5 double] Spalte 3: Zeit, Spalte 5 Typ (bezieht sich auf comtext)

function ReadData_Callback(~, ~)

[setup.DataFilename,setup.DataFilepath] = uigetfile([setup.DataFilepath,'\*.mat'], 'Select *.mat - file');

d=load(fullfile(setup.DataFilepath,setup.DataFilename));

% Daten werden verkleinert, nur jeder 8. Wert wird genommen, da sonst das

% Programm zu langsam ist

% Zeitachse in Minuten, beginnt fⁿr jeden neuen versuch wieder bei Null

[setup.Nkanal, Nversuch] = size(d.datastart); % Anzahl von KanΣlen und Versuchen

d.data = d.data(1:setup.Nkanal:end);

d.datastart = (d.datastart+7) / setup.Nkanal;

d.dataend = ((d.dataend+setup.Nkanal) / setup.Nkanal) -1;

d.samplerate = mean(d.samplerate) / setup.Nkanal;

d.com(:,3) = round(d.com(:,3)/setup.Nkanal); % Indices an denen ein Kommentar steht Hier als zeitwert in Minuten

data_length = mean(diff(d.datastart)); % LΣnge des Datenvektors eines Kanals in jedem Experiment

% if Nversuch == 2, vers = 2; else vers = 1; end % Hier wird davon ausgegangen, dass immer der letzte Versuch wichtig ist

vers = Nversuch;

setup.samplerate = d.samplerate(vers);

setup.time = (0:data_length(vers)-1) ./ setup.samplerate ./ 60; % time in Min. Samplerate in samples pro s !!!!

an=d.datastart(1,vers); % an und en sind immer Indices, nie Zeitwerte

en = d.dataend(setup.Nkanal,vers);

setup.data = reshape(d.data(an:en)',data_length(vers),setup.Nkanal);

setup.com = d.com(d.com(:,2) == vers,:);

setup.comtext = d.comtext;

setup.pre_post = [1 1 0 0]; % Zeit in Min (1+2) und in datenpunkten (3+4)

setup.pre_post(3:4) = round(setup.pre_post(1:2) .* setup.samplerate .* 60);

setup.an = 1;

setup.en = data_length(vers);

setup.next_comment = 0;

setup.titles = d.titles;

setup.chan = 0; % aktueller Kanal, der gemessen wird

erg = zeros(10,setup.Nkanal); % Ergebnisse, fⁿr jees ereignis ein Ergebnisblock, wird als textfile gespeichert

upper_freq_limit=0.2;

[b,a]=butter(3,upper_freq_limit*2/d.samplerate(vers));

upper_freq_limit=2 ;

[b2 a2]=butter(3,upper_freq_limit*2/d.samplerate(vers));

axes('position', [0 0 1 1],'visible','off')

txt = sprintf('File: %s Gesamtdauer: %5.2f Min. ', setup.DataFilename, setup.time(end));

text(0.5 ,0.99,txt,'units','normalized','horizontalAlignment','center','VerticalAlignment','top','Fontsize',12)

fprintf(' \n\n')

[r,~] = size(setup.comtext);

for n=1:r

fprintf('%d) %s \n',n,setup.comtext(n,:))

end

fprintf(' \n\n')

setup.com

plot_all

end

function select_Callback(~,~)

[x,~] = ginput(2);

[~,setup.an] = min(abs(setup.time-x(1)));

[~,setup.en] = min(abs(setup.time-x(2)));

plot_all

end

function showall_Callback(~,~)

setup.an = 1;

setup.en = length(setup.time);

setup.next_comment = 0;

plot_all

end

function back_Callback(~,~)

dummy = setup.en - setup.an;

setup.an = setup.an - dummy;

if setup.an < 1

setup.an = 1;

end

setup.en = setup.an + dummy;

plot_all

end

function forward_Callback(~,~)

dummy = setup.en - setup.an;

setup.an = setup.an + dummy;

setup.en = setup.an + dummy;

if setup.en > length(setup.time)

setup.en = length(setup.time);

setup.an = setup.en - dummy;

end

plot_all

end

function pretime_Callback(src,~)

setup.pre_post(1) = str2double(get(src,'String'));

setup.pre_post(3) = round(setup.pre_post(1) .* setup.samplerate .* 60);

setup.an=setup.com(setup.next_comment,3) - setup.pre_post(3); % index begin next window

plot_all

end

function posttime_Callback(src,~)

setup.pre_post(2) = str2double(get(src,'String'));

setup.pre_post(4) = round(setup.pre_post(2) .* setup.samplerate .* 60);

setup.en=setup.com(setup.next_comment,3) + setup.pre_post(4); % index begin next window

plot_all

end

function next_Callback(~,~) % go to next comment/event

if setup.next_comment < length(setup.com)

setup.next_comment = setup.next_comment + 1;

setup.an=setup.com(setup.next_comment,3) - setup.pre_post(3); % index begin next window

setup.en=setup.com(setup.next_comment,3) + setup.pre_post(4); % index begin next window

fprintf('setup.next_comment = %d; setup.an = %d\n', setup.next_comment, setup.an)

else

setup.an = 1;

setup.en = length(setup.data);

setup.next_comment = 0; % reset

end

plot_all

t=setup.com(setup.next_comment,5); % typ comment

axes('position', [0 0 1 1],'visible','off');

txt = sprintf('Ereignis Nr %d/%d Typ %d (%s)', setup.next_comment, length(setup.com),t,deblank(setup.comtext(t,:)));

text(0.5 ,0.96,txt,'units','normalized','horizontalAlignment','center','VerticalAlignment','top','Fontsize',10)

end

% function Markierung_versetzen_Callback(~,~)

% marker_ind = find((setup.com(:,3) > setup.an) & (setup.com(:,3) < setup.en));

% if length(marker_ind) == 1

% [x,~] = ginput(1);

% [~,ind] = min(abs(setup.time-x));

% % fprintf('Neuer Marker bei %0.6f \n',setup.time(ind))

% setup.com(marker_ind,3) = ind;

% plot_all

% end

% end

function Markierung_versetzen_Callback(~,~)

[x,~] = ginput(1); % der erste click geht auf die Markierung, die ersetzt werden soll

[~,ind] = min(abs(setup.time-x)); % ind ist der Index

[~,marker_ind] = min(abs(setup.com(:,3)-ind)); % ind ist jetzt der Index des Markers

[x,~] = ginput(1); % jetzt auf die neue Position klicken

[~,ind] = min(abs(setup.time-x));

setup.com(marker_ind,3) = ind; % jetzt hat der Marker eine neue Position

plot_all

end

function Grafik_Abschnitt_Callback(~,~)

an = setup.an;

en = setup.en;

ti = (0:(setup.en - setup.an)) ./ setup.samplerate ./ 60;

t=setup.com(setup.next_comment,5);

for n=1:4

set(extra_figures{n},'vis','on')

subplot(2,1,1)

plot(ti,setup.data(an:en,n),'k','LineWidth',1.25)

axis([ti(1) ti(end) min(setup.data(an:en,n)) min(setup.data(an:en,n)) + 1.2 * (max(setup.data(an:en,n)) - min(setup.data(an:en,n)))]);

ylabel('[ mN ]')

set(gca,'box','off','TickDir','out','LineWidth',1.25)

title(setup.titles(n,:))

t1 = deblank(setup.comtext(t,:));

ind = strfind(t1,'-');

if ~isempty(ind)

t1 = [t1(1:ind-1),'^{', t1(ind:ind+2),'}',t1(ind+3:end)];

end

txt = sprintf('%s, Control', t1);

text(0.5,0.9,txt,'HorizontalAlignment','center','units','normalized','FontName','Arial')

subplot(2,1,2)

plot(ti,setup.data(an:en,n+4),'k','LineWidth',1.25)

axis([ti(1) ti(end) min(setup.data(an:en,n+4)) min(setup.data(an:en,n+4)) + 1.2 * (max(setup.data(an:en,n+4)) - min(setup.data(an:en,n+4)))]);

ylabel('[ mN ]')

set(gca,'box','off','TickDir','out','LineWidth',1.25)

xlabel('Time [ Min ]')

txt = sprintf('%s, 5mg/kg rotenone', t1);

text(0.5,0.9,txt,'HorizontalAlignment','center','units','normalized','FontName','Arial')

end

set(extra_figures{5},'vis','on')

end

function messen_Callback(~,~) % missst einen Kanal aus

setup.chan = setup.chan + 1;

if setup.chan > setup.Nkanal

setup.chan = 0;

erg(:,:) = 0; % Ergebnisse resetten

set(f2,'vis','off')

else % jetzt ausmesssen

an = setup.an;

en = setup.en;

marker_ind = setup.com(setup.next_comment,3); % Index des markers, um den gemessen wird

set(f2,'vis','on')

plot(setup.time(an:en),setup.data(an:en,setup.chan)) % Daten plotten

set(gca,'position', [0.06 0.3 0.9 0.6])

axis tight

ax=axis; % vertikale Linie bei Ereignis

y = ax(3) + (ax(4)-ax(3)) * 0.85;

line([setup.time(marker_ind) setup.time(marker_ind)],[ax(3) ax(4)])

text(setup.time(marker_ind), y, num2str(setup.com(setup.next_comment,5)))

title([num2str(setup.chan),'.) ', setup.titles(setup.chan,:)])

xlabel('Zeit [ Min ]')

temp_data = filtfilt(b,a,setup.data(an:en,setup.chan)); % mittelwerte rot plotten

line(setup.time(an:en),temp_data,'color',[1,0,0],'linewidth',1.5)

temp_data = filtfilt(b2,a2,setup.data(an:en,setup.chan)); % Minima und Maxima finden

temp_data=diff(temp_data);

ind1 = find(temp_data>0);

ind2 = find(diff(ind1) > 1);

maxima = ind1(ind2)+1+an-1; % indices beziehen sich auf setup.data

minima = ind1(ind2+1)+an-1; % indices beziehen sich auf setup.data

ind1 = find(minima <= marker_ind); % indices maxima und minima prae

ind2 = find(minima > marker_ind); % indices maxima und minima post

hold on

plot(setup.time(maxima(ind1)),setup.data(maxima(ind1),setup.chan),'ro')

plot(setup.time(minima(ind1)),setup.data(minima(ind1),setup.chan),'ro')

plot(setup.time(maxima(ind2)),setup.data(maxima(ind2),setup.chan),'go')

plot(setup.time(minima(ind2)),setup.data(minima(ind2),setup.chan),'go')

hold off

erg(setup.chan,1) = mean(setup.data(maxima(ind1),setup.chan) - setup.data(minima(ind1),setup.chan)); % Erg 1 : mean Amplitude prae

erg(setup.chan,2) = mean(setup.data(maxima(ind2),setup.chan) - setup.data(minima(ind2),setup.chan)); % Erg 2 : mean Amplitude post

erg(setup.chan,3) = max(setup.data(maxima(ind1),setup.chan)); % Erg 3 : Maximum prae

erg(setup.chan,4) = min(setup.data(minima(ind1),setup.chan)); % Erg 4 : Minimum prae

erg(setup.chan,5) = max(setup.data(maxima(ind2),setup.chan)); % Erg 5 : Maximum post

erg(setup.chan,6) = min(setup.data(minima(ind2),setup.chan)); % Erg 6 : Minimum post

erg(setup.chan,7) = length(ind1) / (setup.time(marker_ind) - setup.time(an)) ; % Erg 7 : Frequenz prae in Kontraktionen / Minute

erg(setup.chan,8) = length(ind2) / (setup.time(en) - setup.time(marker_ind)) ; % Erg 8 : Frequenz post in Kontraktionen / Minute

fprintf('Messwerte: %10.3f /n',erg(setup.chan,1:8))

end

end

function weiter_ohne_speichern_Callback(~,~)

messen_Callback;

end

function weiter(~,~)

for n=1:5

set(extra_figures{n},'vis','off');

end

% plot_all;

end

function weiter_mit_speichern_Callback(~,~)

messen_Callback;

end

function anstieg_messen_Callback(~,~) % Beginn und Ende des steilen Anstiegs nach Ereignis soll markiert werden

an = setup.an;

en = setup.en;

temp_data = filtfilt(b,a,setup.data(an:en,setup.chan)); % mittelwerte rot plotten

set(message,'vis','on','string','Beginn des Anstiegs markieren');

[x,~] = ginput(1);

[~,ind1] = min(abs(setup.time-x)); ind1=ind1-an+1; % ind1 (Beginn des Anstiegs) bezieht sich jetzt auf temp_data

set(message,'vis','on','string','Maximum des Anstiegs markieren');

[x,~] = ginput(1);

[~,ind2] = min(abs(setup.time-x)); ind2=ind2-an+1;

set(message,'vis','on','string','Ende und Plateau des Abfalls markieren');

[x,offset] = ginput(1);

[~,ind3] = min(abs(setup.time-x)); ind3=ind3-an+1;

set(message,'vis','off');

x=setup.time((ind1:ind2)+an-1); x = x(:);

y = temp_data(ind1:ind2); y=y(:);

const = ones(ind2-ind1+1,1);

Coeffs = [x,const] \ y; %Fit line to Anstieg

erg(setup.chan,9) = Coeffs(1); % Anstigssteilheit

% erg(setup.chan,10) = Coeffs(2);

y = x .* Coeffs(1) + Coeffs(2);

line(x,y,'color',[0 0 0],'linewidth',1.5)

erg(setup.chan,11) = temp_data(ind2) - temp_data(ind1); % Anstiegsamplitude

% Hinten muss eine Nullinie sein zum fitten der e-funktion

y = temp_data(ind2:ind3)-offset; y=y(:); % abfallende Flanke mit exponential fit

x = setup.time(1:length(y));x=x(:); % Zeitachse

ff = fit(x,y,'exp1');

% plot(ff,x,y)

fit_data = ff.a * exp(ff.b*x);

line(setup.time((ind2:ind3)+an-1),fit_data + offset,'color',[0 0 0],'linewidth',1.5)

% [~,~] = ginput(1);

end

function plot_all

an = setup.an;

en = setup.en;

marker_ind = find((setup.com(:,3) > an) & (setup.com(:,3) < en)); % index an denen ein event ist

fprintf('an = %d; en = %d\n',an,en)

for n=1:setup.Nkanal

subplot(setup.Nkanal/2,2,n)

plot(setup.time(an:en),setup.data(an:en,n))

axis tight

ax=axis;

y = ax(3) + (ax(4)-ax(3)) * 0.85;

if ~isempty(marker_ind)

for n2=marker_ind'

line([setup.time(setup.com(n2,3)) setup.time(setup.com(n2,3))],[ax(3) ax(4)])

text(setup.time(setup.com(n2,3)), y, num2str(setup.com(n2,5)))

end

end

title([num2str(n),'.) ', setup.titles(n,:)])

if n > (setup.Nkanal-2)

xlabel('Zeit [ Min ]')

end

end

axes('position', [0 0 1 1],'visible','off');

txt = sprintf('File: %s Gesamtdauer: %5.2f Min.', setup.DataFilename, setup.time(end));

text(0.5 ,0.99,txt,'units','normalized','horizontalAlignment','center','VerticalAlignment','top','Fontsize',12)

end

end

% Atomzerfall:

%

% N=N0 * exp(-lambda*t)

% hier

% y=b + N0 * exp(-lambda*(t-t0))

% Matlab:

% y = a * exp(bx)
